# Cross‐Sectional Models of Groundwater Flow: Review and Correction for Transverse Flow

**DOI:** 10.1111/gwat.70017

**Published:** 2025-09-03

**Authors:** Amin Gholami, Amir Jazayeri, Adrian D. Werner

**Affiliations:** ^1^ College of Science and Engineering Flinders University GPO Box 2100 Adelaide South Australia 5001 Australia; ^2^ National Centre for Groundwater Research and Training Flinders University GPO Box 2100 Adelaide South Australia 5001 Australia

## Abstract

Cross‐sectional (2D) groundwater models are commonly applied to simulate complex processes that are challenging to capture using the coarse grids of 3D regional‐scale models. 2D models are often extracted from 3D models for this purpose. However, translating groundwater properties from 3D to 2D models so that regional flow patterns are preserved poses several challenges. A methodology is presented here to maximize agreement between the heads of 2D and 3D groundwater models, considering MODFLOW models with rectilinear grids. This includes careful averaging of hydraulic properties and stresses from the 3D model to create commensurate properties and stresses in cross section. The approach was evaluated by examining the statistical match of transient heads within 10 cross sections extracted from a 3D model of the Limestone Coast (Australia). Concordance between 2D and 3D models was generally poor but was improved by incorporating lateral flow as inflows/outflows in 2D models. Lateral flows required inputs from the 3D model, which limits the application of 2D models as independent predictive tools. Pumping in the 3D model was redistributed to neighboring cells to reduce errors in the 2D model that arise from the limited capability to simulate pumping effects. Although pumping redistribution led to minimal improvement for the case study model, simpler modeling scenarios with more intense, localized pumping showed substantially better head matches between 2D and 3D models when pumping redistribution was applied. The methodology for creating cross‐sectional models offered in this article provides relatively simple steps for creating 2D models that are consistent with 3D parent models, although further work is needed to develop a methodology for 2D models that are oblique to 3D model grids.

## Introduction

Despite major advances in computing speeds and numerical methods (e.g., Li et al. 2020), the regional‐scale simulation of complex groundwater phenomena (e.g., tides, multi‐phase flow, fine‐scale heterogeneities, etc.) remains a challenging undertaking using 3D models. This arises from the need to simulate numerically demanding processes over large scales using grids of limited spatial resolution, creating high computational burdens (Kerrou and Renard 2010). To overcome this challenge, 2D models are often constructed, either in the horizontal or vertical planes. These models neglect flow in the third dimension—for example, horizontal flow that is perpendicular to a vertical cross‐sectional model, or vertical flow in the case of a vertically integrated (horizontal plane) model (Anderson et al. 2015). Examples of 2D models include studies of coastal aquifers (e.g., Werner et al. 2013), tidal lake‐aquifer interaction (e.g., Jazayeri et al. 2021a), variably saturated flow (e.g., Koohbor et al. 2020), river‐aquifer interaction (e.g., Werner 2017; Jazayeri et al. 2020, 2021b), and multi‐phase flow (e.g., Prieto‐Espinoza et al. 2023), amongst a wide range of other research applications.

Whether or not a 2D model is a reasonable predictor of groundwater flow (and solute transport in many cases) relative to the 3D variability that exists in real‐world systems is commonly questioned. For example, Burnett and Frind (1987) highlighted the impact of dimensionality on solute transport model predictions when transitioning from 3D to 2D representations. Their findings demonstrated that the simulated plume from a 2D vertical cross section advanced to a greater extent than the reference 3D plume simulation. This occurred because the spreading of the solute plume in the 3D model occurred in the direction perpendicular to the cross‐sectional model axis, but this was lost in the 2D representation. Kerrou and Renard (2010) evaluated the influence of heterogeneity and dimensionality on salt water intrusion using both 2D and 3D models, where the latter was a 3D extraction of the anisotropic, dispersive Henry problem of Abarca et al. (2007). They adjusted the hydraulic conductivity field in the 2D (cross‐sectional) model to effectively mimic 3D salt water intrusion in heterogeneous aquifers. The impact of heterogeneity on salt water intrusion was found to vary significantly between 2D and 3D models, both in magnitude and in general trends. For example, in 2D models, the penetration length of the salt water toe diminished as the level of random heterogeneity increased. The 3D models revealed varying patterns, with the length of toe penetration either increasing or decreasing, depending on the level of heterogeneity and anisotropy.

Yu and Michael (2019) conducted cross‐sectional modeling to study the impact of aquifer heterogeneity similar to that found in the Lower Bengal Delta (Bangladesh). They compared the impact of 100 years of pumping on groundwater salinization in 3D and 2D models, reporting the salinized area, seawater intrusion rate (i.e., the speed that seawater encroached landward in the aquifer) and the time required for salt water to reach pumping wells. 2D simulations generally showed slightly larger salinized areas and higher seawater intrusion rates compared to 3D simulations, including in both homogeneous and heterogeneous scenarios. Knight et al. (2021) compared 2D and 3D models in their investigation of offshore fresh groundwater circulation resulting from alongshore head gradients. The 3D seawater intrusion models were constructed based approximately on the conditions of the Limestone Coast (Australia). They compared the freshwater–salt water interface and freshwater flow rates of 3D and cross‐sectional models and found that in cases with alongshore hydraulic gradients, 2D approaches overestimate the offshore extent of fresh groundwater where onshore heads are highest and underestimate it where onshore heads are lowest. This arose because lateral (alongshore) flow perpendicular to cross‐section alignments influenced the position of the freshwater–seawater interface. Geng and Michael (2021) also compared 2D (cross‐sectional) and 3D models in their study of alongshore groundwater flow in heterogeneous coastal aquifers of Hawai'i Island (Hawaii). They obtained the submarine groundwater discharge (SGD) of three statistically equivalent 3D models (each with alternative representations of aquifer heterogeneity) with the SGD obtained from corresponding 2D models. Their findings revealed that 2D models computed lower rates of SGD compared to 3D models when heterogeneities in the form of conduits were included, whereas the absence of conduits led to similar rates of SGD in both 3D and 2D models.

The extraction of a cross‐sectional model from a 3D model (i.e., for the purposes of reducing the numerical impost of simulation) faces several challenges, especially when the cross section is oblique to the 3D model grid. This requires careful translation of parameters and stresses from 3D to 2D due to several factors. For example, 2D models should include only a fraction of some 3D stresses, including where the 2D model axis only partially intercepts a 3D model cell. Also, aquifer properties from the 3D model need to be averaged (using an averaging approach that depends on the parameter) in translating these to 2D models. Additionally, the effects of well pumping (and the accompanying radial flow) in 3D models are difficult to capture in cross‐sectional models. Whether or not a cross‐sectional model is a proper representation of its parent 3D model requires comparison of their respective outputs; however, this is rarely reported. For example, the authors were unable to find evidence of such a comparison between a 3D model and a cross‐sectional model extracted at an oblique angle to the cells of the 3D model.

This paper presents a structured workflow for extracting cross sections from a 3D MODFLOW model with a rectilinear grid, along with efforts to improve the reproduction (in the cross‐sectional model) of heads from the parent, 3D model. As cross sections may be aligned with the rows and columns of the rectilinear grid of the 3D model or may be oblique to them, the methodology requires various formulations for averaging hydraulic properties and stresses in the 3D model for application to 2D models. The analysis includes testing of different approximations of pumping that are usually excluded from cross‐sectional models because the associated radial flow cannot be simulated. Furthermore, the impact of incorporating lateral flow (i.e., flow in the 3D model that is perpendicular to the cross‐sectional model axis) into the 2D model is examined, at least for cross sections that align with the rows and columns of the rectilinear grid of a regional‐scale 3D model.

## Methodology

This section outlines the procedure for extracting a cross section from a rectilinear 3D groundwater model. The cross sections were obtained from a 3D MODFLOW model of the Lower Limestone Coast (Australia) that simulates transient stresses for the period 1970 to 2013 (Morgan et al. 2015). The components of the water balance, including storage, specified‐head boundaries, wells, drains, evapotranspiration, and recharge, are presented in Table S1 of the Supporting Information. The approach involves the translation of the following model components from the 3D model to the cross‐sectional model: (1) model grid, (2) aquifer hydraulic properties, (3) initial and specified heads, and the distribution of active and inactive cells, (4) flux‐based stresses (recharge and pumping), and (5) head‐dependent stresses (evapotranspiration and drains). The workflow was developed using FloPy, a Python package for creating, running, and post‐processing MODFLOW‐based models (Bakker et al. 2016; Leaf and Fienen 2022; Hughes et al. 2024).

**Figure 1:**
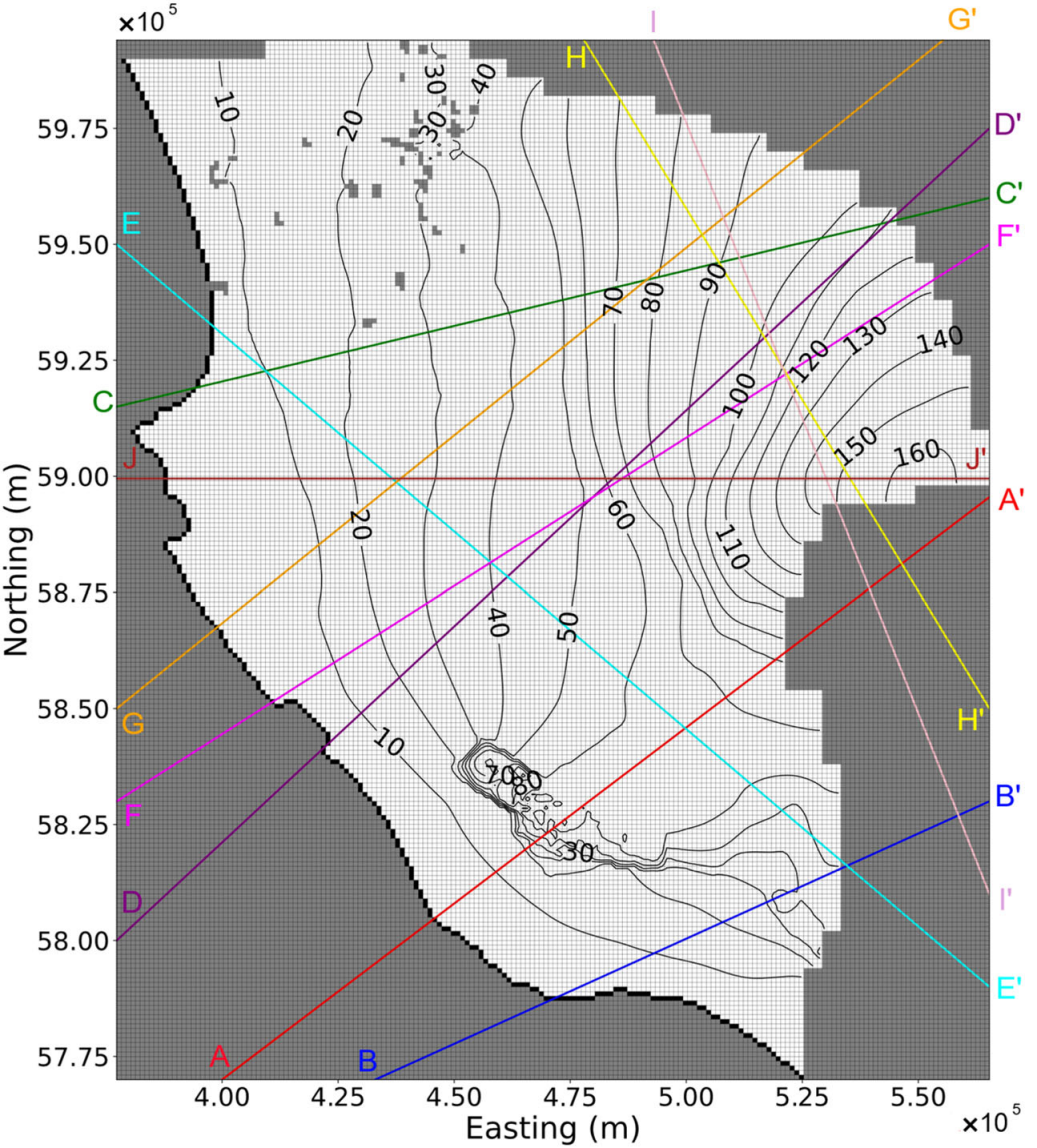
Locations of cross sections through the 3D model of the Lower Limestone Coast (Morgan et al. 2015). Black contours represent head, white‐filled cells are active, black‐filled cells indicate specified‐head cells, and gray‐filled cells are inactive. Contours of head from layer 1 and at the end of the first stress period (10 days of the 16,081‐day simulation) are shown. Contour labels represent head in meters above AHD (Australian Height Datum).

The methodology for transferring each of the above‐mentioned model components is described below. These were combined to extract 10 cross sections (from the 3D regional model) that were subsequently run to compare to the heads of the parent model. Figure 1 shows the location of these cross sections and the outline of the 3D model, which was created by Morgan et al. (2015). Cross sections were selected to avoid disconnected regions of the 3D model domain (e.g., as would occur if a north–south cross section was adopted at an easting of 530,000 m). The cross sections represented a mixture of alignments relative to the groundwater flow direction, with several being strongly oblique to the head contours and/or crossing areas showing complex head patterns (e.g., see cross section AA′).

### Model Grid

**Figure 2:**
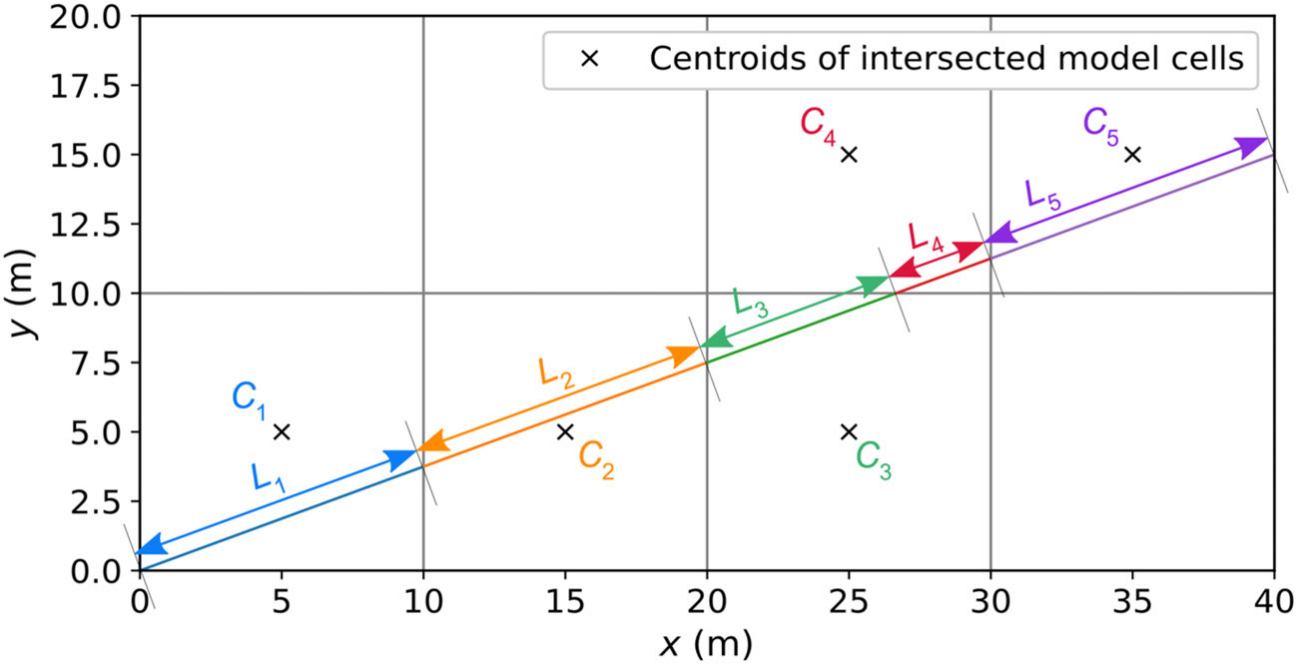
Plan view of a cross‐sectional model extracted obliquely from a 3D model with a grid consisting of two rows (in the *y*‐direction) and four columns (in the *x*‐direction), where *L*_1_ to *L*_5_ are the lengths of cross‐sectional model cells obtained from the intersection of 3D model cells *C*_1_ to *C*_5_, respectively.

The usual practice of selecting the alignment of a cross‐sectional model (presumed to have a straight‐line axis in the current study) is to intersect head contours at right angles. This is difficult in the case of transient, real‐world simulations because cross sections have a stationary position while head contours invariably shift in time. Nevertheless, lateral flow (i.e., perpendicular to the axis of the cross section) is minimized using this approach. Usually, the resulting cross sections are oblique to the 3D model grid. A cross section passing through a rectilinear grid at an oblique angle will intersect non‐uniform portions of 3D cells. This may create irregular cell sizes in the 2D model if the intersected 3D cells are each represented individually in the 2D model, leading to numerical challenges if adjacent cells have contrasting dimensions. Figure 2 shows how the cells of a cross‐sectional model will have varying lengths when aligned obliquely with a simple grid.

The technique adopted in the current study for extracting the intersected lengths of 3D model cells, shown in Figure 2, involved the *GridIntersect* class in the FloPy package (Hughes et al. 2024). Initially, non‐uniform 2D cell lengths were adopted, whereby lengths *L*_1_, *L*_2_, and *L*_5_ are equal, while *L*_3_ and *L*_4_ differed (but sum to *L*_1_). Non‐uniform cell lengths with ratios of neighboring cells that exceed recommended limits potentially create numerical errors. For example, Anderson et al. (2015) recommend restricting the expansion of cell lengths to a factor of 1.5. To avoid this issue, cells in the 2D model were merged so that the cell interval of the 3D model was effectively preserved, notwithstanding that the intersection length of an oblique cross‐sectional grid exceeds the rectilinear cell dimensions of the 3D model (i.e., in Figure 2, *L*_2_ exceeds the dimensions of cell *C*_2_ according to Pythagoras). Thus, the length of each cross‐sectional cell was set to *L*_c_ (L), given by:

(1) $$L_{c}=\left\{\begin{array}{l} \sqrt{{\left(\Delta x\right)}^{2}+{\left(s\Delta x\right)}^{2}}\;\;\text{if}\;s\leq 1\\ \sqrt{{\left(\Delta y\right)}^{2}+{\left(\frac{\Delta y}{s}\right)}^{2}}\;\text{if}\;s>1 \end{array}\right.$$

where *s* (−) represents the slope (*∆y*/*∆x*) of the cross‐section axis relative to the rectilinear grid, *∆x* (L) is the width of a column in the 3D model, and *∆y* (L) is the width of a row in the 3D model. Here, the method is described for uniform cell sizes in the 3D model. The approach requires minor adjustments to accommodate 3D grid with variable cell sizes. In Figure 2, *s* is $\sqrt{\left(L_{1}^{2}-10^{2}\right)}/10$, where the 3D model column width is a uniform 10 m. The unavoidable aggregation of 3D cells in building the 2D model grid led to the need for appropriate averaging methods for transferring 3D model properties (hydraulic parameters, boundary conditions, stresses, etc.) to the 2D model, as discussed in the next subsection.

For cross‐sectional models aligned to the orientation of a 3D rectilinear model, the top and bottom elevations of the 2D cells can be directly taken from the corresponding 3D model cells. This is also the case for the cells of oblique cross‐sectional models where the cross section passes through the entire width of a 3D cell (e.g., *C*_1_, *C*_2_, and *C*_5_; Figure 2) and thus merging of 3D cells is not required. Where 2D cells result from the merging of two 3D cells, their top and bottom elevations were determined using distance‐weighted averaging, as:

(2) $$P_{m}=\frac{P_{i}L_{i}+P_{i+1}L_{i+1}}{L_{i}+L_{i+1}}$$

where *L*_*i*_ (L) and *L*_*i*+1_ (L) are the intersection lengths (e.g., *L*_3_ and *L*_4_ in Figure 2) of two neighboring 3D cells that were merged in the 2D model, *P*_*i*_ and *P*_*i*+1_ are the model properties (e.g., the top or bottom elevations of model cells) linked to these cells, and *P*_m_ is the property assigned to the merged cell in the 2D model.

### Aquifer Hydraulic Properties

Where 2D model cells do not represent the merger of two 3D model cells, aquifer properties in the 2D model, such as the horizontal hydraulic conductivity (*K*_h_ [L T^−1^]), the vertical hydraulic conductivity (*K*_v_ [L T^−1^]), specific storage (*S*_s_ [L^−1^]) and specific yield (*S*_y_ [−]), were taken directly from the 3D model. For merged 2D cells, Equation (2) was used to calculate the cross‐sectional values of *K*_v_, *S*_s_, and *S*_y_. Averaging of the *K*_h_ for merged cells took into consideration the method used to average *K*_h_ between adjacent cells in MODFLOW, namely the harmonic mean, treating the neighboring cells as porous media that are connected in series (Langevin et al. 2017). This led to the following averaging formula:

(3) $$K_{h,m}=\frac{L_{i}+L_{i+1}}{\left(\frac{L_{i}}{K_{h,i}}\right)+\left(\frac{L_{i+1}}{K_{h,i+1}}\right)}$$

where *L*_*i*_ and *L*_*i*+1_ are the lengths of two neighboring 3D cells that were merged in the 2D model, *K*_h,*i*_ (L T^−1^) and *K*_h,*i+*1_ (L T^−1^) are the horizontal hydraulic conductivities of the corresponding cells, and *K*_h,m_ (L T^−1^) is the horizontal hydraulic conductivity of the merged cell in the 2D model. Isotropic hydraulic conductivity was assumed in this study. In real‐world applications where anisotropy exists (e.g., differing horizontal hydraulic conductivities in the *x*‐direction (*K*_h,*x*_) and *y*‐direction (*K*_h,*y*_), where *K*_h,*x*_ ≠ *K*_h,*y*_) and the cross‐sectional orientation is oblique to the principal directions of the hydraulic conductivity tensor, it would be necessary to project and rotate the tensor accordingly.

### Initial Heads, Specified Heads and Active/Inactive Conditions

Initial heads, specified‐head boundary conditions, and active/inactive cell conditions for 2D model cells that are not the combination of two 3D model cells were the same as the 3D model. Here, “inactive” refers to no‐flow cells that are omitted from MODFLOW calculations of head and flux, while “active” refers to cells that are included in the simulation, where groundwater flow is computed and heads are updated throughout the model run (i.e., not fixed using a specified‐head condition). Table 1 provides the method for assigning initial heads to the 2D model for merged cells. Table 1 also describes the rules for setting the conditions (active, inactive or specified head) of merged cells in the 2D model.

**Table 1:** Strategies for Specifying the Active (i.e., Head Is Not Specified by the User), Inactive (i.e., Cells That Are Excluded from MODFLOW Calculations), or Specified‐Head Conditions of Merged Cells in the 2D Model

Case	*C*_*i*_	*C*_*i*+1_	*C*_m_	Calculation of Initial and/or Specified Heads in the 2D Model
1	Active	Active	Active	Equation (2)
2	Inactive	Inactive	Inactive	Not required
3	Active	Specified head	Specified head	*C*_*i*+1_
4	Inactive	Active	Active	*C*_*i*+1_
5	Inactive	Specified head	Specified head	*C*_*i*+1_
6	Specified head	Active	Specified head	*C*_*i*_
7	Specified head	Specified head	Specified head	Equation (2)
8	Active	Inactive	Active	*C*_*i*_
9	Specified head	Inactive	Specified head	*C*_*i*_

Notes: *C*_*i*_ and *C*_*i*+1_ are the neighboring 3D cells involved in the creation of the merged cell (*C*_m_) in the 2D model. The methods for determining initial and/or specified heads in the 2D model are also listed.

### Aquifer Stresses (Pumping Wells, Recharge, Evapotranspiration, and Drains)

Radial flow cannot be simulated in 2D cross‐sectional models, requiring careful consideration of the transfer of well pumping rates from the 3D model to the 2D model. If the pumping rate in a 3D model is transferred directly to a 2D model (i.e., where the cross section intercepts a pumping cell), the effect of pumping is greater in the 2D model because lateral flow to the well (perpendicular to the cross‐section axis) is neglected. Equally, if a cross section is one cell departed from a pumping well in the 3D model, and only wells that are intercepted by the 2D model are transferred between the models, the effect of the well is ignored in the 2D model. Thus, we presume that wells that are intercepted by the cross section should be assigned a lower pumping rate in the 2D model than in the 3D model, and wells that are close to, but not intercepted by, the cross section should be included in the 2D model with a pumping rate that is lower than occurs in the 3D model. This strategy had the effect of reducing drawdown in the 2D model that would otherwise be caused by pumping (at the same rate as occurs in the 3D model) from wells intercepted by the cross section, while partial representation of wells was included in areas where the 2D model likely encounters the cone of depression of a well (in the 3D model) that is not intercepted by the 2D model axis. The modified pumping rate in a 2D cross section to produce the same effect as occurs under the radial flow of a 3D model is not easily determined, and so testing of various approaches was undertaken. This also helps to compensate for the approximate nature of the tested methods for transferring pumping from 3D to 2D.

**Figure 3:**
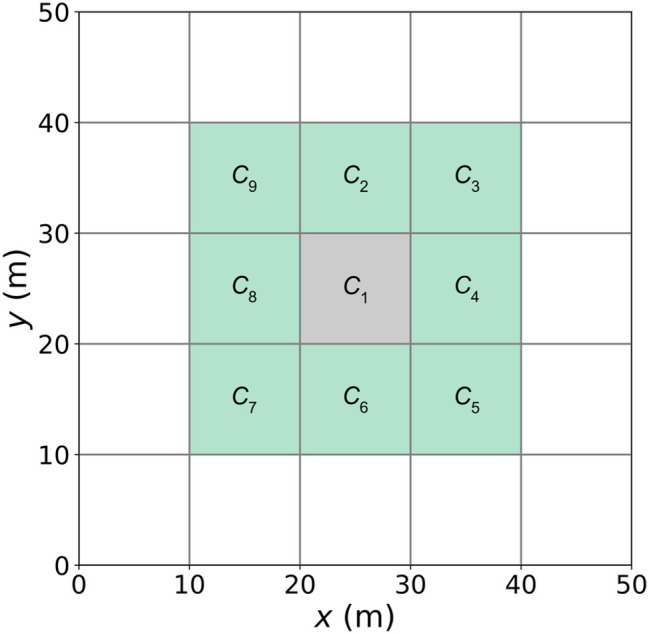
Plan view of the cells in a 3D grid where the pumping rate has been spread across nine cells (Scenario (b); nine‐cell redistribution). *C*_1_ (gray cell) represents the original well cell in the 3D model, while *C*_2_ to *C*_9_ (green cells) are the surrounding cells over which pumping is distributed. The pumping rate in each of the nine cells is equal to 1/9th of the original pumping rate.

The approach for transferring wells from the 3D model to the 2D model firstly involved the redistribution of pumping in the 3D model, whereby each pumping cell was redistributed over neighboring cells using five scenarios: (a) no redistribution – well cells in the 3D model that were intercepted by the 2D model were transferred directly, (b) nine‐cell redistribution—the pumping rate in the 3D model was distributed to nine cells (the pumping cell and 8 neighboring cells, see Figure 3), with each cell assigned 1/9th of the original pumping rate, (c) 49‐cell redistribution (as (b) except with the pumping rate distributed to 49 cells), (d) 121‐cell redistribution, and (e) 361‐cell redistribution. Where the redistribution of pumping encountered inactive or specified‐head cells, these were discounted from the redistribution. The term “pumping wells” is used throughout to refer to extraction, although the proposed approach is equally applicable to injection wells.

The 2D cells that translate directly from the 3D model (i.e., cell amalgamation was not needed) adopted the same well pumping (based on the distributed‐pumping rates, as obtained using the method described above) and recharge fluxes. For merged cells, pumping and recharge fluxes were obtained using Equation (2), as were evapotranspiration parameters (surface elevation, maximum evapotranspiration flux, extinction depth) and the conductance of drain cells. The assignment of drain elevations (*z*_d_ [L]) to the 2D model adopted the methods outlined in Table 2.

**Table 2:** Method for Determining the Drain Elevation (*z*_d_) of Merged Cells in the 2D Model

Case	*C*_*i*_	*C*_*i*+1_	*C*_m_
1	*z*_d,*i*_	None	*z*_d,*i*_
2	None	*z*_d,*i*+1_	*z*_d,*i*+1_
3	None	None	None
4	*z*_d,*i*_	*z*_d,*i*+1_	Equation (2)

Notes: *C*_*i*_ and *C*_*i*+1_ are the neighboring 3D cells involved in the creation of the merged cell (*C*_m_) in the 2D model. *z*_d,*i*_ and *z*_d,*i*+1_ are the drain elevations of cell *C*_*i*_ and *C*_*i*+1_, respectively. “None” means that the 3D model cell does not include a drain.

Figure S1 illustrates the workflow for converting a 3D groundwater model into a 2D cross‐sectional model.

### Accounting for Lateral Flow in 2D Models

**Figure 4:**
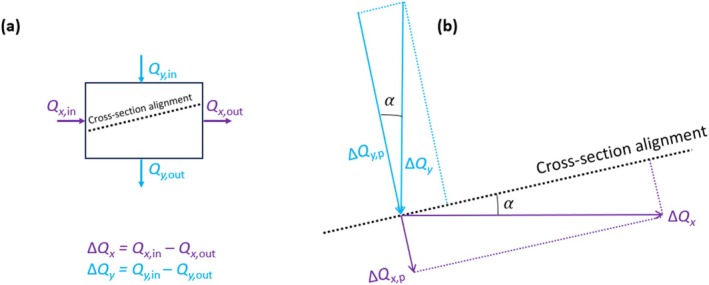
(a) Flow components for a 3D model cell intersected by an oblique cross section, where *Q* represents a volumetric flux (L^3^ T^−1^), subscripts *x* and *y* indicate the flow direction, and subscripts “in” and “out” refer to inflow and outflow to the cell, and (b) Flow components (associated with a 3D model cell) perpendicular to the cross section, where subscript “p” identifies the net flow components (Δ*Q*_*x*,p_ and Δ*Q*_*y*,p_) that are perpendicular to the cross‐section axis.

Lateral flow is a well‐known source of disparity between 3D and 2D models (e.g., Geosyntec Consultants, Inc. 2017). That is, where lateral flows in the 3D model create a net flux into or out of the 2D domain (e.g., representing a cross section to be extracted from the 3D model), the heads of the 3D model are unlikely to be reproduced by the 2D model. Where the cross‐section axis is parallel to the rectilinear grid of a 3D model, the estimation of net flows in the 3D model perpendicular to the cross section is the simple summation of lateral cell fluxes (in the 3D model) perpendicular to the 2D model axis. An evaluation of the net fluxes into or out of a 2D cross section that is oblique to 3D model cells is more complicated, requiring calculation of flow components (in directions perpendicular to the cross‐section axis) from the 3D model. A conceptual model showing the components of lateral flow for an oblique 2D model is shown in Figure 4, which illustrates the situation where the oblique cross section is contained within a single cell of the 3D model. The cross‐sectional model in Figure 4 is depicted only by its centerline. If the cell dimensions of the cross‐sectional model are considered (including perpendicular to the 2D model axis), the net lateral flow components require more complicated geometric calculations to account for the proportion of the 2D cells contained in each 3D cell.

Lateral flows in the case study applications of the current study were derived from the cell budget file of MODFLOW for the 3D model, including input and output flows in the *x*‐direction (*Q*_*x*,in_ [L^3^ T^−1^] and *Q*_*x*,out_ [L^3^ T^−1^]) and *y*‐direction (*Q*_*y*,in_ [L^3^ T^−1^] and *Q*_*y*,out_ [L^3^ T^−1^]). Net flows across 3D model cells were then determined, given by ∆*Q*_*x*_ (L^3^ T^−1^) and ∆*Q*_*y*_ (L^3^ T^−1^) for the *x* and *y* directions, respectively (see Figure 4). Negative values of ∆*Q*_*x*_ and ∆*Q*_*y*_ indicate fluid loss from the cell, akin to extraction, while positive values signify fluid gain into the cell. For oblique cross‐sectional models, ∆*Q*_*x*,p_ (L^3^ T^−1^) and ∆*Q*_*y*,p_ (L^3^ T^−1^) are the components of ∆*Q*_*x*_ and ∆*Q*_*y*_ (respectively) that are perpendicular to the cross‐section axis, given by:

(4) $$\Delta Q_{x,p}=\Delta Q_{x}\sin\left(\alpha \right)$$

(5) $$\Delta Q_{y,p}=\Delta Q_{y}\cos\left(\alpha \right)$$

Here, *α* (−) denotes the angle between the cross‐section axis and the *x*‐axis, as shown in Figure 4. The projection of flow from the 3D model in the direction of the cross‐section axis is captured by the calculations of the 2D model, so these are omitted from Figure 4b. The net flows perpendicular to the cross section were obtained so that these could be incorporated as injection or extraction terms in the 2D model to account for lateral flow that occurs in the 3D model but are otherwise neglected in the 2D model.

### Statistical Measures of the Match between 2D and 3D Models

The heads of 2D and 3D models (along the alignment of the 2D cross section) were compared using the root‐mean‐square error (RMSE) and bias (Moriasi et al. 2007). Bias is the head from the 3D model minus the corresponding head of the 2D model. Values of RMSE and bias closer to 0 signify a higher degree of agreement. Positive and negative values of bias suggest that the head of the 2D model is, on average, lower and higher (respectively) than that of the 3D model. Where the 2D model represents the merger of two cells in the 3D model, Equation (2) was employed to compute the 3D model head for comparison to the 2D model.

## Results and Discussion

### Comparison of 2D and 3D Models

**Figure 5:**
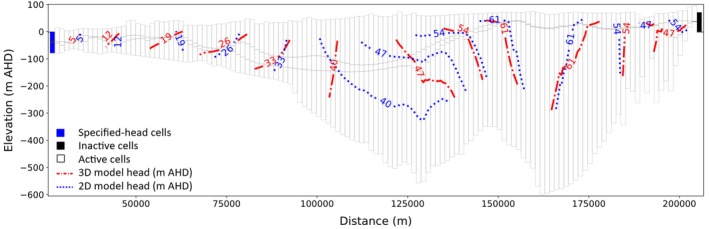
Head contours of 2D and 3D models along cross section EE′ (see Figure 1) at the end of the 16,081‐day simulation.

Figure 5 presents head contours for cross section EE′ at the end of the 16,081‐day (44 years) simulation, showing both the 2D and 3D model results. EE′ was chosen arbitrarily from the 10 cross sections shown in Figure 1, with corresponding outputs for other cross sections given in the Supporting Information (Figures S2–S10). This 2D model is oblique to the rectilinear grid of the 3D model, and it does not account for lateral flow in the 3D model. Pumping in the 3D model was transferred directly to the 2D model where pumping well cells were intercepted by the 2D model, rather than attempting to redistribute pumping (as described in section Aquifer Stresses [Pumping Wells, Recharge, Evapotranspiration, and Drains]).

The comparison of head contours in Figure 5 shows that the heads in this 2D model align with those of the 3D model in some areas, but the match is poor in others, most notably in the central part of the model. This is unsurprising given that cross section EE′ is not perpendicular to the head contours of the 3D model over most of its length (see Figure 1). The match between 2D and 3D head contours is better near specified‐head cells (i.e., the left boundary), as expected. RMSE and bias values for spatial error (i.e., reflecting differences in the head contours) in Figure 5 were 3.7 m and −1.8 m, respectively. The head matches of the other nine cross sections are provided in Figures S2–S10. These show that cross sections that do not intersect specified‐head cells (i.e., cross sections HH′ and II′) lead to head values that depart the most from those of the parent 3D model. Also, the improved match that arises around specified‐head cells in cross section EE′ is also apparent in other cross‐sectional models with specified heads.

**Figure 6:**
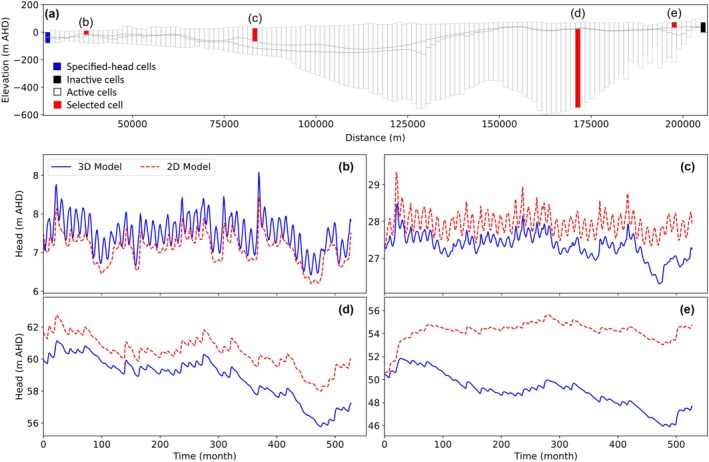
(a) Model grid of cross section EE′, showing the location of selected cells for which hydrographs from both the 2D and 3D models are shown in (b), (c), (d), and (e). Cells in (b), (c), and (e) are from the top layer of the model, whereas the cell in (d) is from the bottom layer.

The comparison between 3D and 2D models included time series of heads extracted from all active cells of each cross‐sectional model. Figure 6 presents a selection of these hydrographs for cross‐sectional model EE′, showing the comparison to those of the corresponding cells in the 3D model.

The comparison of hydrographs in Figure 6 indicates substantial disagreement between the 2D and 3D models that tended to worsen over time (e.g., Figure 6c to 6e). Figures S11–S19 of the Supporting Information present corresponding plots of a selection of hydrographs from the other nine cross sections. RMSE and bias values for the temporal error (i.e., comparison of hydrographs) apparent in Figure 6 ranged from 0.23 to 5.5 m and −5.2 to 0.19 m, respectively, for the four hydrographs. A more comprehensive statistical assessment of head errors is provided below.

**Figure 7:**
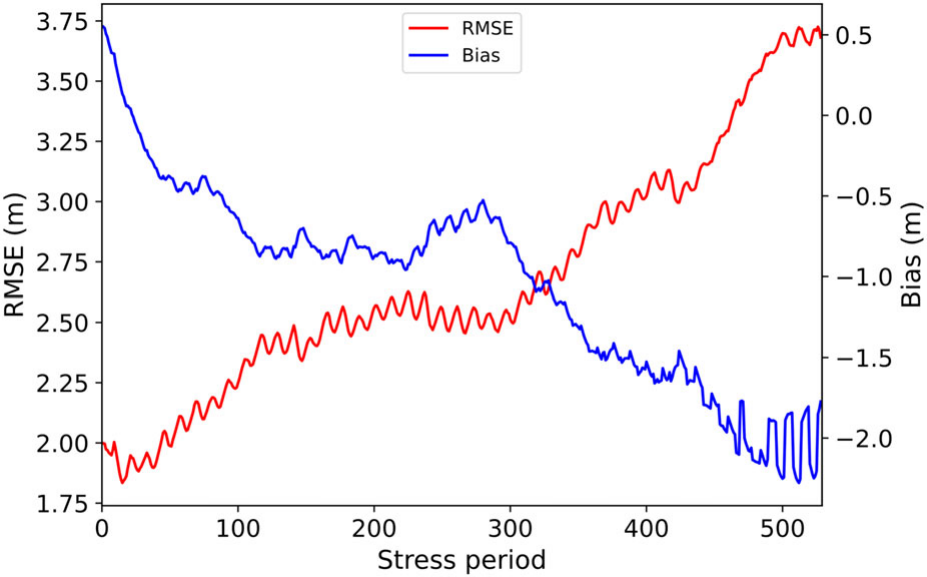
Temporal variation in the mean spatial error for cross section EE′.

**Figure 8:**
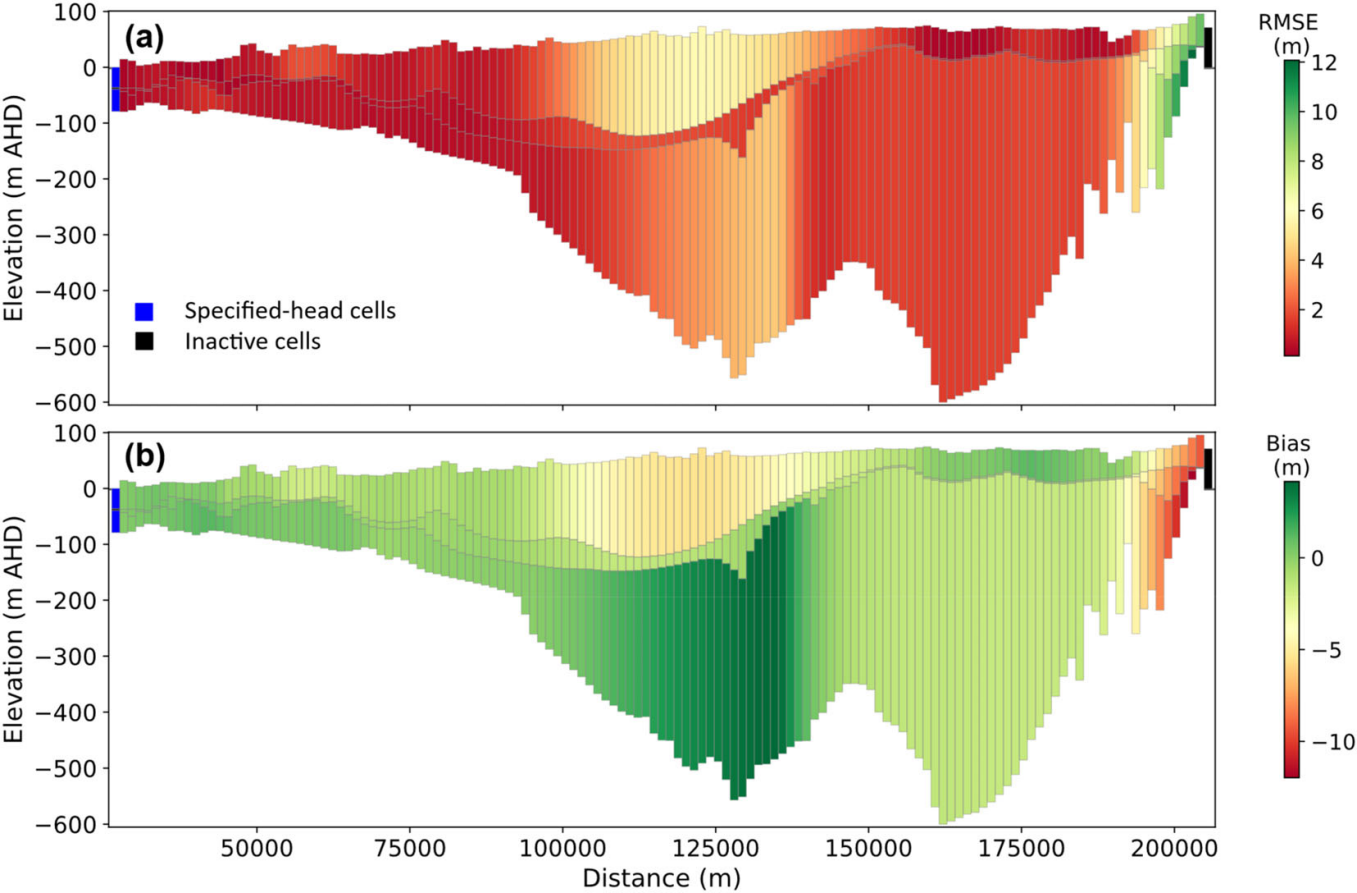
Spatial distribution of temporal error for cross section EE′: (a) RMSE, and (b) bias.

Statistical measures of the goodness‐of‐fit between 2D and 3D models were assessed for all cells at each time step (“spatial error”), and for all times at each cell (“temporal error”), thereby evaluating the spatial distribution and temporal evolution of head mismatch errors. Variability in the spatial error over time represents the transient evolution of errors, while spatial variability in the temporal error offers insight into the influence of model geometric features (e.g., boundary conditions, etc.) on the errors introduced by the extraction of the cross section from the 3D model. Figure 7 presents changes in the mean spatial error (RMSE and bias) over time, while Figure 8 shows the spatial distribution of temporal errors for cross section EE′.

In Figure 7, an overall upward trend (in time) in RMSE values is observed that reaches a peak of 3.7 m toward the end of the simulation. The bias has a positive value at the end of the first stress period, indicating that the cross‐sectional model underestimates the regional model heads at early times, eventually reaching about −1.8 m, representing an overestimation of the regional model heads. These results indicate, on average, a worsening match between 2D and 3D models with time. Changes in the spatial error with time for the remaining nine cross sections are presented in Figures S20–S28 (Supporting Information). There is a general upward trend (with time) in RMSE in these plots, consistent with Figure 7. The slope of the RMSE versus time relationship is steeper for cross sections that lack specified‐head cells. Mixed trends were obtained for the bias, some upward and some downward with time, for the other nine cross sections.

The results in Figure 8 demonstrate large spatial variability in the temporal error, with particularly large RMSE (up to 12 m) and bias (up to −12 m) near the right‐hand no‐flow boundary. Corresponding plots for the other nine cross sections are illustrated in Figures S29–S37 (Supporting Information). These confirm the observation that cross sections not intersecting specified‐head cells show poor alignment between 2D and 3D heads. Otherwise, causative factors for the trends observed in these plots are expected to include lateral flow and pumping effects (see sections Accounting for Lateral Flow in 2D Models and Aquifer Stresses [Pumping Wells, Recharge, Evapotranspiration, and Drains], respectively), while other issues (differences in numerical errors such as round‐off, etc.) cannot be discounted. The simple averaging method may also play a role in the mismatch between 2D and 3D models, especially for oblique cross sections.

Table S2 in the Supporting Information summarizes the spatial and temporal errors for each layer of all 10 cross‐sectional models. Spatial errors (RMSE and bias) tend to exhibit a narrower range compared to temporal errors, with the exception of cross sections HH′ and II′, which lack specified‐head cells. That is, errors tend to vary more significantly across space than they do over time. The bias of spatial errors is generally negative, indicating that 2D models are more likely to overestimate the groundwater heads of 3D models. The results in Table S2 show that cross sections FF′, EE′, and DD′ are best matched to the parent 3D model, at least based on RMSE and bias metrics. Nevertheless, errors are still unacceptable, with RMSE and bias as high as 12 and −12 m (respectively) for those cases. The worst error metrics were obtained for cross sections HH′ and II′ (RMSE and bias up to 27 and −27 m, respectively).

### Correcting for Lateral Flow

**Figure 9:**
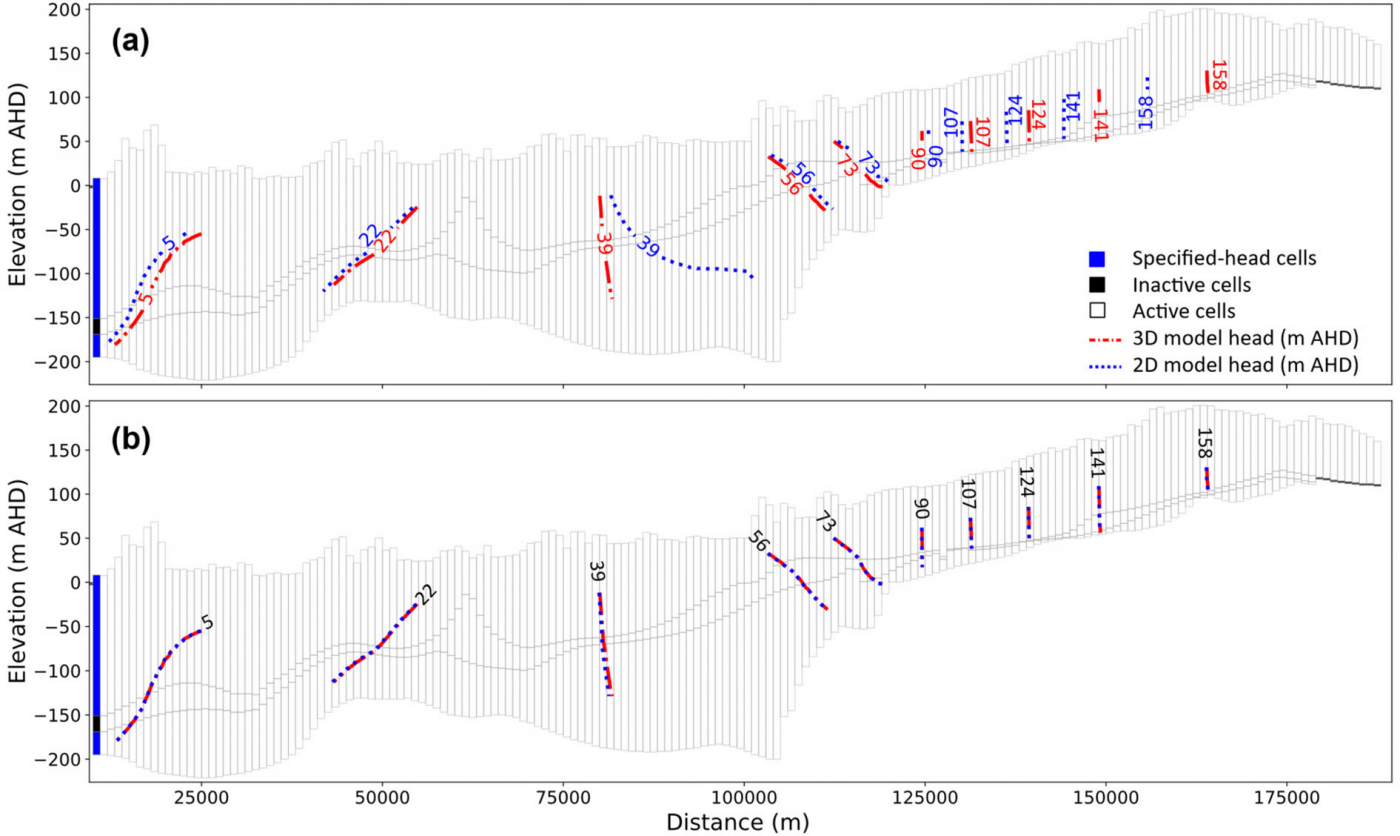
Head contours of 2D and 3D models along cross section JJ′ (at the end of the 16,081‐day simulation), showing results obtained: (a) without correcting for lateral flow, and (b) with lateral‐flow corrections applied.

The results presented above for 2D models highlight the need for corrections to be applied given the poor matches obtained. This was attempted through the incorporation of lateral flow, although only for cross section JJ′, which is parallel to the 3D model rectilinear grid. Attempts to account for lateral flow in oblique cross sections, requiring the amalgamation of partly intercepted 3D cells, were unsuccessful, and further research effort is needed to determine the lateral flow (in 3D models) that applies to oblique cross‐sectional models. Figure 9 presents the match in heads with and without corrections for lateral flow in cross section JJ′.

Figure 9a shows a similarly poor match to heads as was observed for cross section EE′ (Figure 5). The addition of a lateral flow correction, using the method outlined in section Accounting for Lateral Flow in 2D Models, produced head contours from the 2D model that closely matched those of the 3D model (Figure 9b). This is reflected in the lowering of the spatial RMSE and bias values at the end of the 16,081‐day simulation—from 7.6 to 0.022 m and from −4.8 to 0.014 m, respectively. When lateral flow is included, the temporal RMSE for all parts of the cross section is less than 0.031 m (Figure S38). A selection of hydrographs from cross section JJ′, with the lateral flow correction, is provided in Figure S39 (Supporting Information), while the temporal variability in spatial error with and without a correction for lateral flow is given in Figure S40. The results of Figures S39 and S40 confirm the strong match between the 2D and 3D models in different parts of the cross section with the addition of the lateral flow correction. This result not only demonstrates the applicability of the proposed lateral flow correction, but also validates the method for extracting parameters from the regional model for application in the 2D model.

An attempt was made to examine whether head errors and lateral flows added to the 2D model are correlated. Figure S41 presents the lateral flows added to the 2D model plotted with the error in heads in the 2D model (i.e., head differences between the 3D and 2D model) for four arbitrarily selected cells of cross section JJ′. A broader search for correlation was also undertaken, involving a wider gamut of the head and lateral flow results. It appears from Figure S41 (and a broader investigation) that there is no correlation between lateral flow and the head error of the 2D model. This is most likely due to the transient nature of both the head error and the lateral flow correction that has temporal dependencies of antecedent conditions and the stresses on the 3D system at locations beyond the cross‐sectional model domain.

### Redistribution of Pumping

**Figure 10:**
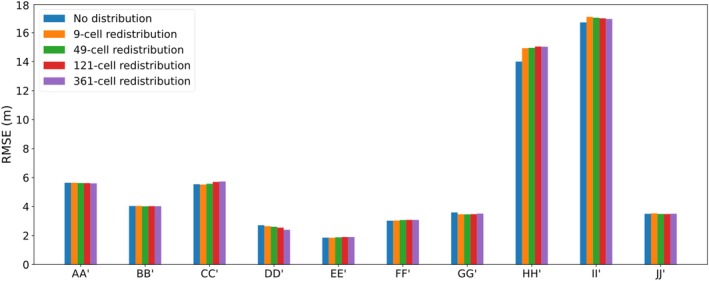
Comparison of RMSE arising from different pumping redistribution scenarios.

Figure 10 compares 2D and 3D models for various pumping redistribution scenarios using the average of the temporal RMSE for all 10 cross sections. Figure S42 (Supporting Information) provides the same comparison except in terms of bias. These results were calculated under conditions where lateral flow was not included in cross‐sectional models.

Figures 10 and S41 show that only minor differences (e.g., <10% change in RMSE) were obtained by redistributing pumping within the 3D model. In five cases, pumping redistribution led to a better overall match between 2D and 3D models, while in the other five cases, worse matches were obtained when pumping was redistributed. The minor changes observed in these results are likely due to the limited contribution of pumping to the overall water balance of the 3D model, accounting for only 4% of total outflows (Table S1).

Given that pumping is a larger component of the groundwater balance in many other situations (e.g., Knowling et al. 2015), pumping redistribution was assessed for a simplified case where pumping was a larger proportion of the overall water balance and wells were more sparsely situated than those of the regional case study. A simple model was constructed for this analysis consisting of 120 rows and 60 columns. Figure S43 depicts the map view of the model, showing the locations of cross sections and wells. The 3D model parameters are included in Table S3. The model water balance is summarized in Table S4 (Supporting Information), whereby pumping accounts for 99% of the total outflow.

Figure S44a illustrates the effects of 9‐cell and 25‐cell redistributions of pumping on the head match between the 2D and 3D models when the cross section intersects a pumping well. This comparison was also conducted for head values when the cross section is one cell away from the pumping well (Figure S44b). As expected, the drawdown was over‐represented in the 2D model when the pumping cell was intercepted (Figure S44a), while drawdown was under‐represented for the 2D model that was close to a pumping cell (Figure S44b). Pumping redistribution reduced drawdown in the former, while in the latter, drawdown from the nearby pumping cell was introduced into the 2D model that was otherwise neglected without pumping redistribution. The results in Figure S44 show that both the 9‐cell and 25‐cell redistributions enhance the alignment of head values between the 2D and 3D models. The head match of the 25‐cell redistribution was better than that of the 9‐cell redistribution. Whether or not pumping redistribution is required prior to the extraction of a 2D model from a 3D model appears to depend on the contribution of pumping to the water balance and the distribution of pumping cells, and should be evaluated based on the user's specific model setup. Certainly, significant errors were observed in the 2D models of the hypothetical case study without pumping redistribution.

## Conclusions

The current study assessed the agreement between 3D models and cross‐sectional models, both aligned and oblique to the 3D model's rectilinear grid, by comparing their heads. The results revealed major discrepancies between the 2D and 3D models, with the most severe errors (RMSE up to 27 m) observed in cross sections not intersecting with specified‐head cells. These discrepancies can be mitigated by incorporating lateral flows as inflows/outflows in 2D models, as demonstrated for a cross section that aligned with the 3D model's rectilinear grid. Attempts to find lateral‐flow corrections for 2D models that are oblique to the 3D model's grid were unsuccessful, highlighting the need for further research to develop a more generalizable methodology. The effect of redistributing pumping stresses on the match between 2D and 3D models demonstrated that redistribution enhances the alignment between the 2D and 3D models when there is a significant contribution of pumping to the 3D model's water balance. Otherwise, pumping redistribution may be ineffective.

Despite the challenges of cross‐sectional simulation, the proposed method has potential for various applications at the field scale, particularly in settings with fewer anthropogenic stresses. The ability to extract oblique cross sections from a 3D model offers a practical and efficient tool in cases where alignment with natural flow paths is important. Nevertheless, the techniques described in this study have wide‐ranging applications in constructing cross‐sectional models from 3D parent models, especially for the purpose of studying complex processes that require finer‐resolution grids, or as instructional tools for assessing generalized behavior rather than as site‐specific predictors of field‐scale conditions. Although cross‐sectional models are useful tools for studying practical groundwater problems, future efforts should emphasize approaches that reduce the computational demands of 3D groundwater models, such as solver optimization, localized refinement, or hybrid modeling frameworks, thereby allowing models to preserve spatial realism without the challenges that accompany cross‐sectional simulation.

## Authors' Note

The authors do not have any conflicts of interest or financial disclosures to report.

## Supporting information


**Data S1.** Supporting Information.

## Data Availability

The data that support the findings of this study are available on request from the corresponding author. The data are not publicly available due to privacy or ethical restrictions.
