# Supplementary material for: Cross‐Sectional Models of Groundwater Flow: Review and Correction for Transverse Flow
Source: Ground Water. 2025 Sep 3;63(5):752–63. doi: 10.1111/gwat.70017 (PMC12435104; doi:10.1111/gwat.70017)
Supplement: Supplementary file 1 — Data S1. Supporting Information. [file GWAT-63-752-s001.docx]

Supplementary Information for

**Cross-sectional models of groundwater flow: Review and correction for transverse flow**

Amin Gholami^1,2*^, Amir Jazayeri^1,2^, Adrian D. Werner^1,2^

^1^College of Science and Engineering, Flinders University, GPO Box 2100, Adelaide, SA 5001, Australia

^2^National Centre for Groundwater Research and Training, Flinders University, GPO Box 2100, Adelaide, SA 5001, Australia

*Corresponding author

Email addresses:

Amin Gholami: [amin.gholami@flinders.edu.au](mailto:amin.gholami@flinders.edu.au); [amingholamiput@yahoo.com](mailto:amingholamiput@yahoo.com)

*Submitted to: Groundwater on 28* *January 2025*

**Table S1.** The contributions of each component to the inflow and outflow of the 3D model of the Limestone Coast (Australia).

| Components | Percentage in inflow (%) | Percentage in outflow (%) |
| --- | --- | --- |
| Storage | 25 | 21 |
| Specified head | 0.18 | 34 |
| Wells | 0.0 | 4.0 |
| Drains | 0.0 | 5.6 |
| Evapotranspiration | 0.0 | 34 |
| Recharge | 74 | 0.0 |

**Table S2.** Summary of spatial and temporal errors in the results for cross sections depicted in Figure 1 of the Main Manuscript. Here, “Min” is the lowest temporal error value across active cells in the cross section or is the lowest spatial error from all time steps in the simulation. “Max” and “Mean” are the corresponding maximum and minimum values.

| Cross section | Layer | Description | RMSE (m) | | | Bias (m) | | |
| --- | --- | --- | --- | --- | --- | --- | --- | --- |
|  |  |  | Min | Max | Mean | Min | Max | Mean |
| AA′ | 1 | Temporal error | 0.16 | 13 | 3.2 | ‒12 | 2.5 | ‒2.8 |
|  |  | Spatial error | 0.83 | 6.3 | 3.8 | ‒4.6 | ‒0.2 | ‒2.8 |
|  | 2 | Temporal error | 0.56 | 13 | 5.8 | ‒12 | ‒0.51 | ‒5.7 |
|  |  | Spatial error | 3.2 | 8.7 | 6.4 | ‒7.6 | ‒2.7 | ‒5.7 |
|  | 3 | Temporal error | 0.62 | 14 | 8.3 | ‒14 | ‒0.56 | ‒8.3 |
|  |  | Spatial error | 6.3 | 11 | 9.3 | ‒10 | ‒5.5 | ‒8.3 |
| BB′ | 1 | Temporal error | 0.32 | 11 | 6.3 | ‒11 | 0.11 | ‒6.1 |
|  |  | Spatial error | 0.25 | 8.5 | 7.0 | ‒7.4 | ‒0.13 | ‒6.1 |
|  | 2 | Temporal error | 0.12 | 6.9 | 3.9 | ‒6.6 | ‒0.059 | ‒3.6 |
|  |  | Spatial error | 0.84 | 5.3 | 4.2 | ‒4.7 | 1.4 | ‒3.6 |
|  | 3 | Temporal error | 0.15 | 3.7 | 2.2 | ‒3.6 | 2.2 | ‒1.1 |
|  |  | Spatial error | 1.4 | 3.8 | 2.4 | ‒2.0 | 3.3 | ‒1.1 |
| CC′ | 1 | Temporal error | 0.091 | 16 | 4.5 | ‒0.63 | 15 | 4.0 |
|  |  | Spatial error | 0.10 | 9.1 | 5.8 | 0.041 | 6.2 | 4.0 |
|  | 2 | Temporal error | 0.12 | 19 | 5.5 | ‒0.27 | 18 | 5.1 |
|  |  | Spatial error | 1.8 | 10 | 7.0 | 1.3 | 7.2 | 5.1 |
|  | 3 | Temporal error | 0.070 | 22 | 6.7 | ‒0.69 | 21 | 6.4 |
|  |  | Spatial error | 3.6 | 11 | 8.4 | 2.6 | 8.3 | 6.4 |
| DD′ | 1 | Temporal error | 0.036 | 5.1 | 2.0 | ‒4.6 | 0.53 | ‒1.6 |
|  |  | Spatial error | 0.054 | 4.4 | 2.5 | ‒3.1 | ‒0.0081 | ‒1.6 |
|  | 2 | Temporal error | 0.074 | 4.7 | 2.7 | ‒4.3 | 3.6 | ‒1.4 |
|  |  | Spatial error | 1.6 | 4.2 | 2.8 | ‒3.0 | 0.21 | ‒1.4 |
|  | 3 | Temporal error | 0.13 | 7.4 | 3.6 | ‒5.7 | 7.3 | ‒1.2 |
|  |  | Spatial error | 3.2 | 4.7 | 3.8 | ‒3.0 | 0.53 | ‒1.2 |
| EE′ | 1 | Temporal error | 0.13 | 9.6 | 2.3 | ‒9.3 | 1.2 | ‒1.9 |
|  |  | Spatial error | 0.12 | 4.7 | 3.0 | ‒3.2 | ‒0.029 | ‒1.9 |
|  | 2 | Temporal error | 0.23 | 10 | 1.4 | ‒10 | 0.79 | ‒1.0 |
|  |  | Spatial error | 1.2 | 3.2 | 2.1 | ‒2.3 | 0.55 | ‒1.0 |
|  | 3 | Temporal error | 0.23 | 12 | 1.9 | ‒12 | 4.2 | ‒0.24 |
|  |  | Spatial error | 2.5 | 3.3 | 2.8 | ‒1.4 | 1.1 | ‒0.24 |
| FF′ | 1 | Temporal error | 0.066 | 9.4 | 2.6 | ‒8.6 | 0.88 | ‒2.1 |
|  |  | Spatial error | 0.11 | 5.6 | 3.2 | ‒4.1 | 0.013 | ‒2.1 |
|  | 2 | Temporal error | 0.19 | 10 | 2.9 | ‒9.8 | 1.3 | ‒2.2 |
|  |  | Spatial error | 1.1 | 5.6 | 3.5 | ‒4.2 | ‒0.03 | ‒2.2 |
|  | 3 | Temporal error | 0.52 | 12 | 3.6 | ‒11 | 2.7 | ‒2.2 |
|  |  | Spatial error | 2.3 | 5.9 | 4.2 | ‒4.3 | ‒0.019 | ‒2.2 |
| GG′ | 1 | Temporal error | 0.056 | 7.1 | 2.6 | 0.042 | 6.4 | 2.3 |
|  |  | Spatial error | 0.071 | 5 | 3.1 | 0.039 | 3.6 | 2.3 |
|  | 2 | Temporal error | 0.24 | 7.7 | 3.6 | 0.24 | 7.2 | 3.4 |
|  |  | Spatial error | 1.6 | 5.5 | 3.9 | 1.2 | 4.6 | 3.4 |
|  | 3 | Temporal error | 0.44 | 8.4 | 4.7 | 0.44 | 7.9 | 4.5 |
|  |  | Spatial error | 3.1 | 6.3 | 4.7 | 2.4 | 5.7 | 4.5 |
| HH′ | 1 | Temporal error | 3.9 | 22 | 13 | ‒20 | ‒2.2 | ‒12 |
|  |  | Spatial error | 0.14 | 21 | 13 | ‒21 | ‒0.053 | ‒12 |
|  | 2 | Temporal error | 5.0 | 24 | 14 | ‒23 | ‒3.8 | ‒13 |
|  |  | Spatial error | 1.1 | 22 | 13 | ‒21 | ‒0.74 | ‒13 |
|  | 3 | Temporal error | 5.2 | 26 | 15 | ‒25 | ‒4 | ‒13 |
|  |  | Spatial error | 2.2 | 23 | 14 | ‒22 | ‒1.4 | ‒13 |
| II′ | 1 | Temporal error | 8.9 | 22 | 16 | ‒20 | ‒7.6 | ‒14 |
|  |  | Spatial error | 0.20 | 26 | 15 | ‒26 | ‒0.061 | ‒14 |
|  | 2 | Temporal error | 9.0 | 24 | 17 | ‒22 | ‒7.7 | ‒15 |
|  |  | Spatial error | 1.1 | 27 | 15 | ‒27 | ‒0.66 | ‒15 |
|  | 3 | Temporal error | 8.3 | 25 | 17 | ‒24 | ‒6.9 | ‒16 |
|  |  | Spatial error | 2.1 | 27 | 16 | ‒27 | ‒1.3 | ‒16 |
| JJ′ | 1 | Temporal error | 0.11 | 12 | 3.1 | ‒11 | 0.49 | ‒2.7 |
|  |  | Spatial error | 0.068 | 7.3 | 4.5 | ‒4.7 | ‒0.027 | ‒2.7 |
|  | 2 | Temporal error | 0.29 | 12 | 3.5 | ‒11 | 1.9 | ‒2.6 |
|  |  | Spatial error | 0.89 | 7.6 | 4.9 | ‒4.8 | 0.21 | ‒2.6 |
|  | 3 | Temporal error | 0.45 | 13 | 4.0 | ‒12 | 4.0 | ‒2.4 |
|  |  | Spatial error | 1.8 | 7.8 | 5.3 | ‒5.0 | 0.44 | ‒2.4 |

**Table S3.** Summary of parameters for the 3D model simulation setup.

| Package | Parameters | Unit | Specifications |
| --- | --- | --- | --- |
| Discretization | Number of periods | - | 5 |
|  | Number of time steps | - | 100 |
|  | Length of each stress period | d | 5 |
|  | Number of layers | - | 1 |
|  | Number of columns | - | 60 |
|  | Number of rows | - | 120 |
|  | Column width | m | 10 |
|  | Row width | m | 10 |
|  | Top of the model | m | 40 |
|  | Bottom of the model | m | 0 |
| Basic | Initial head | m | 35 |
| Upstream flow | *K*_h_ | md^‒1^ | 20 |
|  | *K*_v_ | md^‒1^ | 10 |
|  | *S*_s_ | m^‒1^ | 1e-6 |
|  | *S*_y_ | - | 0.10 |
|  | Cell conversion type | - | Convertible |
| Recharge | Recharge rate | md^‒1^ | 0.0020 |

**Table S4.** The contributions of each component to the inflow and outflow of the simple 3D model.

| Components | Percentage in inflow (%) | Percentage in outflow (%) |
| --- | --- | --- |
| Storage | 91 | 0.33 |
| Wells | 0.0 | 99 |
| Recharge | 9.1 | 0.0 |


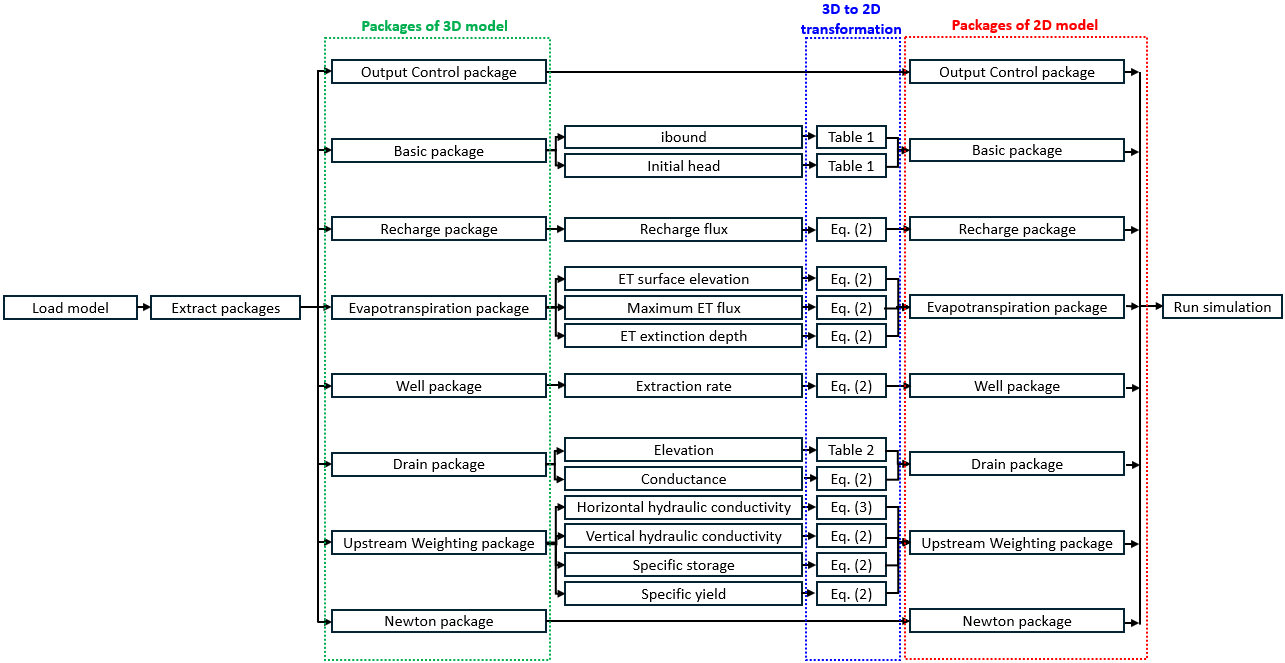


**Figure S1.** Workflow illustrating the step-by-step process of converting a 3D groundwater model into a 2D cross-sectional model.


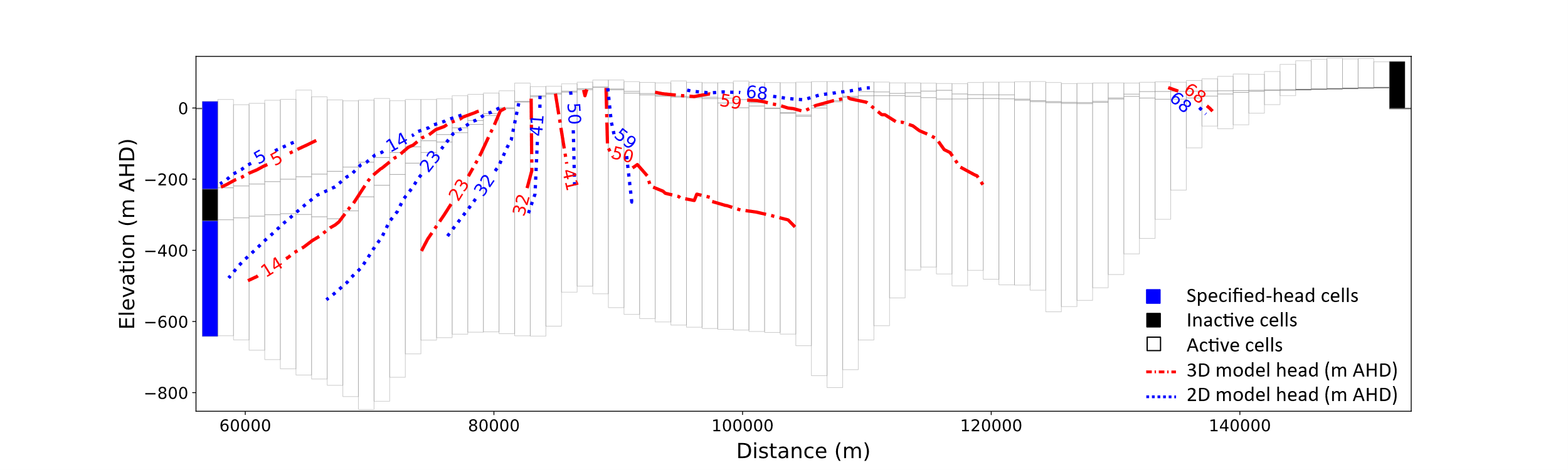


**Figure S2.** Head contours of 2D and 3D models along cross section AA′ at the end of the 16,081-day simulation.


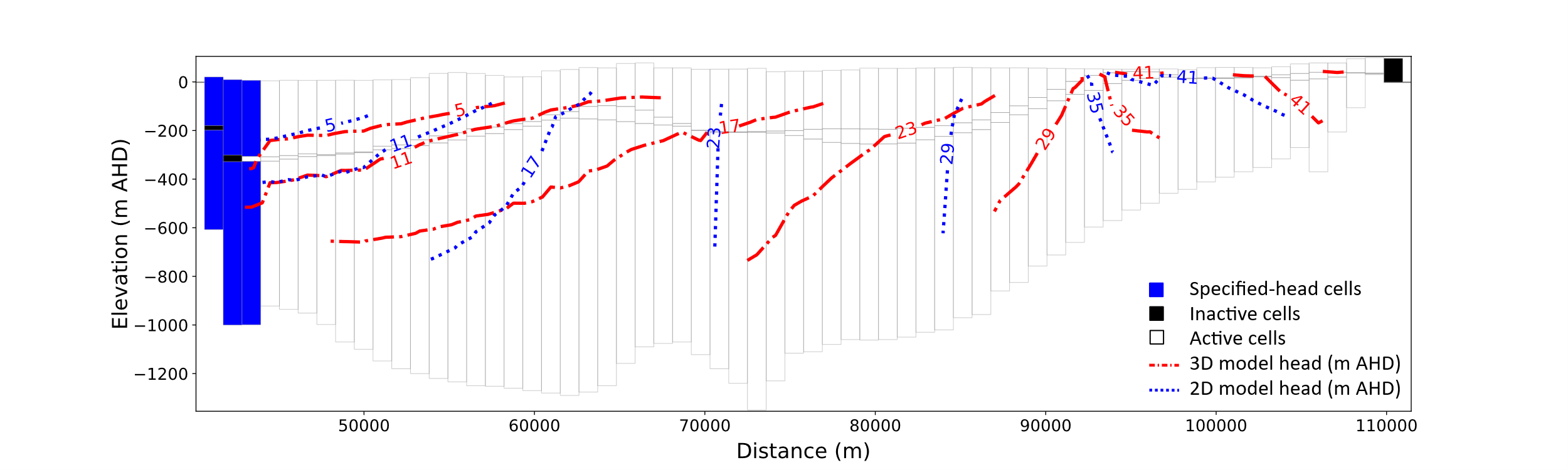


**Figure S3.** Head contours of 2D and 3D models along cross section BB′ at the end of the 16,081-day simulation.


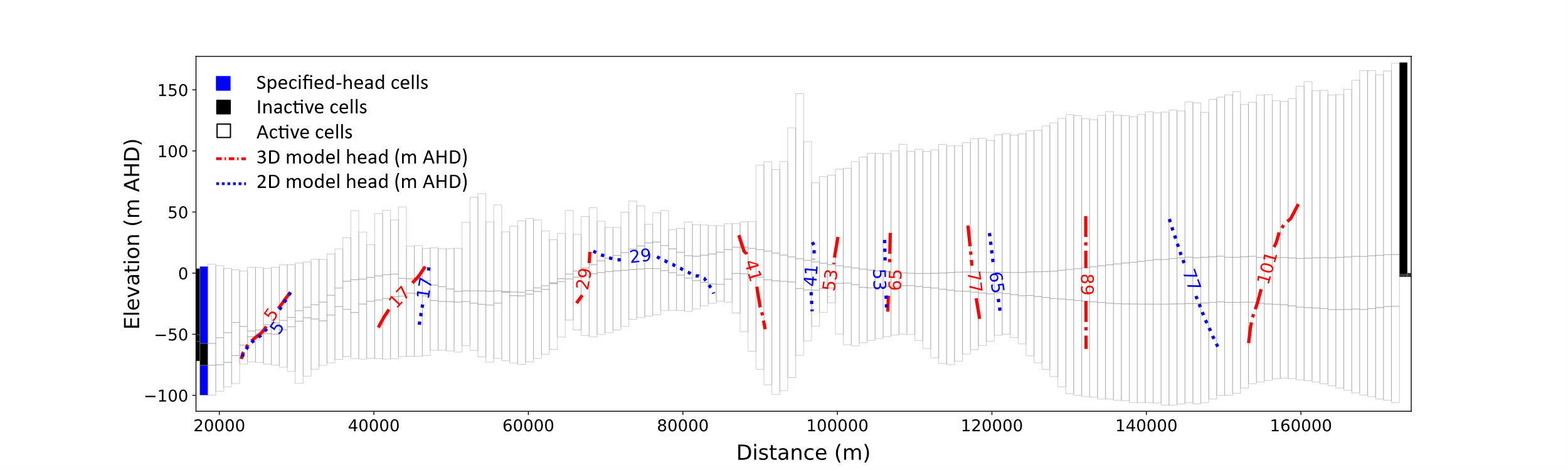


**Figure S4.** Head contours of 2D and 3D models along cross section CC′ at the end of the 16,081-day simulation.


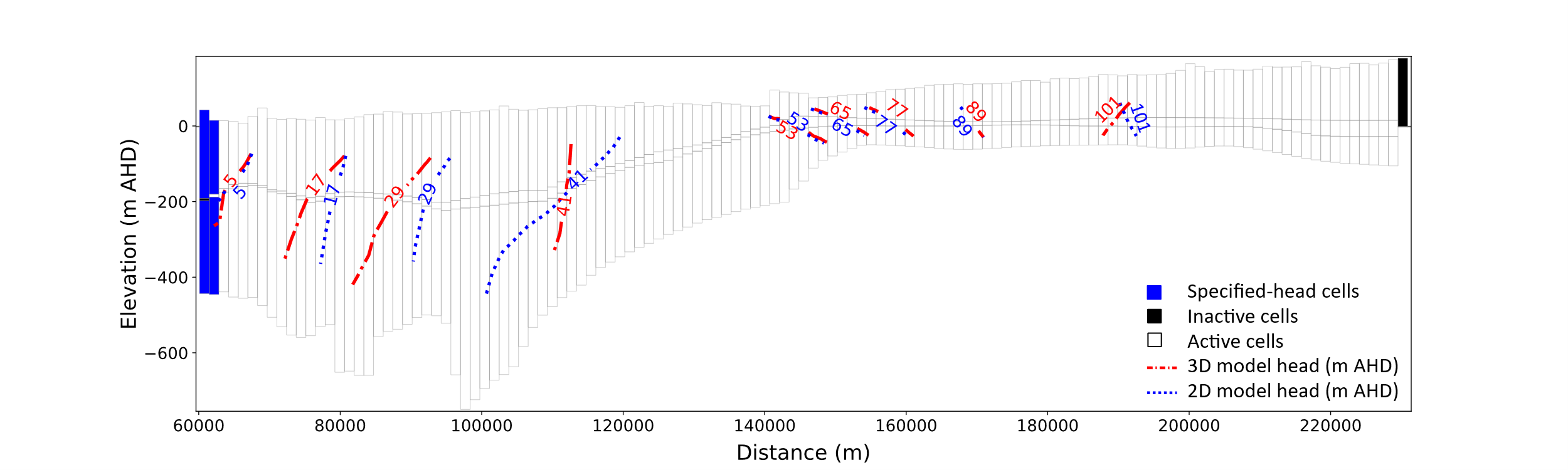


**Figure S5.** Head contours of 2D and 3D models along cross section DD′ at the end of the 16,081-day simulation.


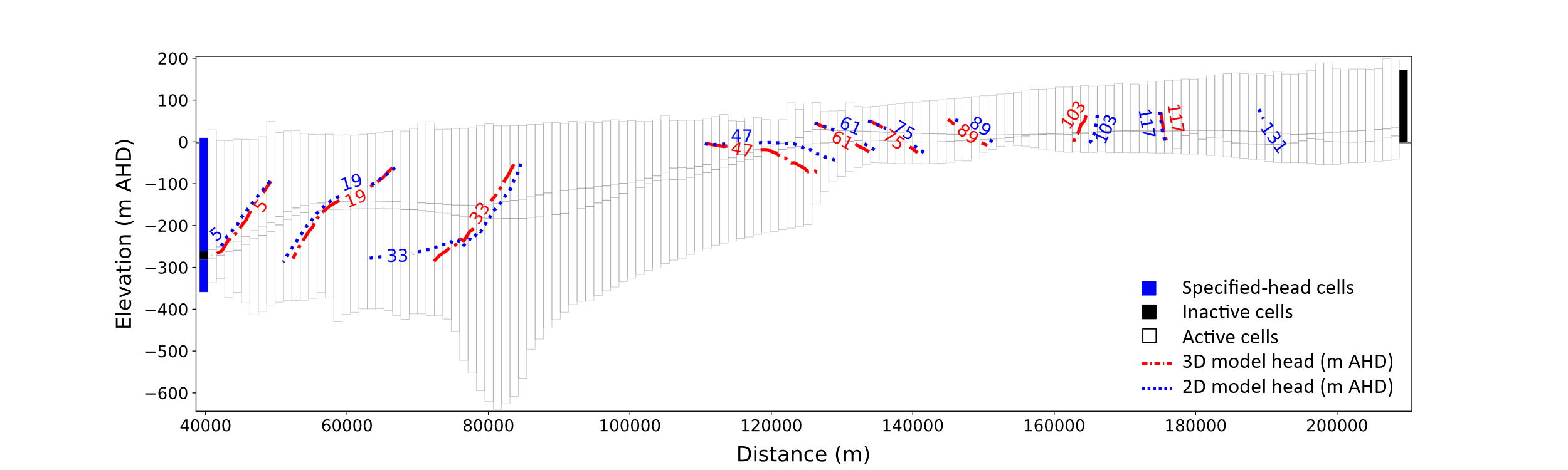


**Figure S6.** Head contours of 2D and 3D models along cross section FF′ at the end of the 16,081-day simulation.


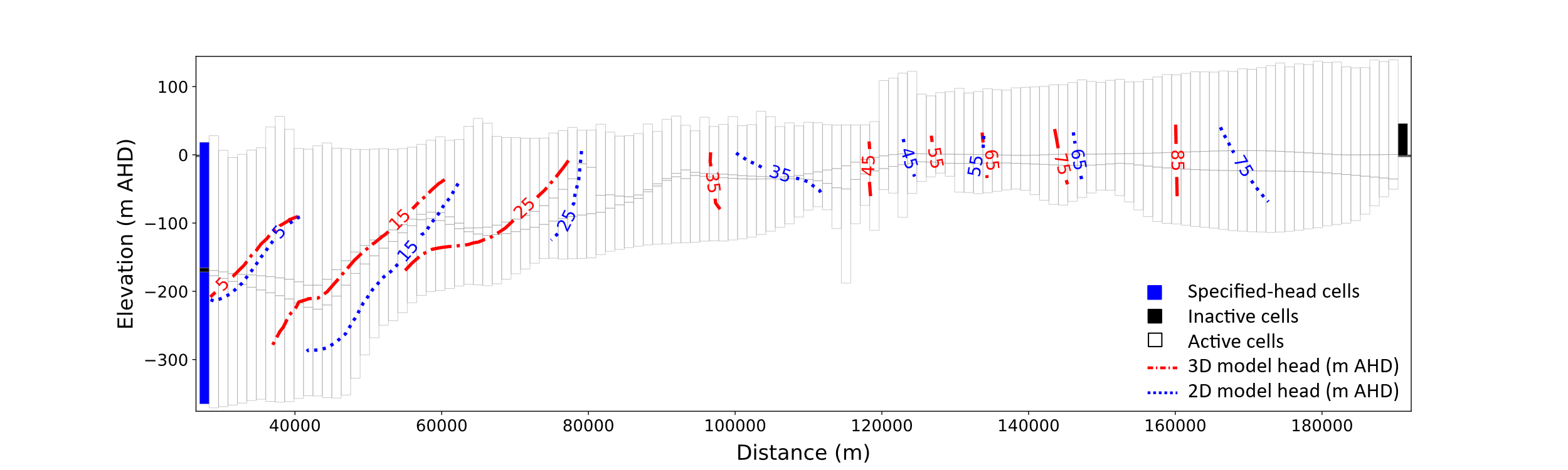


**Figure S7.** Head contours of 2D and 3D models along cross section GG′ at the end of the 16,081-day simulation.


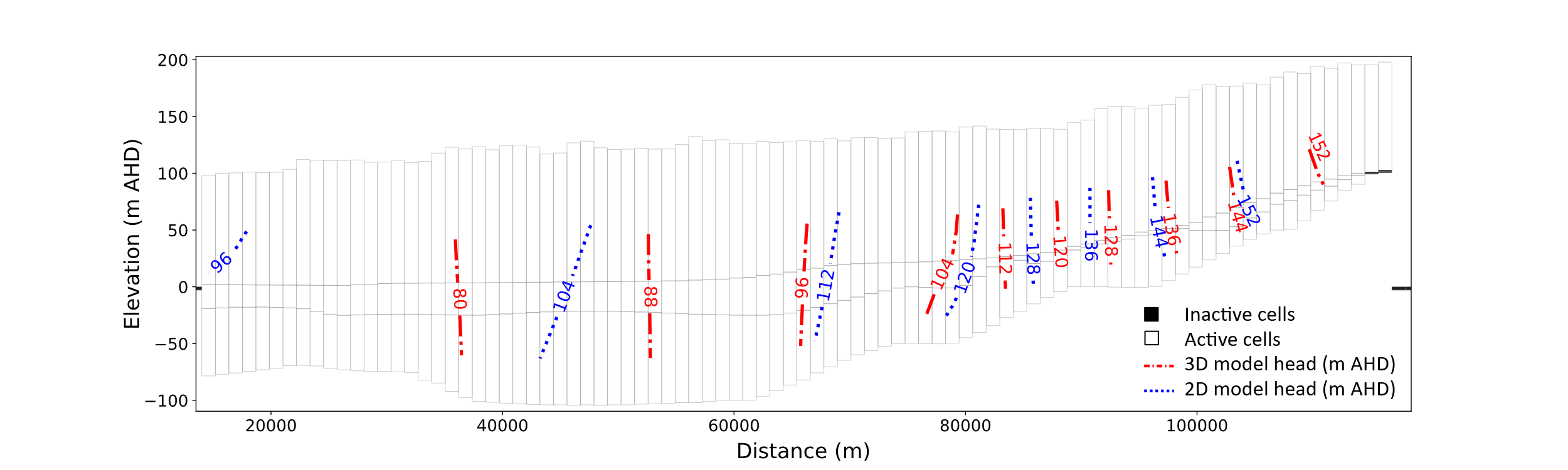


**Figure S8.** Head contours of 2D and 3D models along cross section HH′ at the end of the 16,081-day simulation.


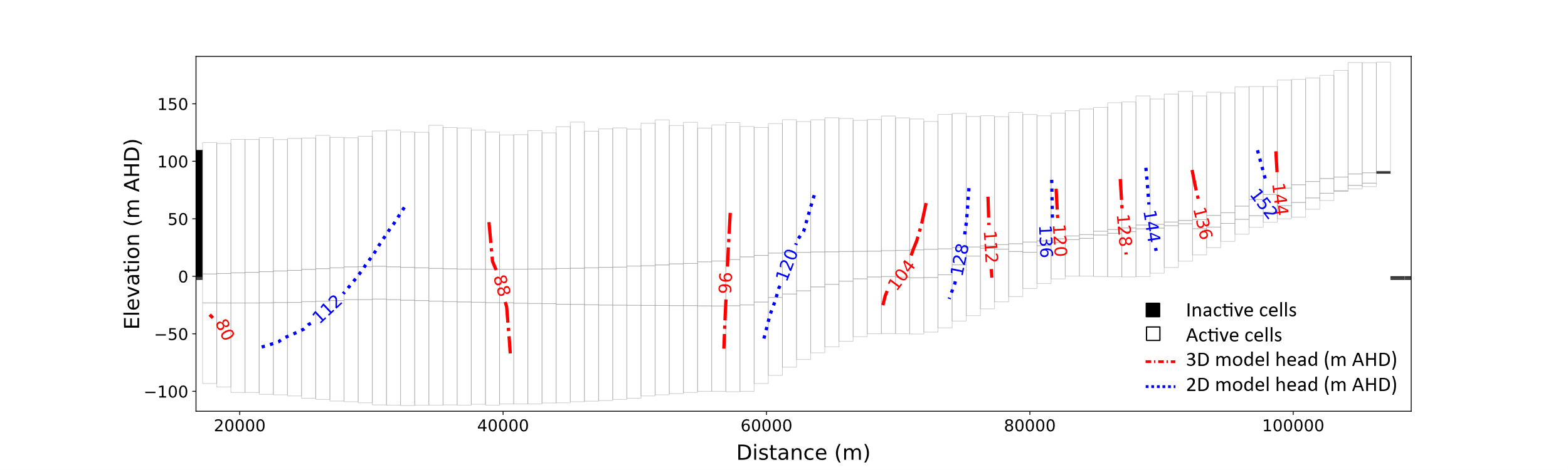


**Figure S9.** Head contours of 2D and 3D models along cross section II′ at the end of the 16,081-day simulation.


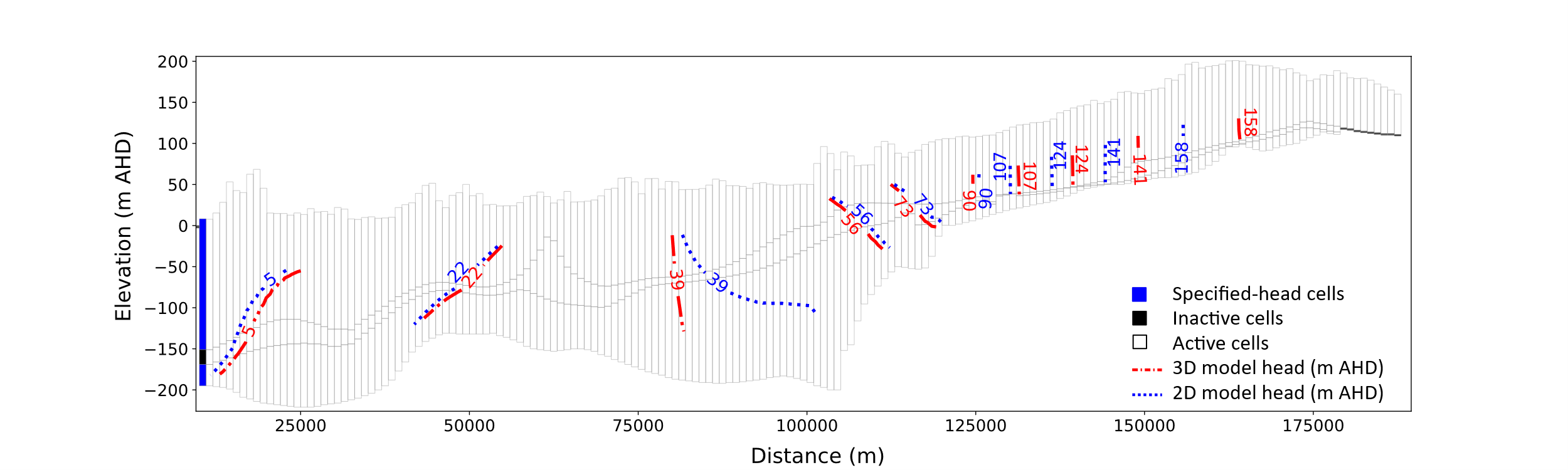


**Figure S10.** Head contours of 2D and 3D models along cross section JJ′ at the end of the 16,081-day simulation.

**
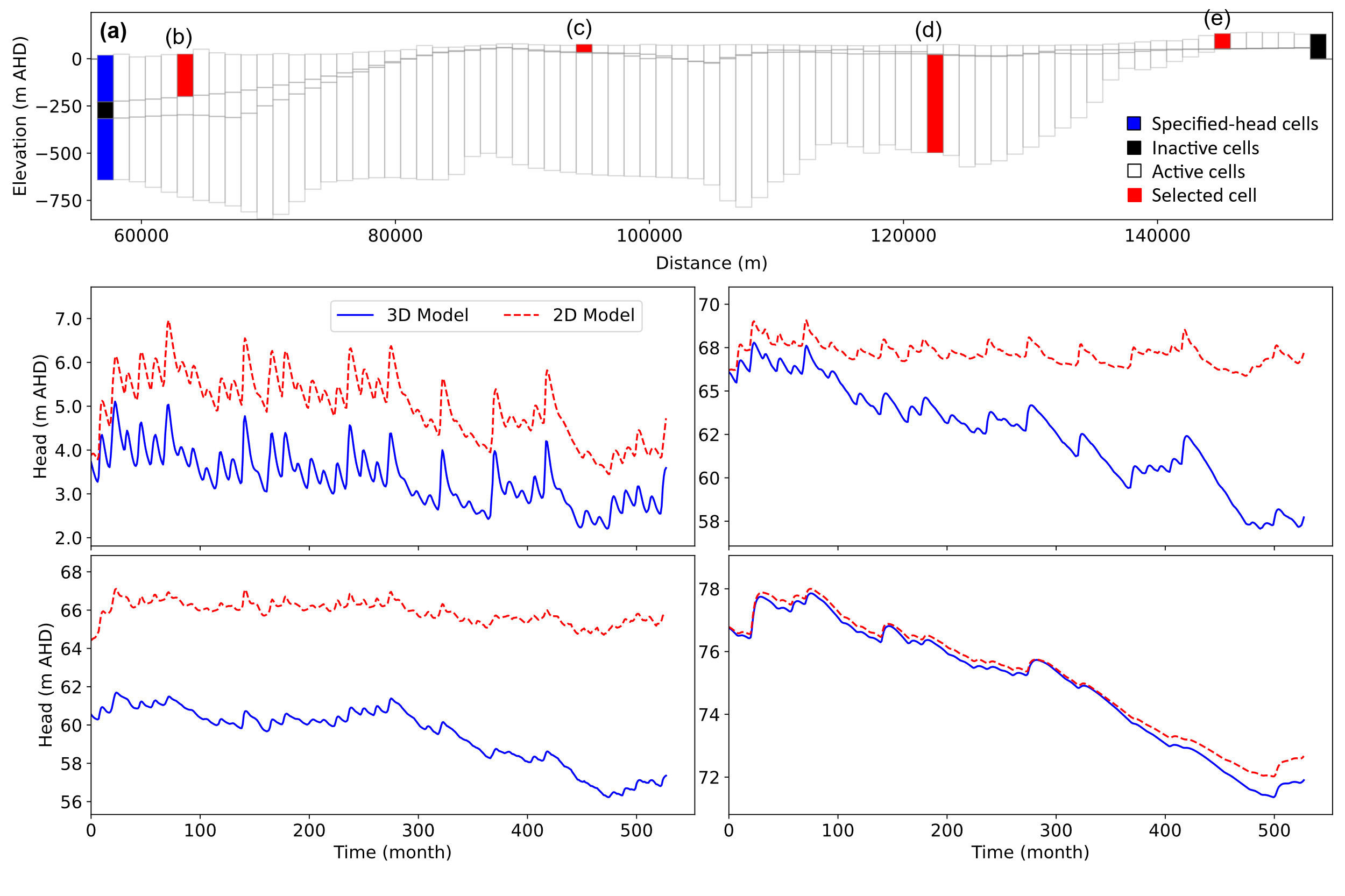
**

**Figure S11.** (a) Model grid of cross section AA′, showing the location of selected cells for which hydrographs from both the 2D and 3D models are shown in (b), (c), (d) and (e).


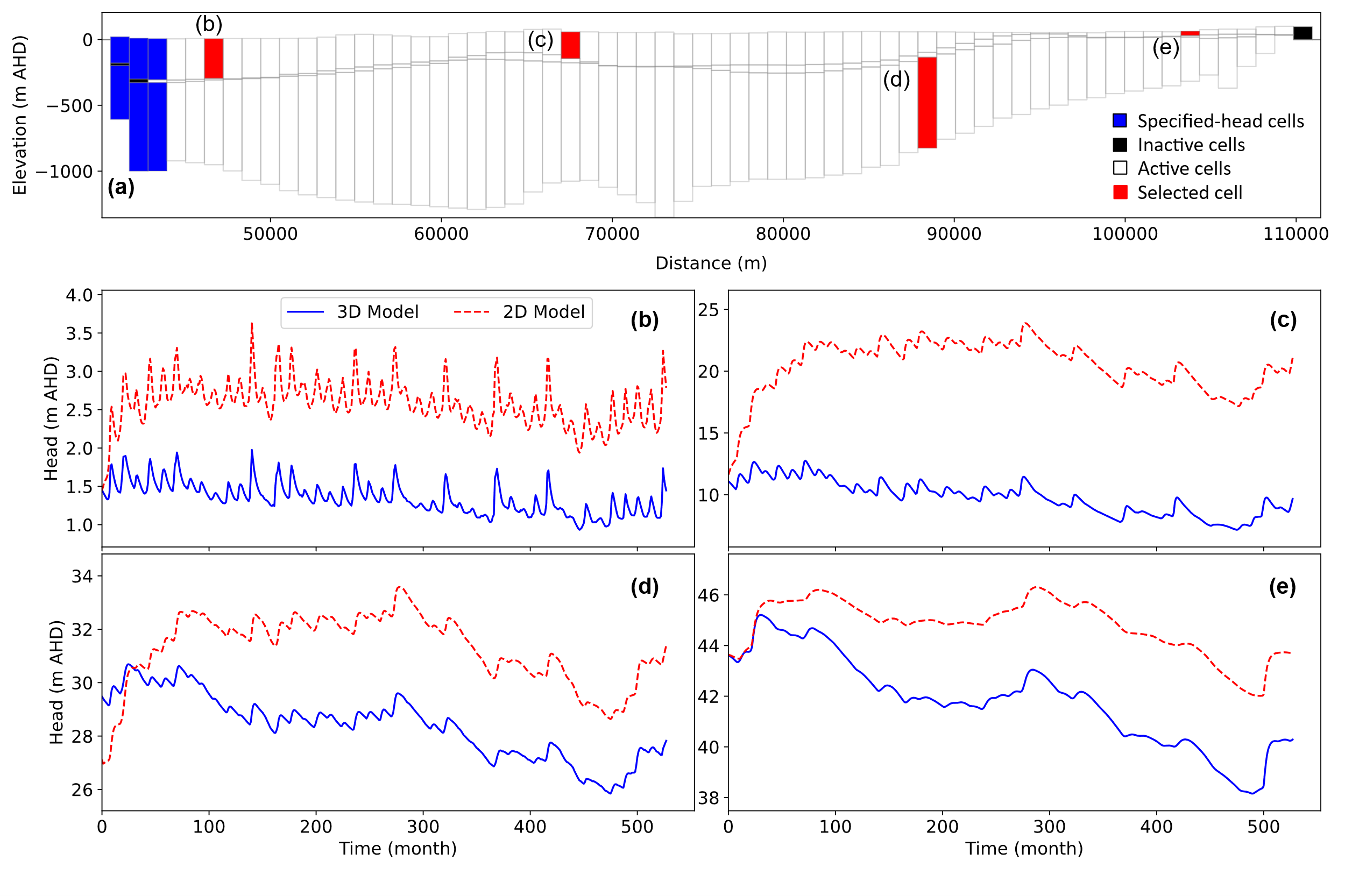


**Figure S12.** (a) Model grid of cross section BB′, showing the location of selected cells for which hydrographs from both the 2D and 3D models are shown in (b), (c), (d) and (e).


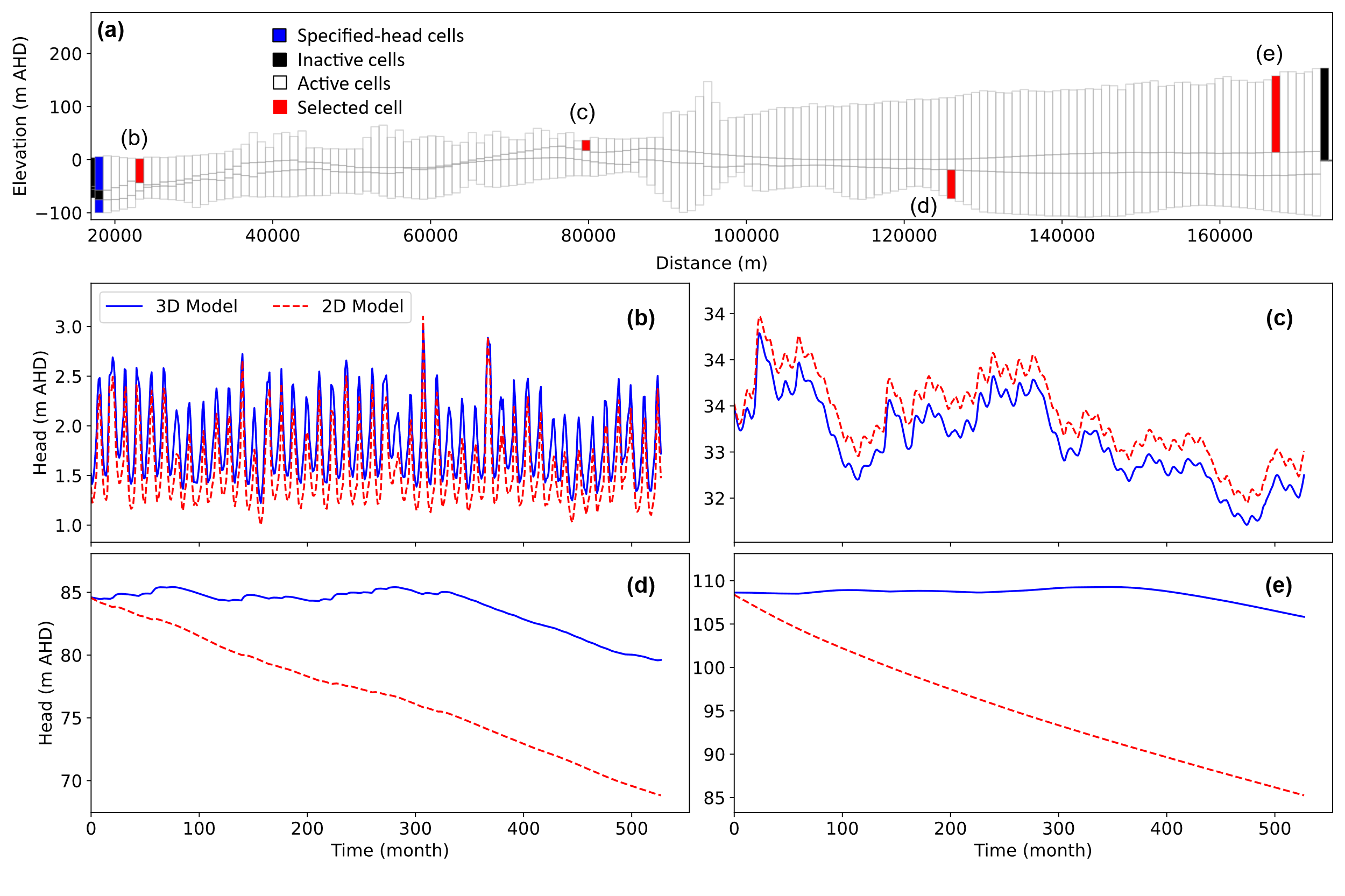


**Figure S13.** (a) Model grid of cross section CC′, showing the location of selected cells for which hydrographs from both the 2D and 3D models are shown in (b), (c), (d) and (e).


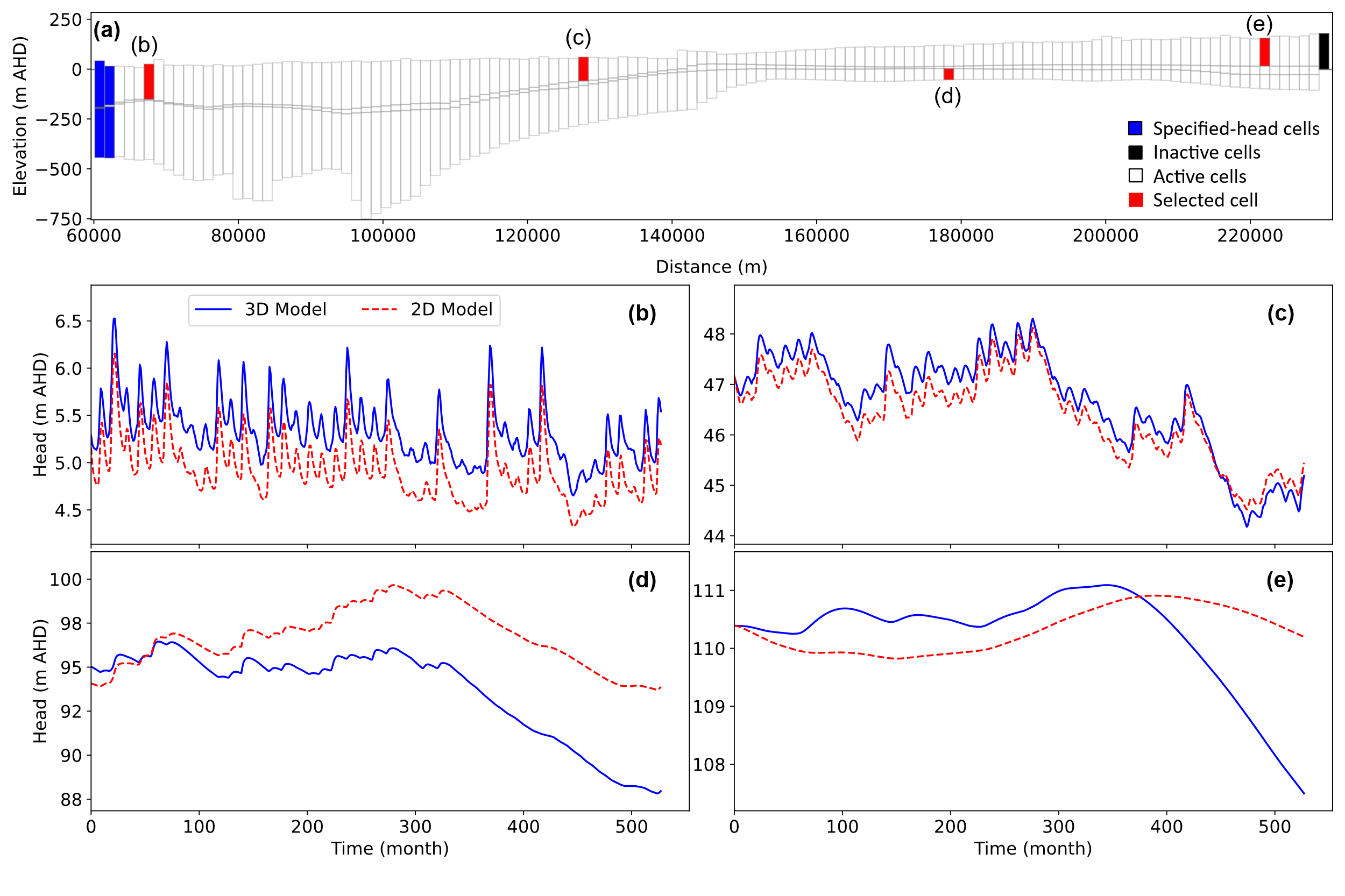


**Figure S14.** (a) Model grid of cross section DD′, showing the location of selected cells for which hydrographs from both the 2D and 3D models are shown in (b), (c), (d) and (e).


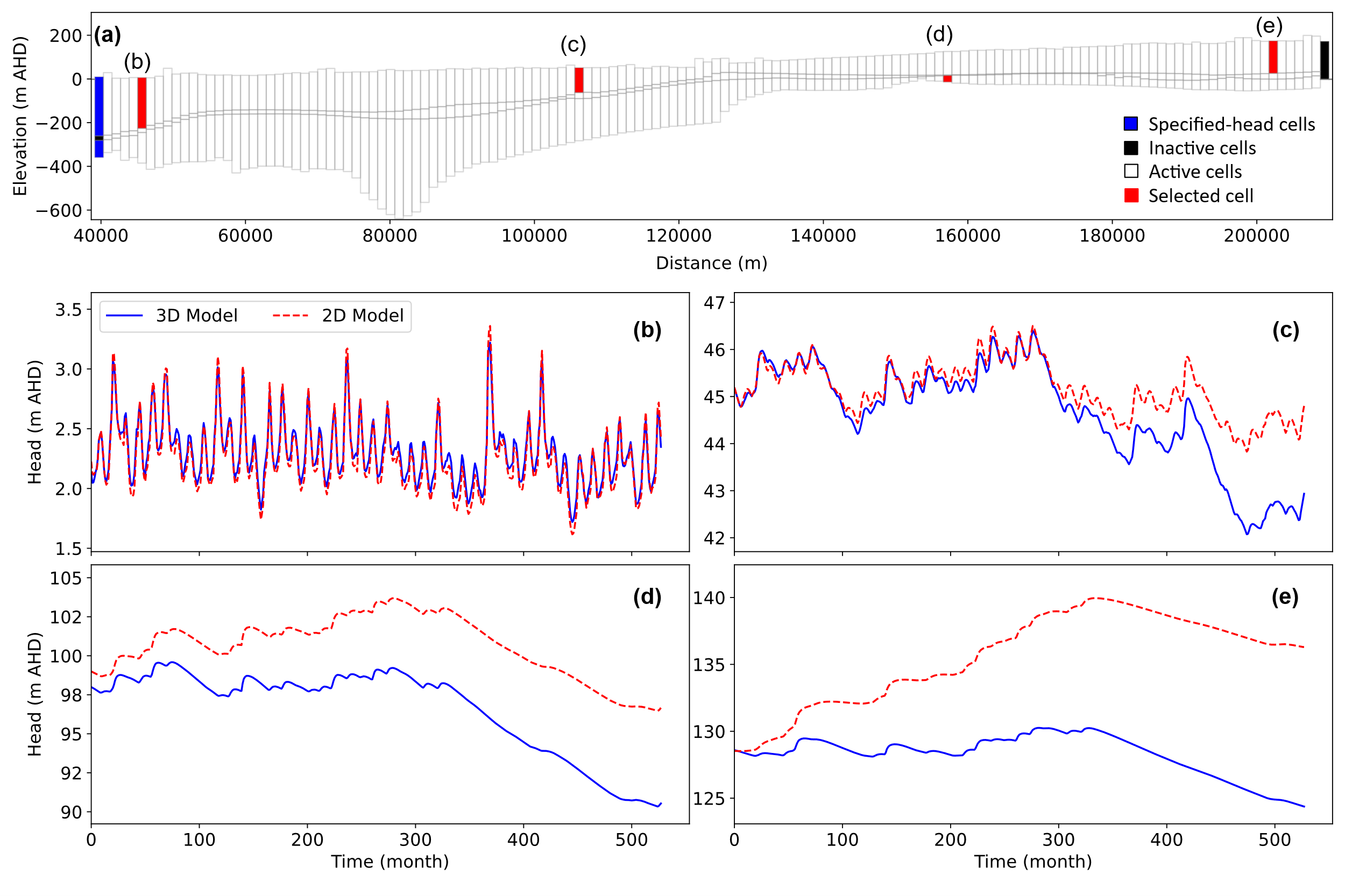


**Figure S15.** (a) Model grid of cross section FF′, showing the location of selected cells for which hydrographs from both the 2D and 3D models are shown in (b), (c), (d) and (e).


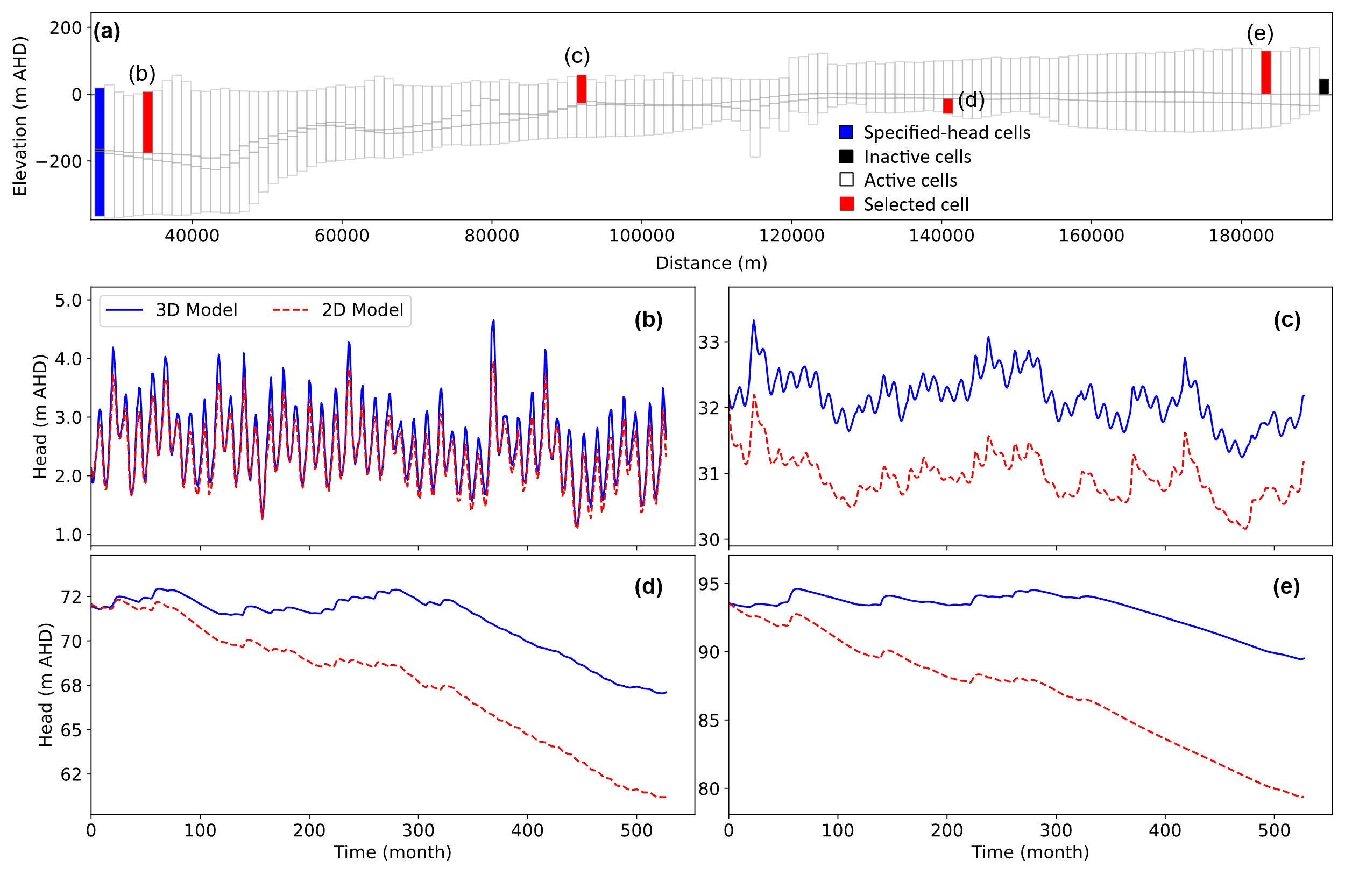


**Figure S16.** (a) Model grid of cross section GG′, showing the location of selected cells for which hydrographs from both the 2D and 3D models are shown in (b), (c), (d) and (e).


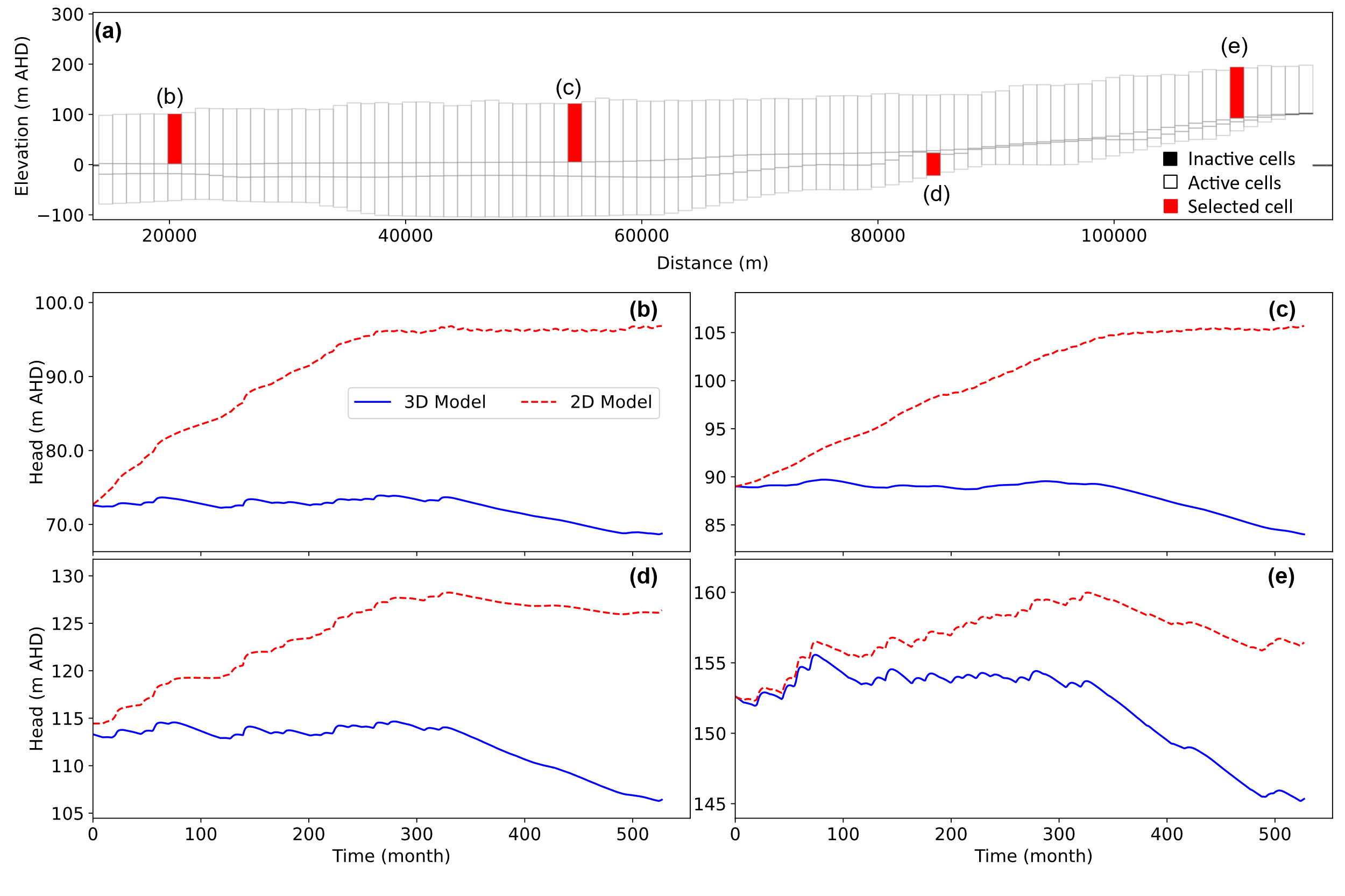


**Figure S17.** (a) Model grid of cross section HH′, showing the location of selected cells for which hydrographs from both the 2D and 3D models are shown in (b), (c), (d) and (e).


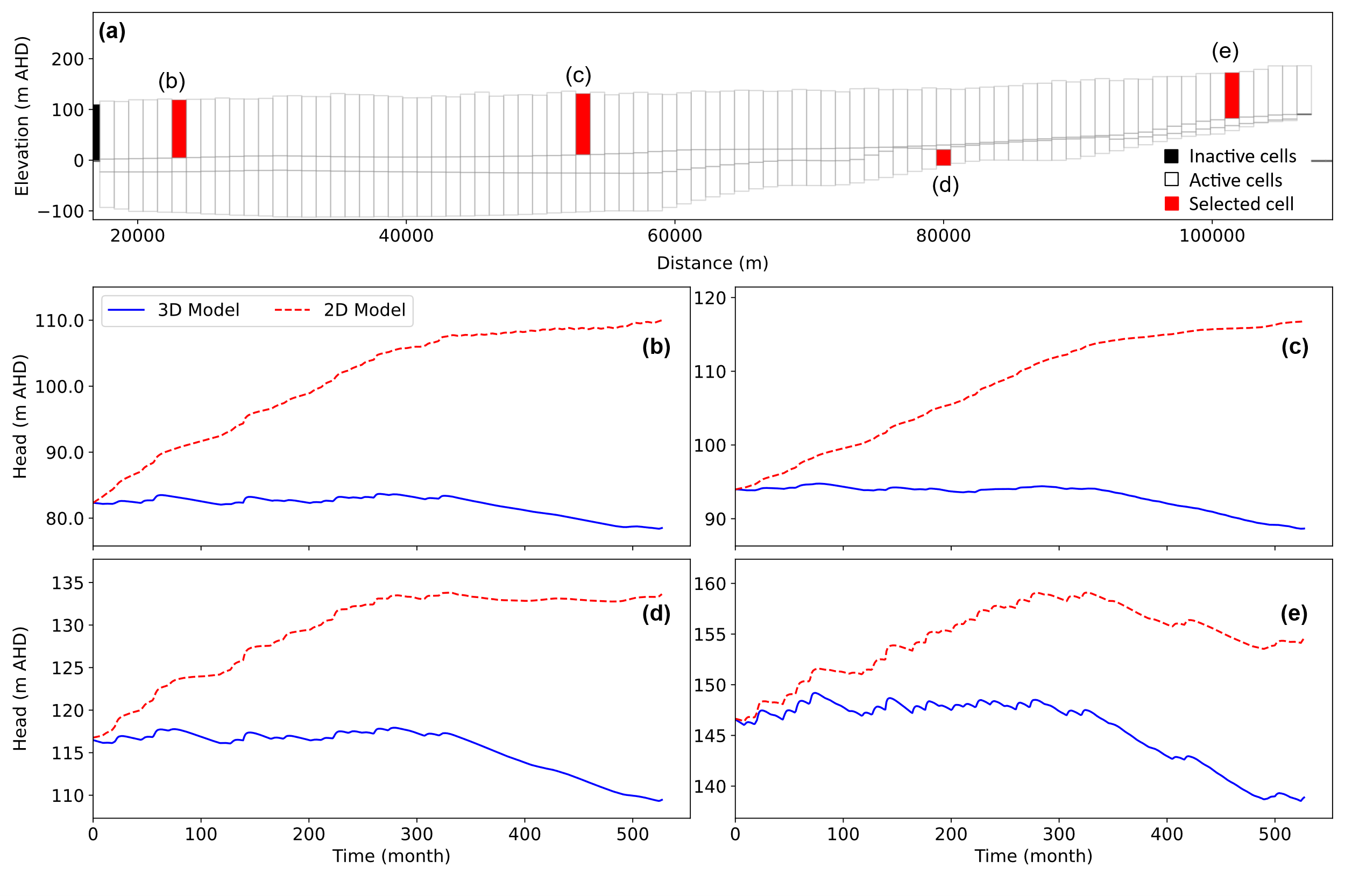


**Figure S18.** (a) Model grid of cross section II′, showing the location of selected cells for which hydrographs from both the 2D and 3D models are shown in (b), (c), (d) and (e).


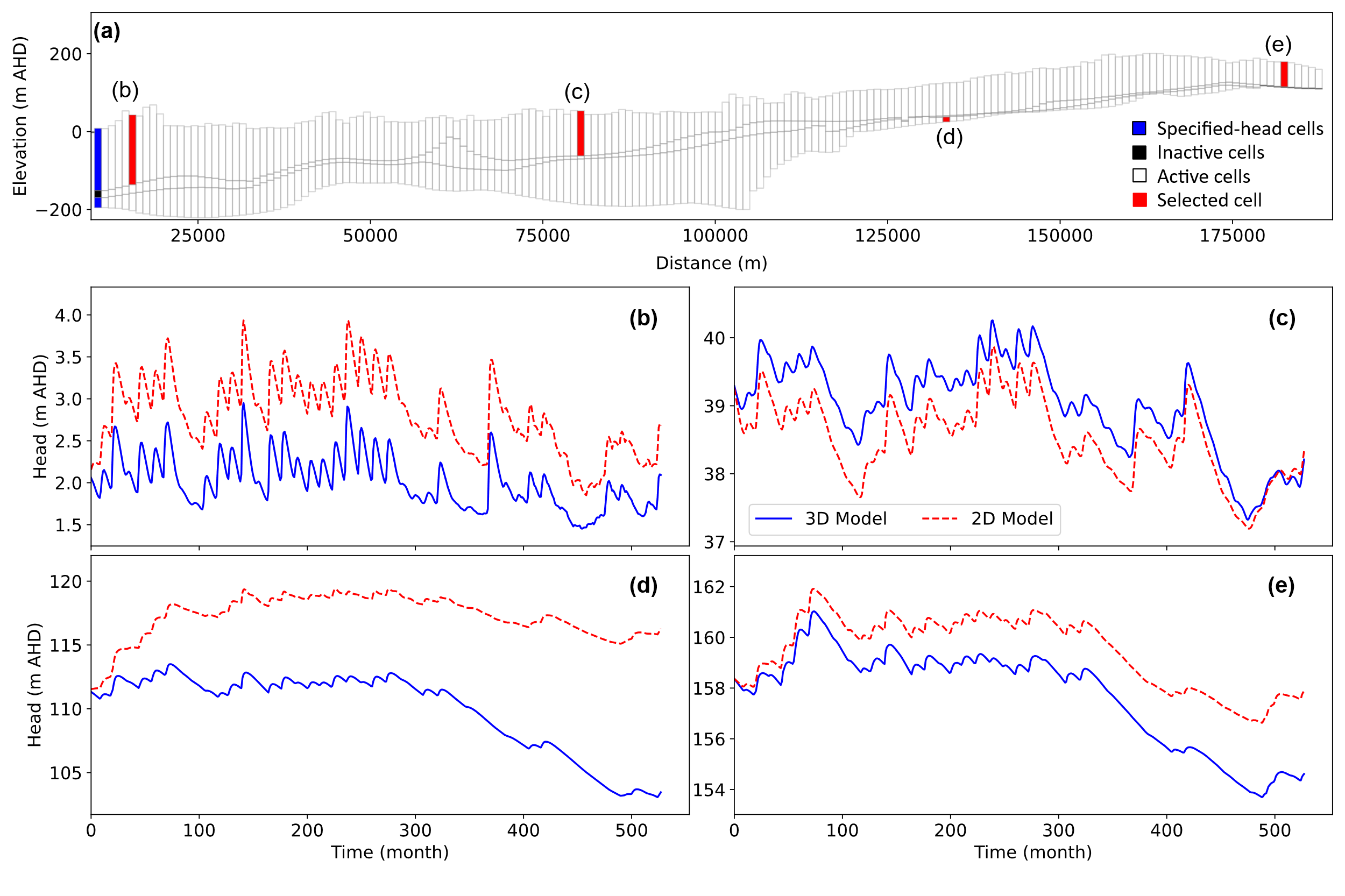


**Figure S19.** (a) Model grid of cross section JJ′, showing the location of selected cells for which hydrographs from both the 2D and 3D models are shown in (b), (c), (d) and (e).

**
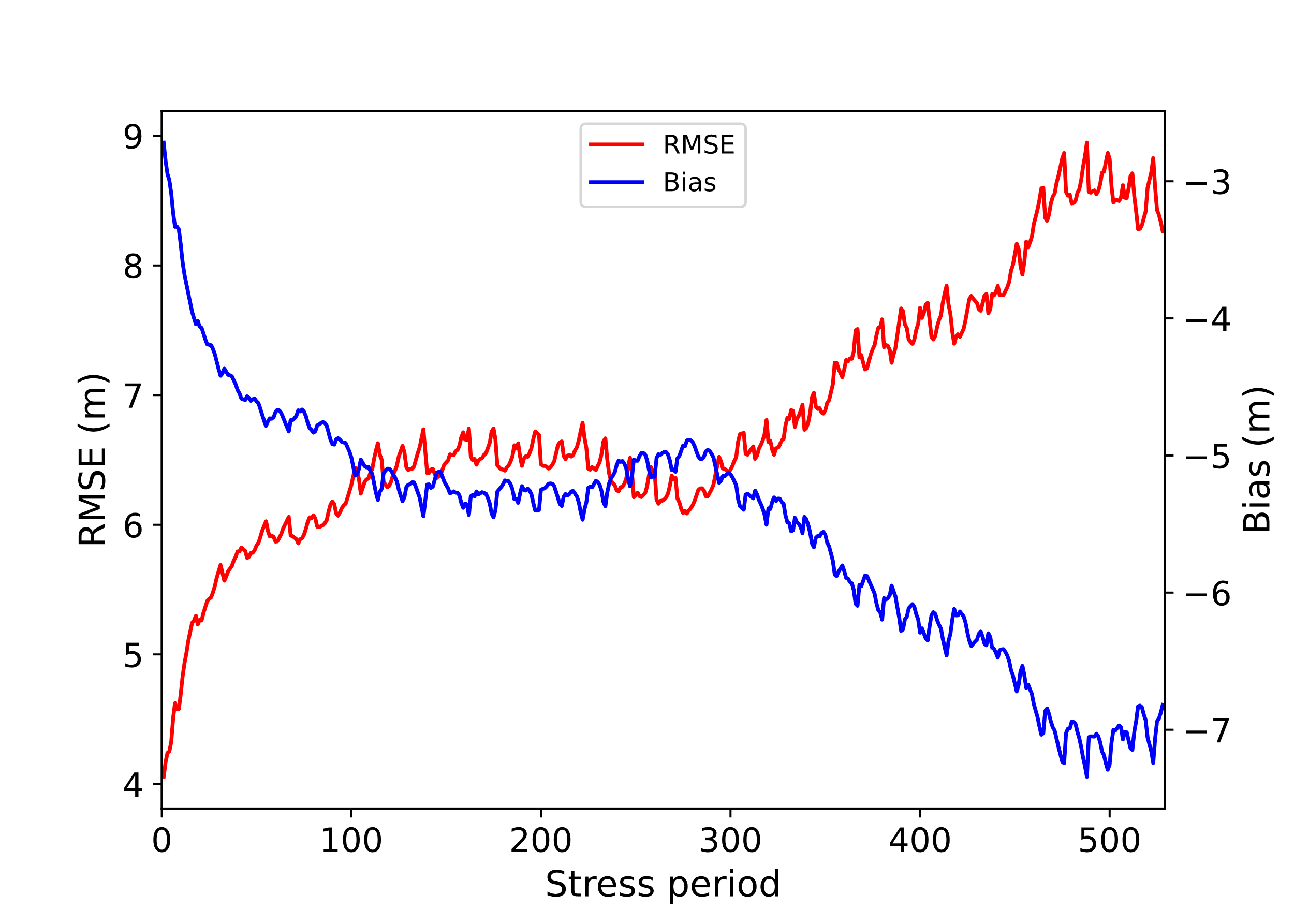
**

**Figure S20.** Temporal variation in the mean spatial error for cross section AA′.


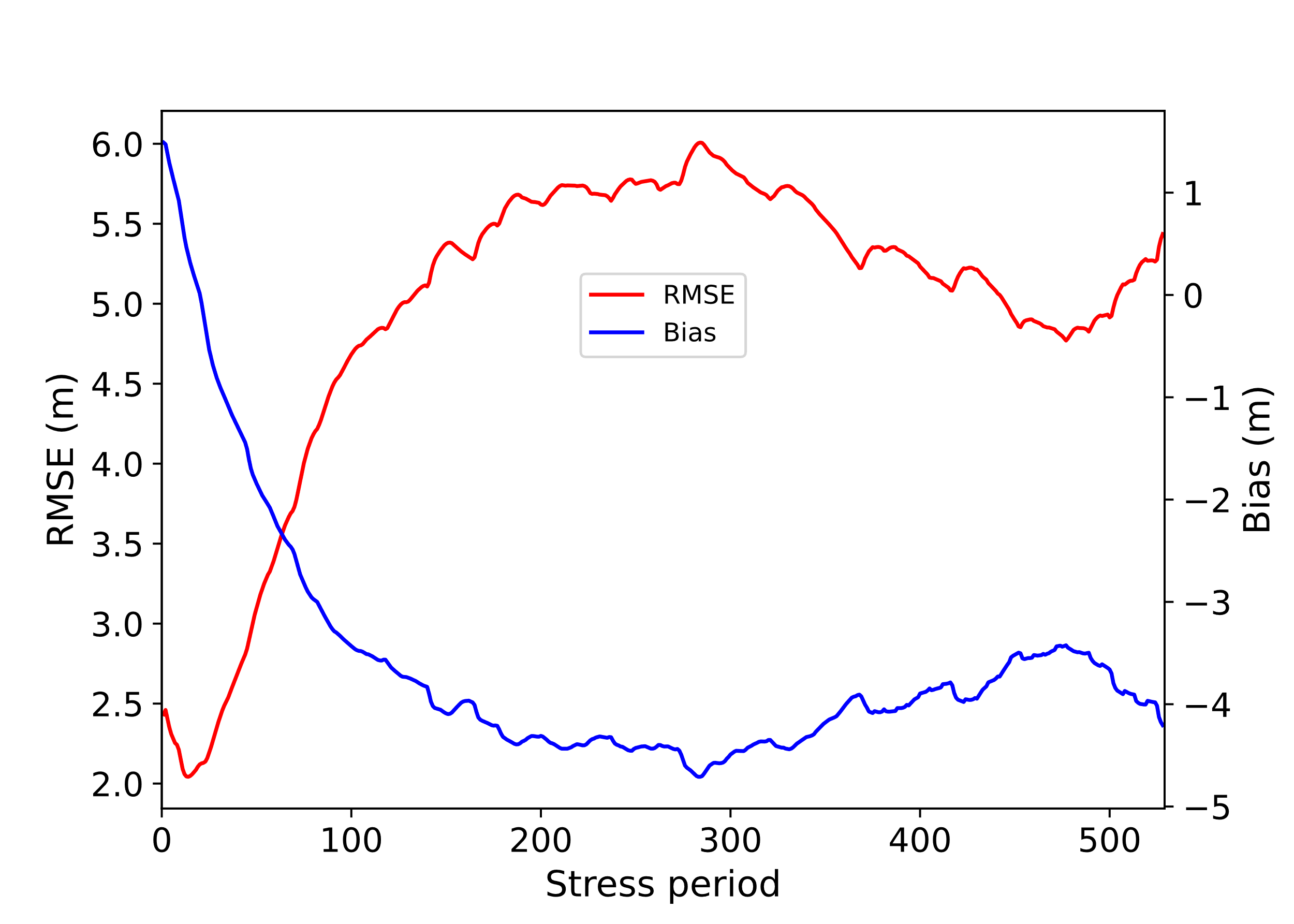


**Figure S21.** Temporal variation in the mean spatial error for cross section BB′.


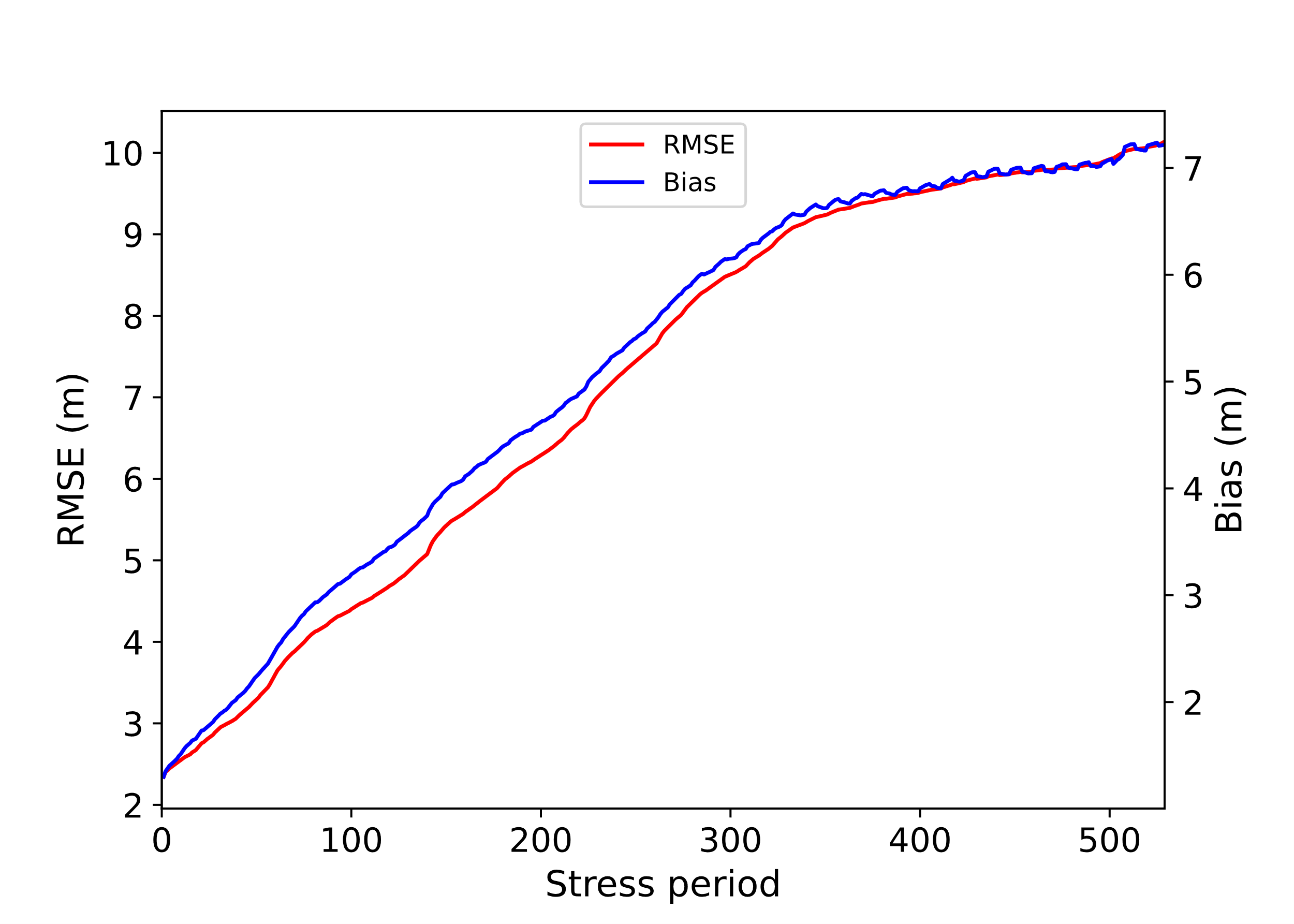


**Figure S22.** Temporal variation in the mean spatial error for cross section CC′.


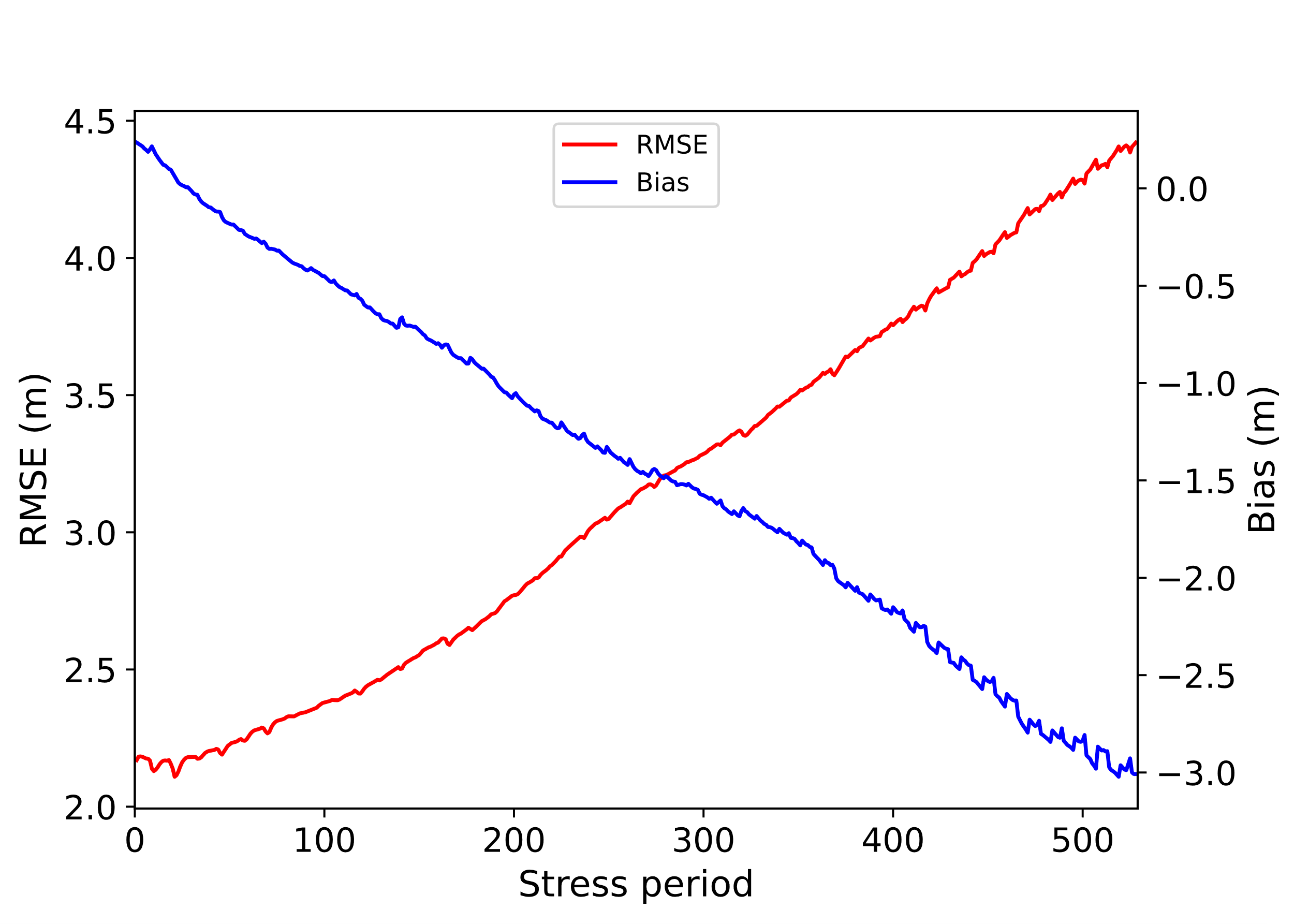


**Figure S23.** Temporal variation in the mean spatial error for cross section DD′.


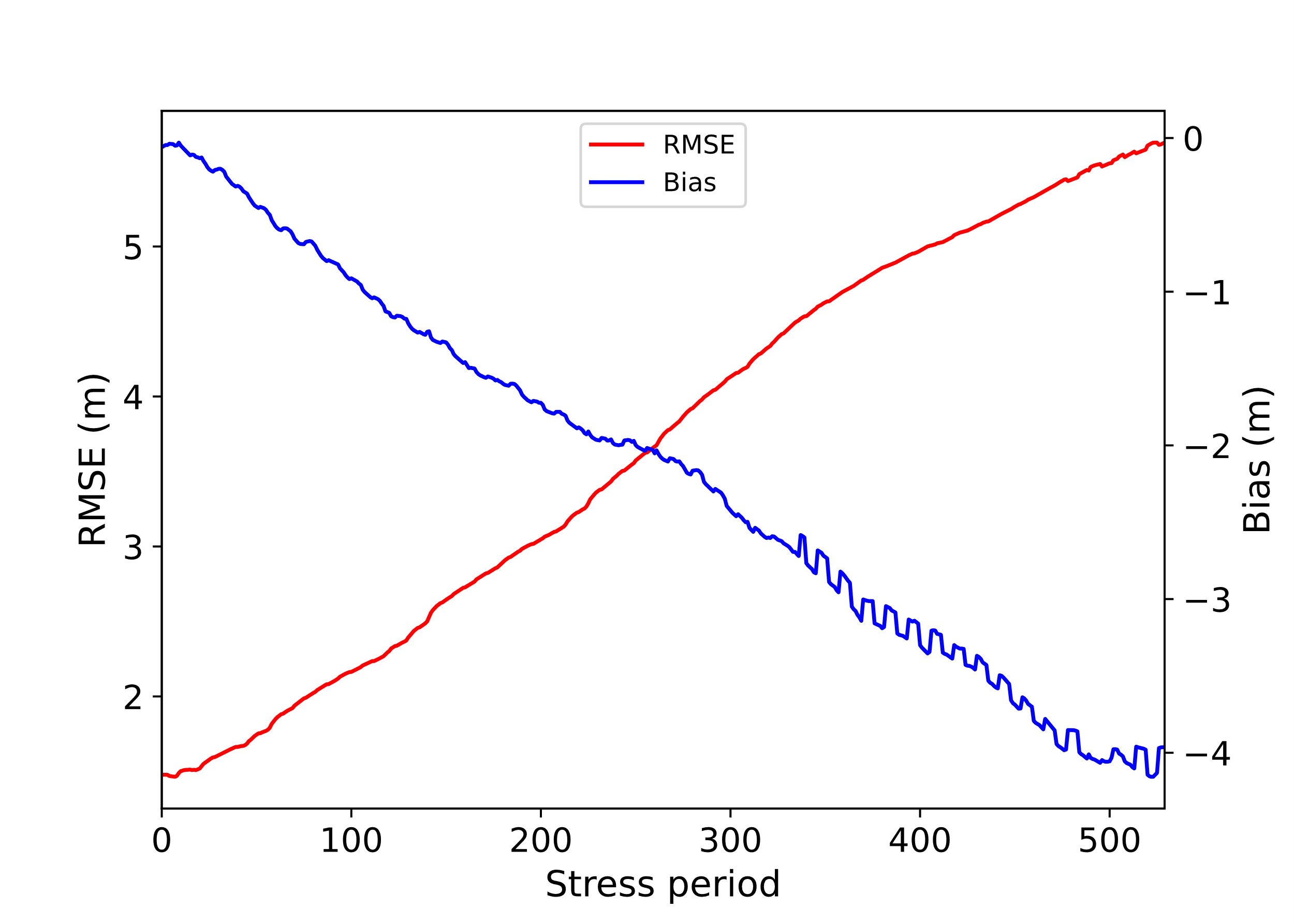


**Figure S24.** Temporal variation in the mean spatial error for cross section FF′.


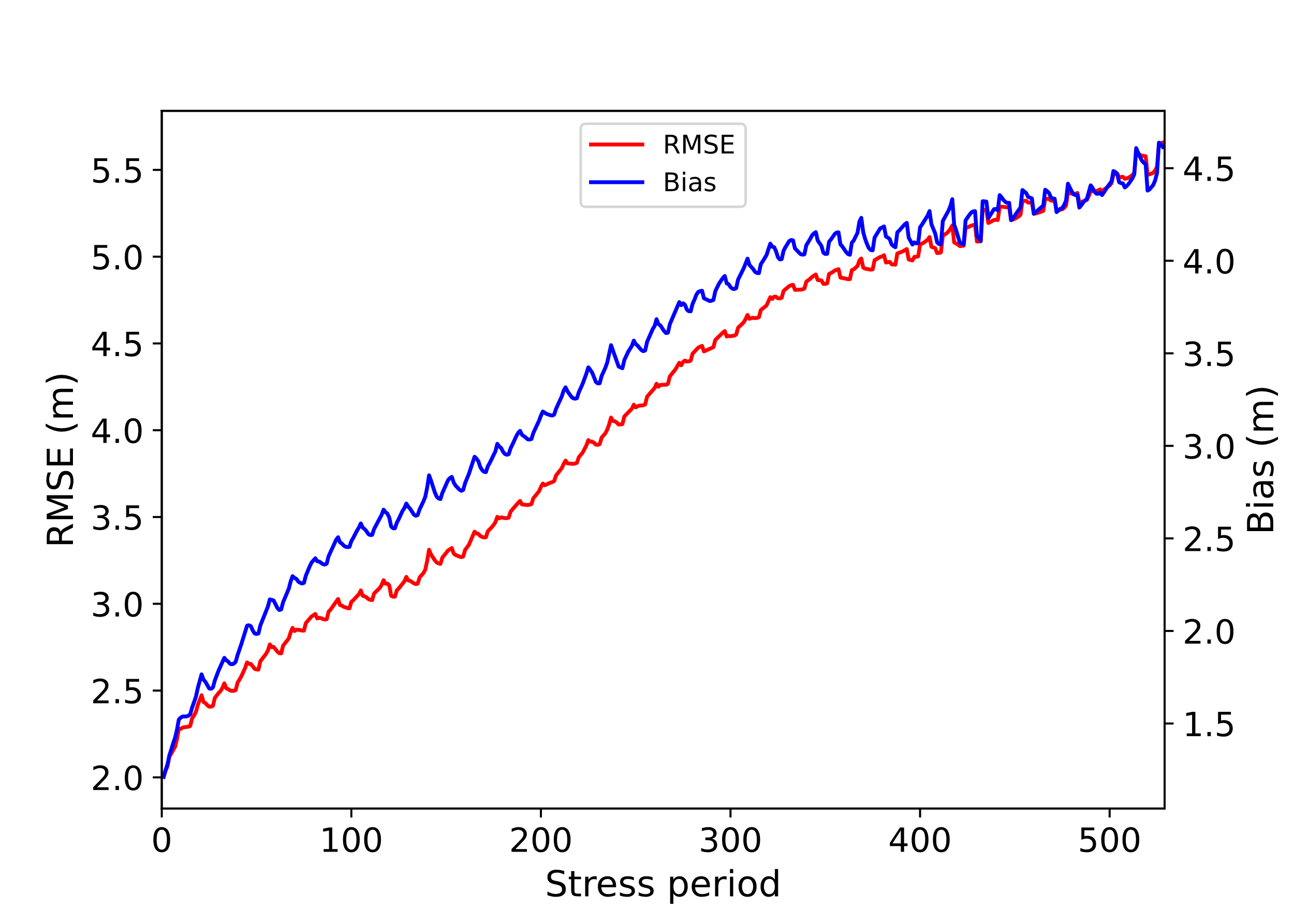


**Figure S25.** Temporal variation in the mean spatial error for cross section GG′.


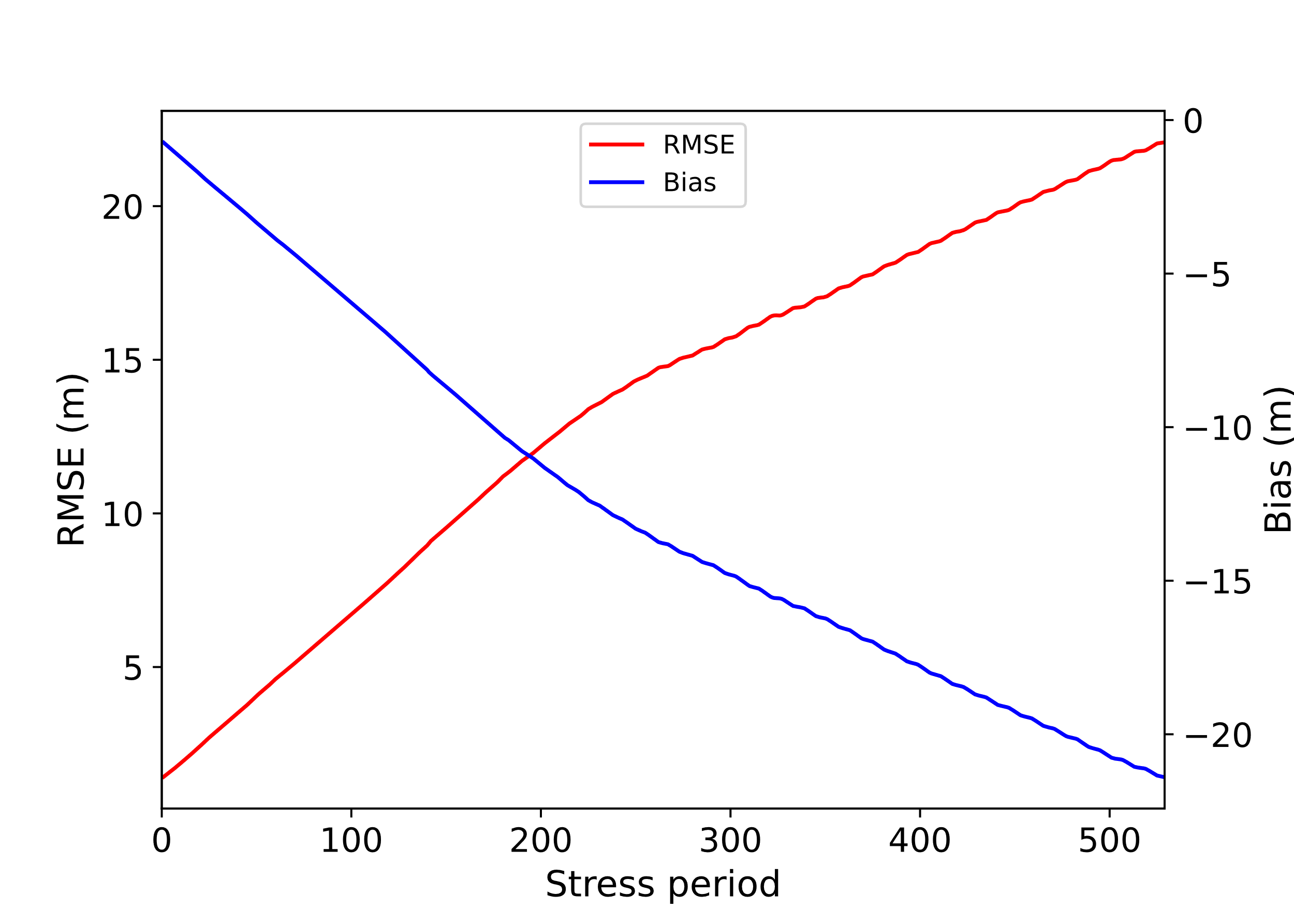


**Figure S26.** Temporal variation in the mean spatial error for cross section HH′.


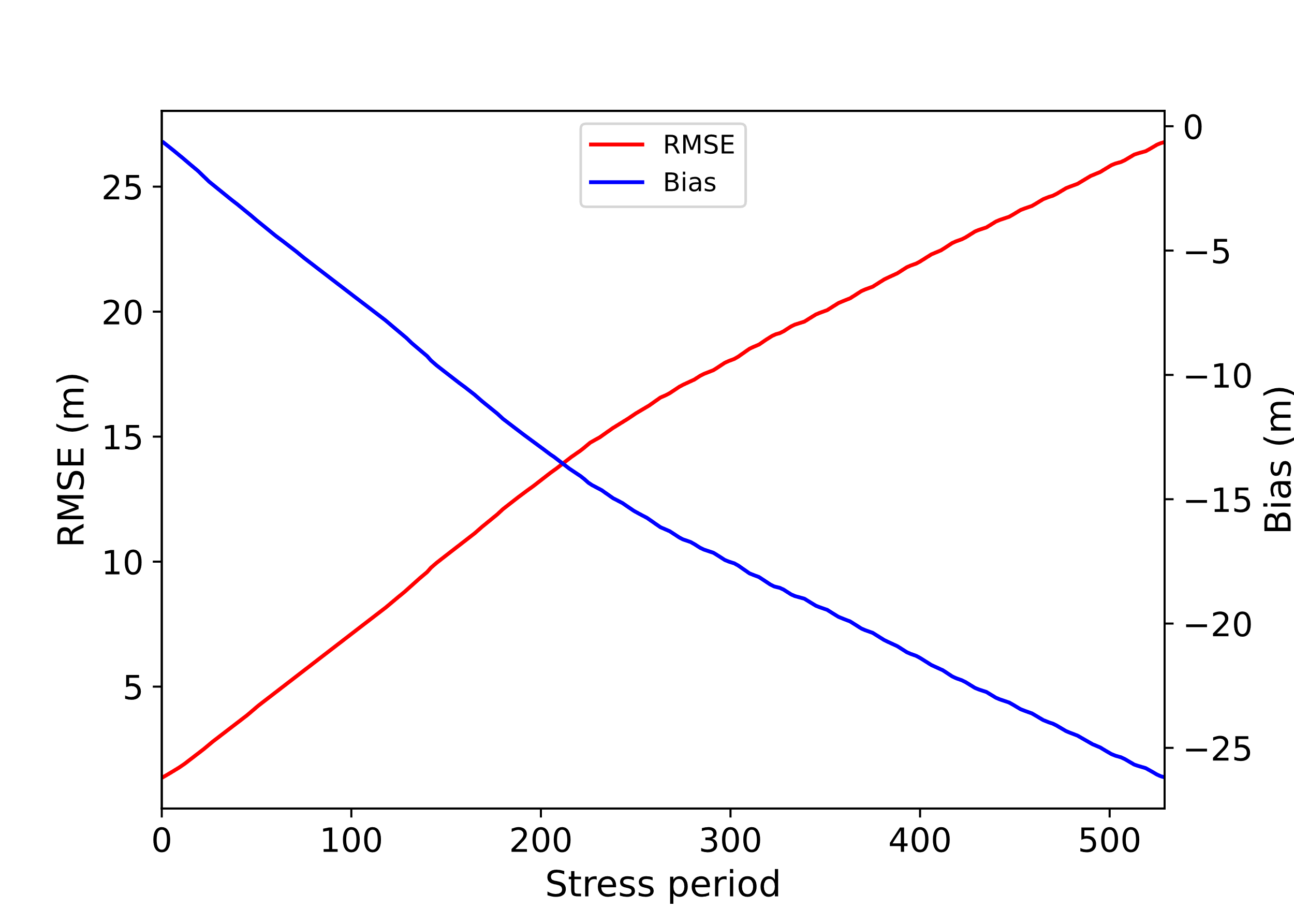


**Figure S27.** Temporal variation in the mean spatial error for cross section II′.


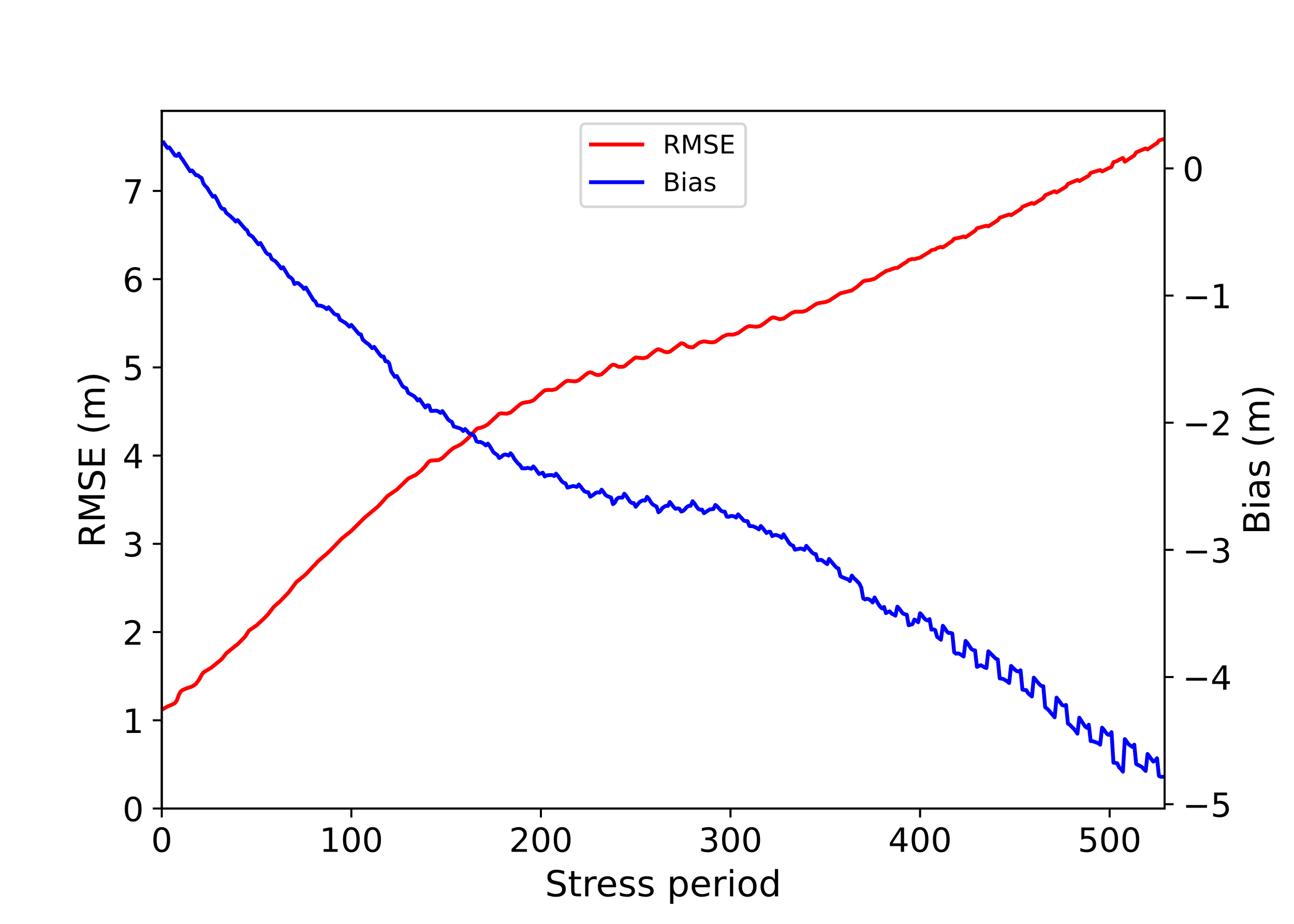


**Figure S28.** Temporal variation in the mean spatial error for cross section JJ′.


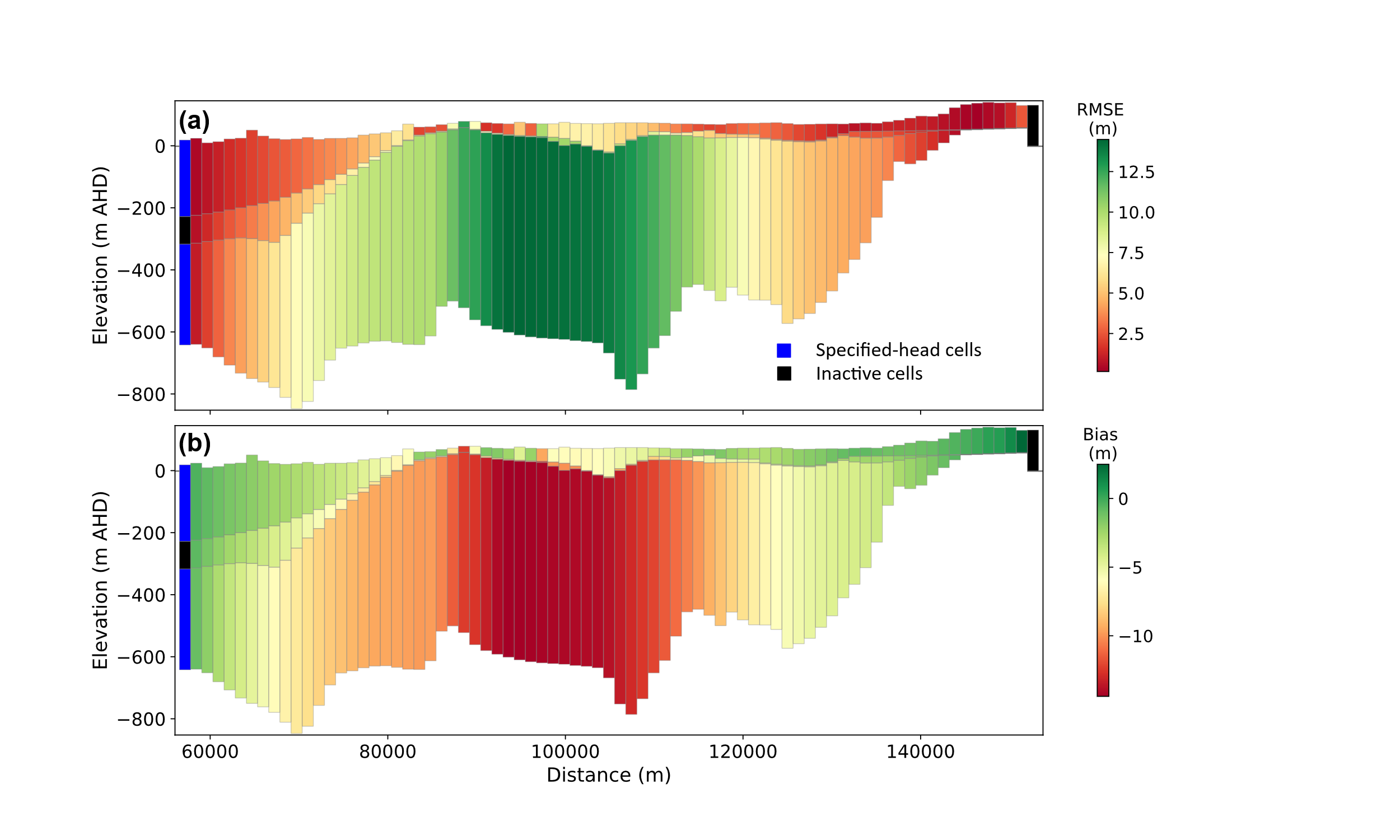


**Figure S29.** Spatial distribution of temporal error for cross section AA′: (a) RMSE, and (b) bias.


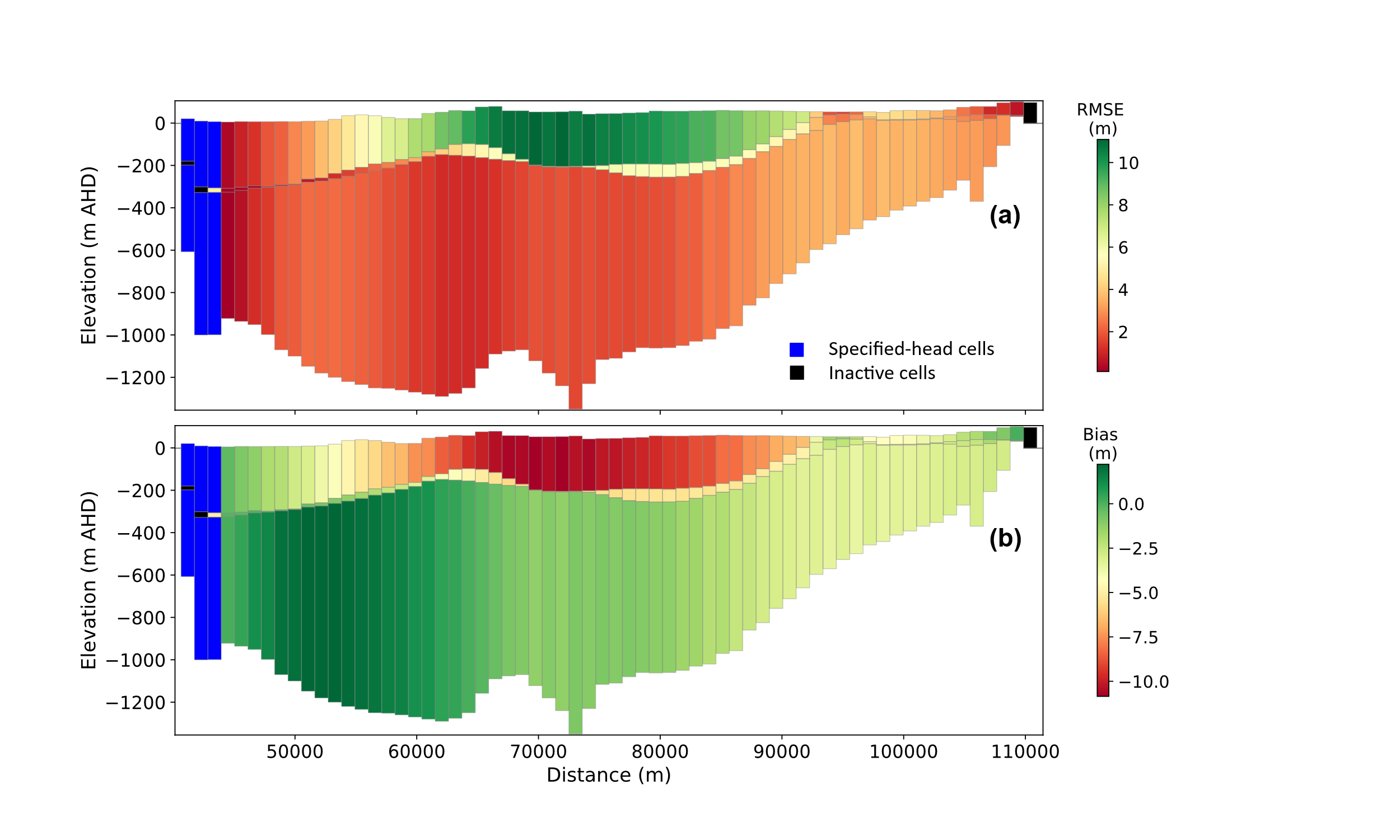


**Figure S30.** Spatial distribution of temporal error for cross section BB′: (a) RMSE, and (b) bias.

**
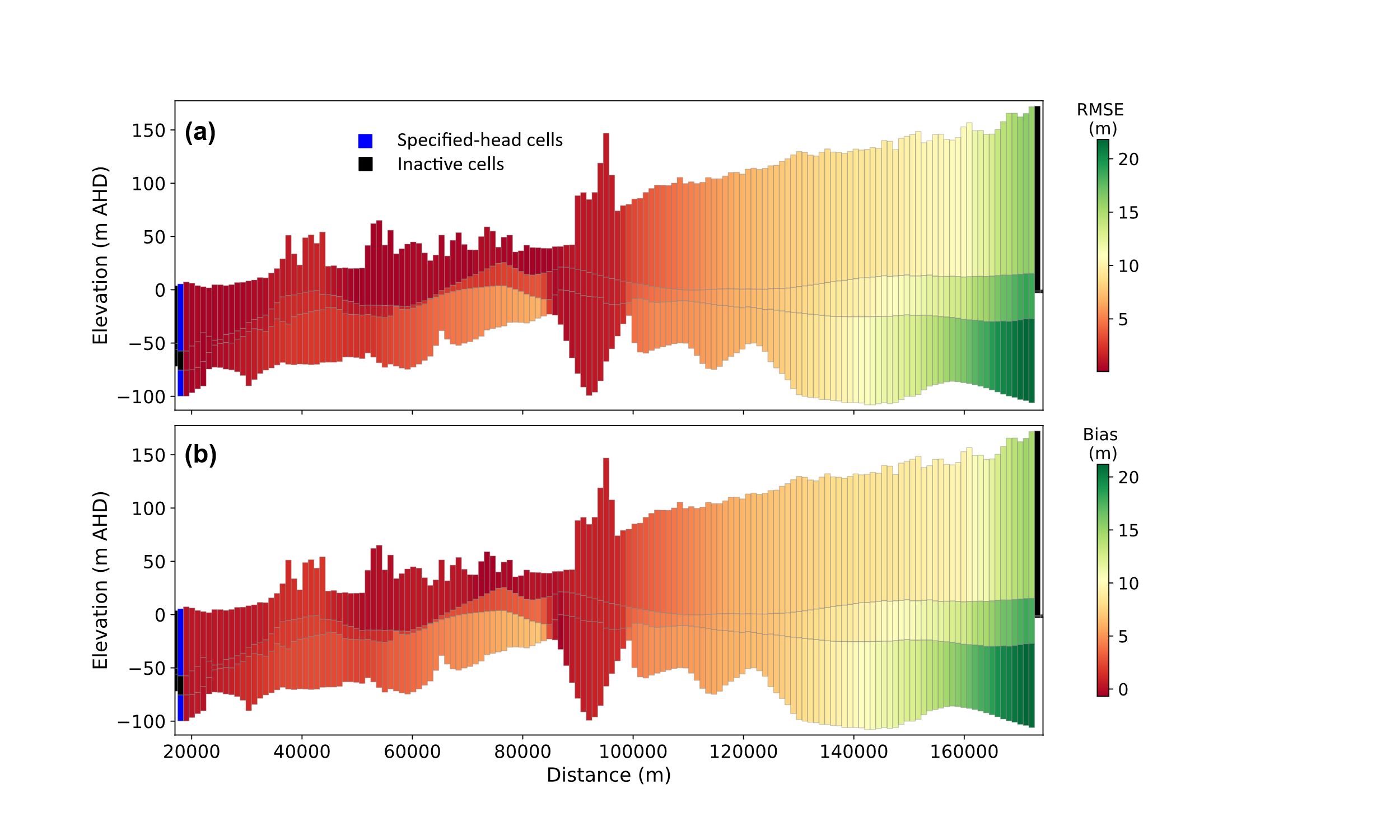
**

**Figure S31.** Spatial distribution of temporal error for cross section CC′: (a) RMSE, and (b) bias.


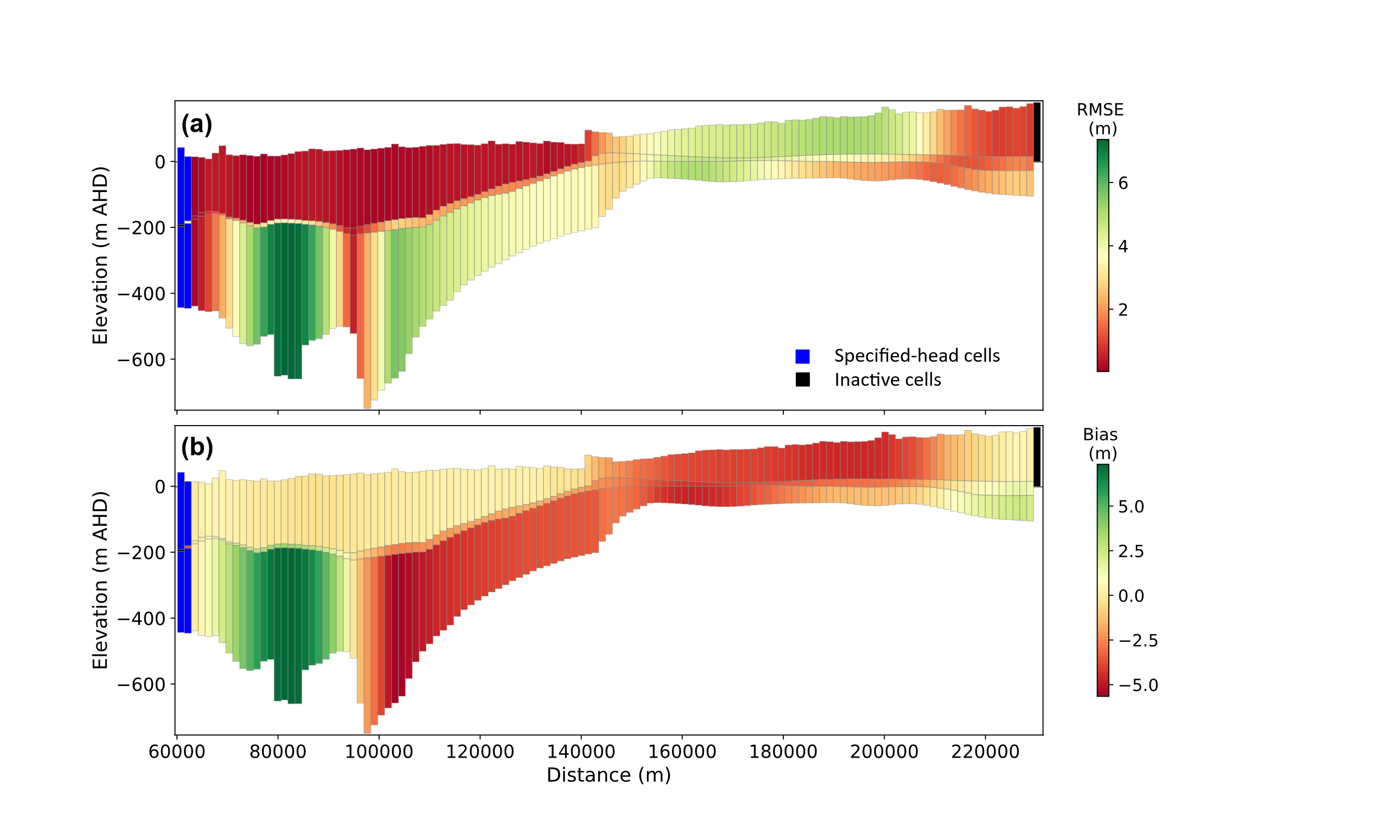


**Figure S32.** Spatial distribution of temporal error for cross section DD′: (a) RMSE, and (b) bias.


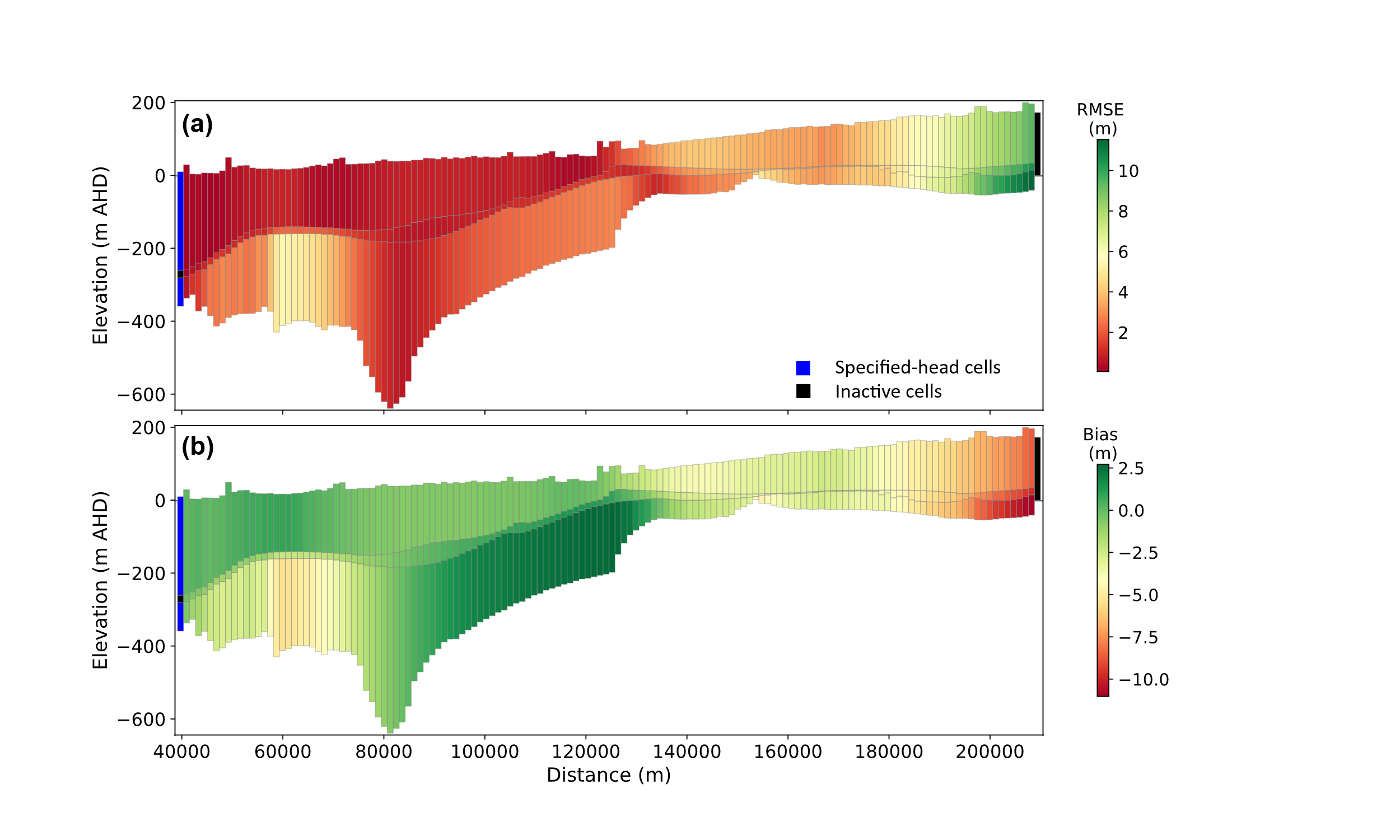


**Figure S33.** Spatial distribution of temporal error for cross section FF′: (a) RMSE, and (b) bias.


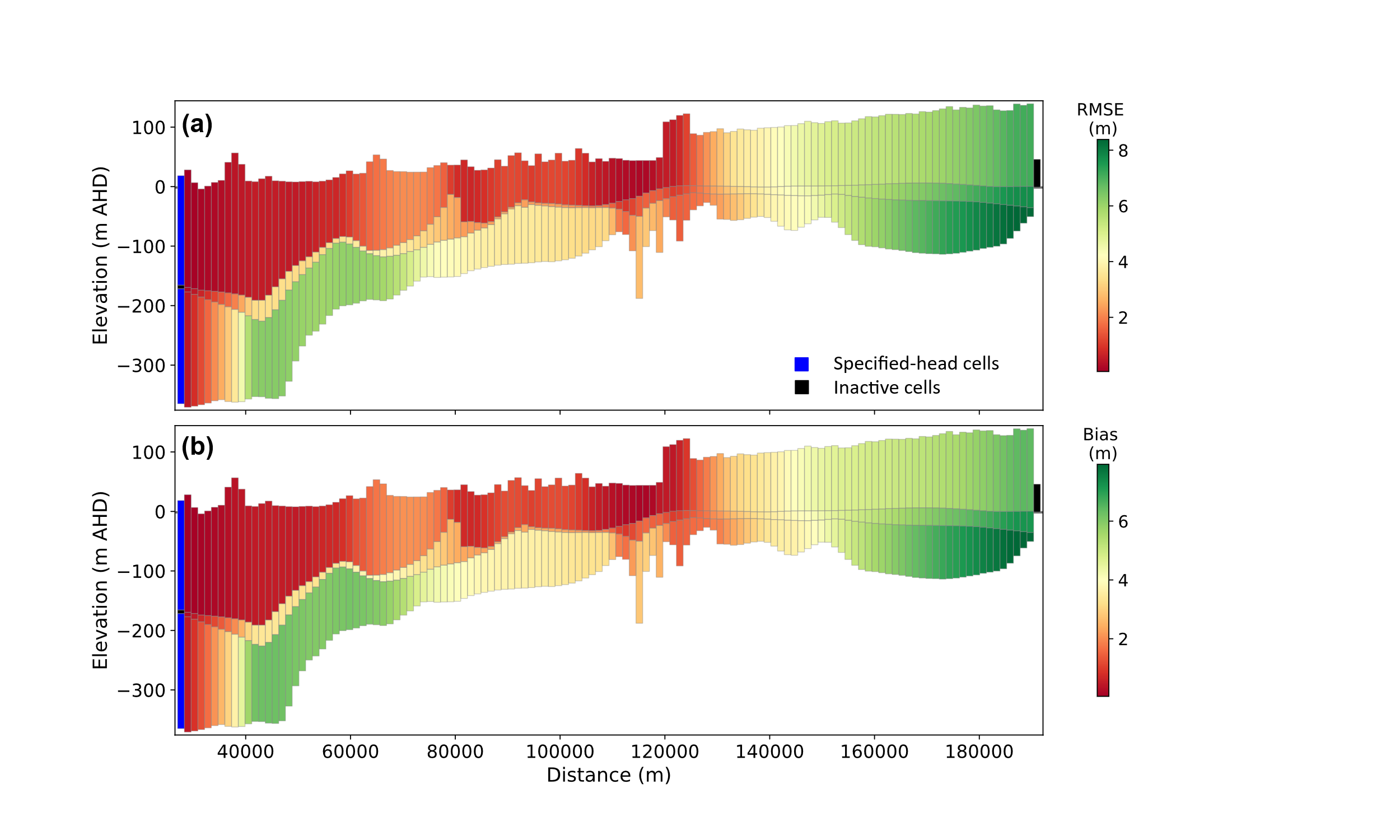


**Figure S34.** Spatial distribution of temporal error for cross section GG′: (a) RMSE, and (b) bias.


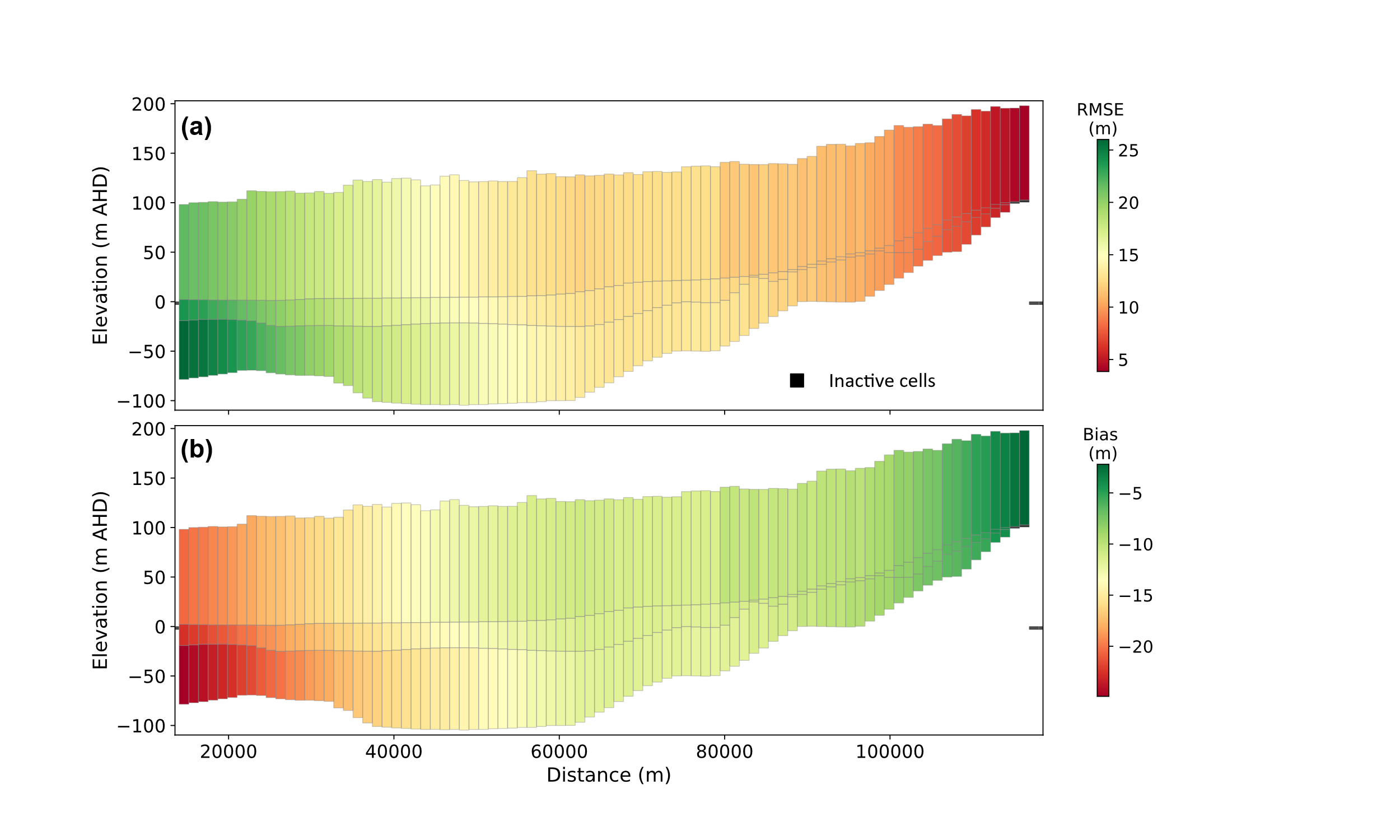


**Figure S35.** Spatial distribution of temporal error for cross section HH′: (a) RMSE, and (b) bias.


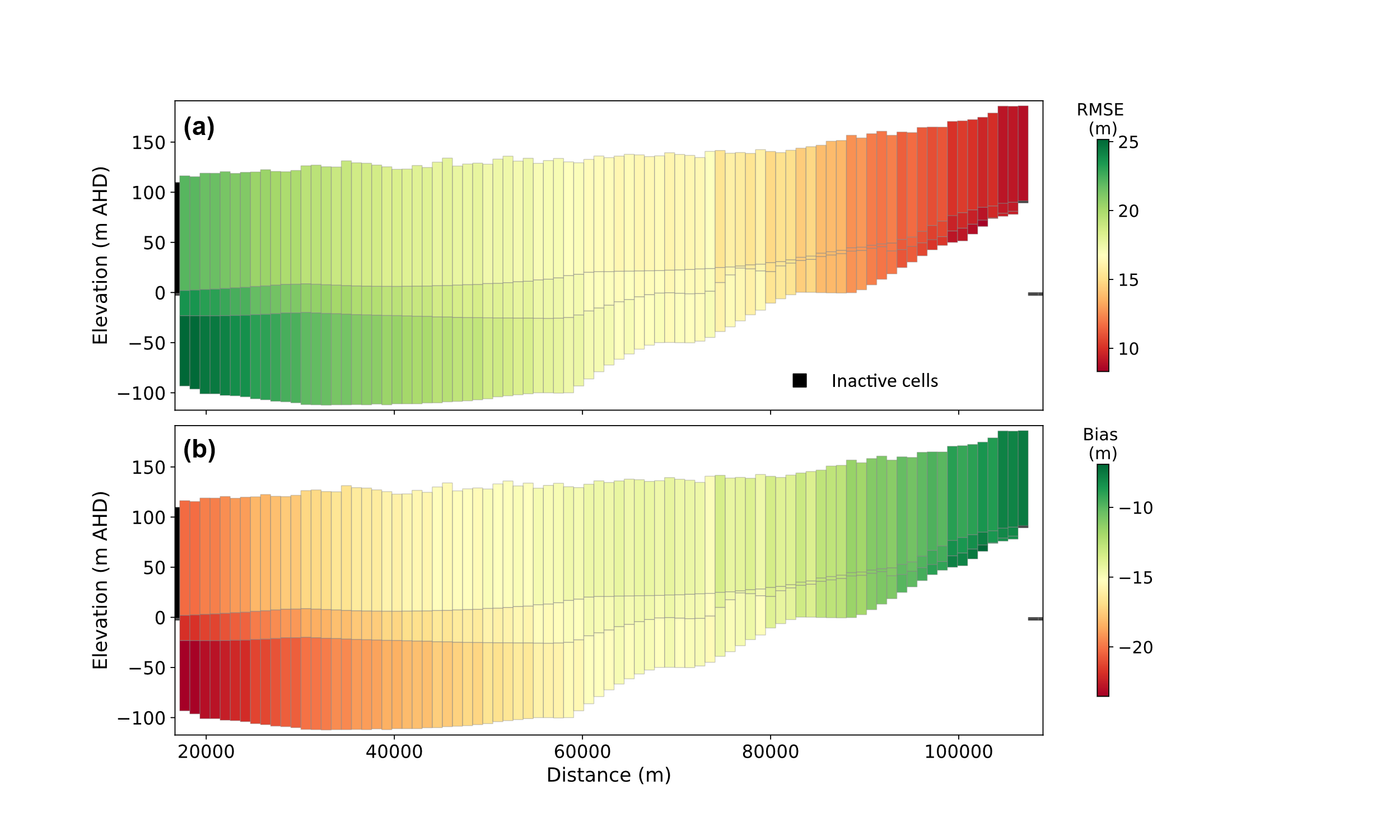


**Figure S36.** Spatial distribution of temporal error for cross section II′: (a) RMSE, and (b) bias.


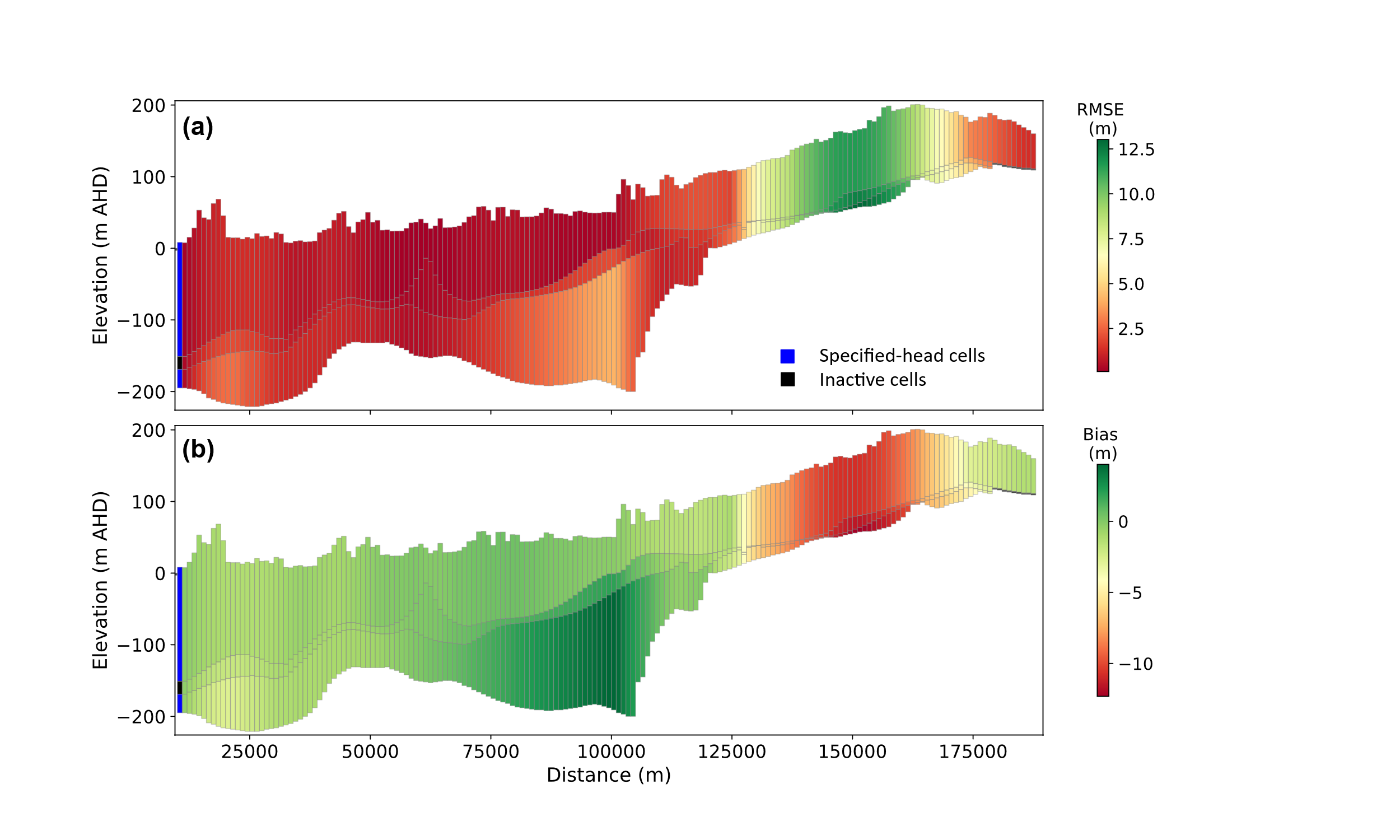


**Figure S37.** Spatial distribution of temporal error for cross section JJ′: (a) RMSE, and (b) bias.


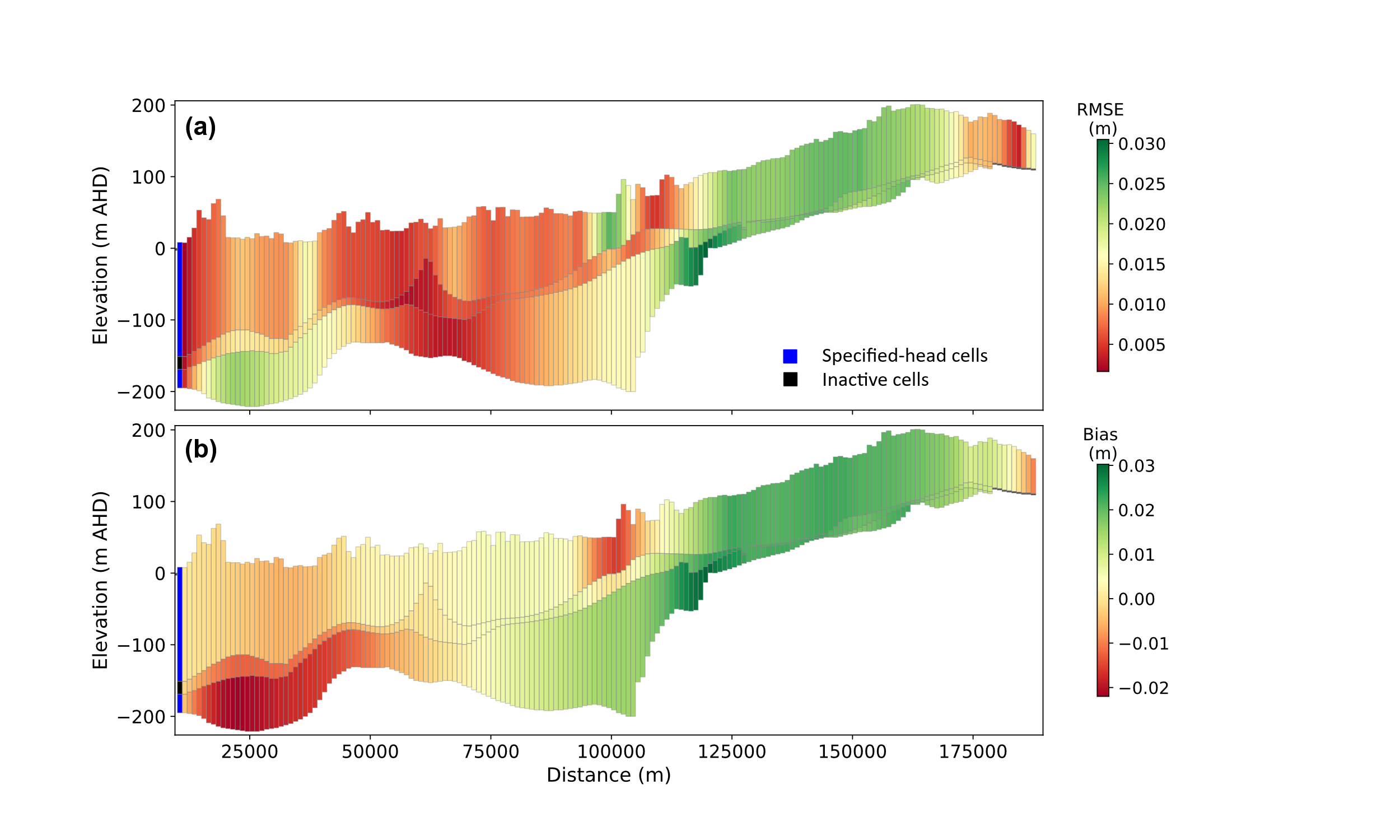


**Figure S38.** Spatial distribution of temporal error for cross section JJ′ with the addition of the lateral flow correction: (a) RMSE, and (b) bias.


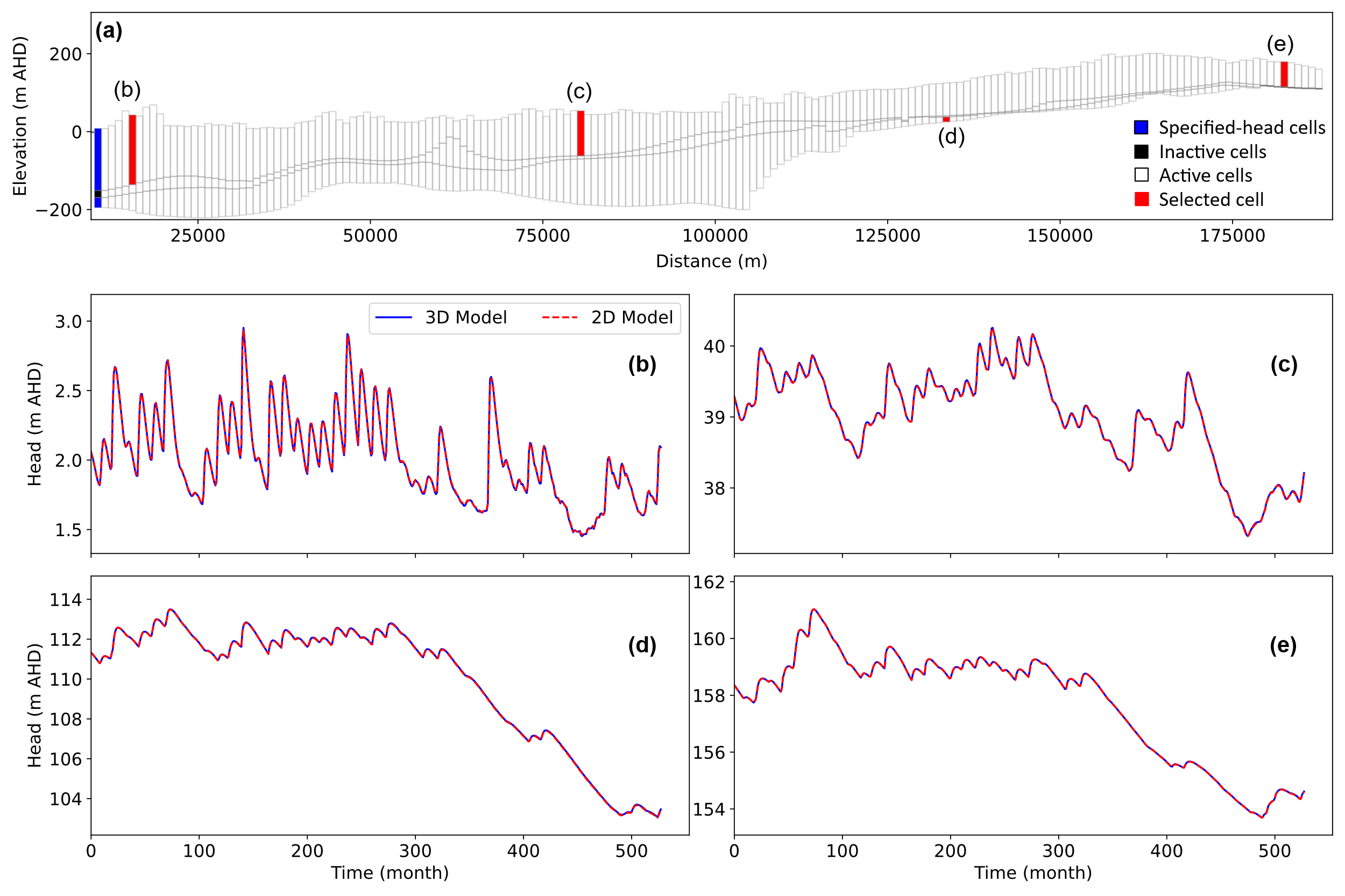


**Figure S39.** (a) Displays the grid of cross section JJ′, highlighting location of cells (b), (c), (d), and (e). (b), (c), (d), and (e) subsequently illustrate a comparison of head values from the 2D models (dashed red lines) and 3D models (solid blue lines) within these highlighted cells, specifically when lateral flow is incorporated into cross section JJ′.


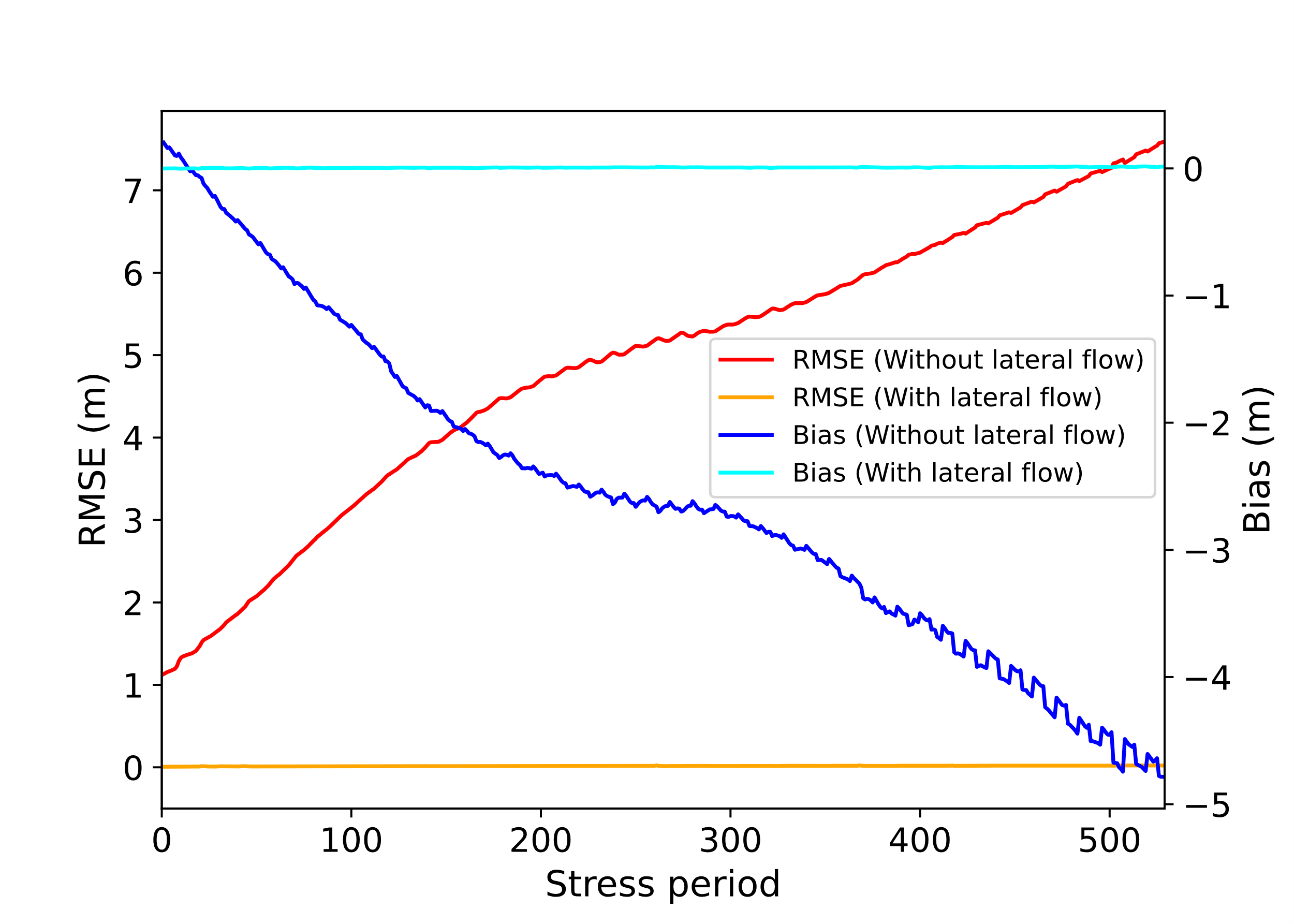


**Figure S40**. Comparison between variation of mean spatial error over time for cross section JJ′ with and without the addition of the lateral flow correction.


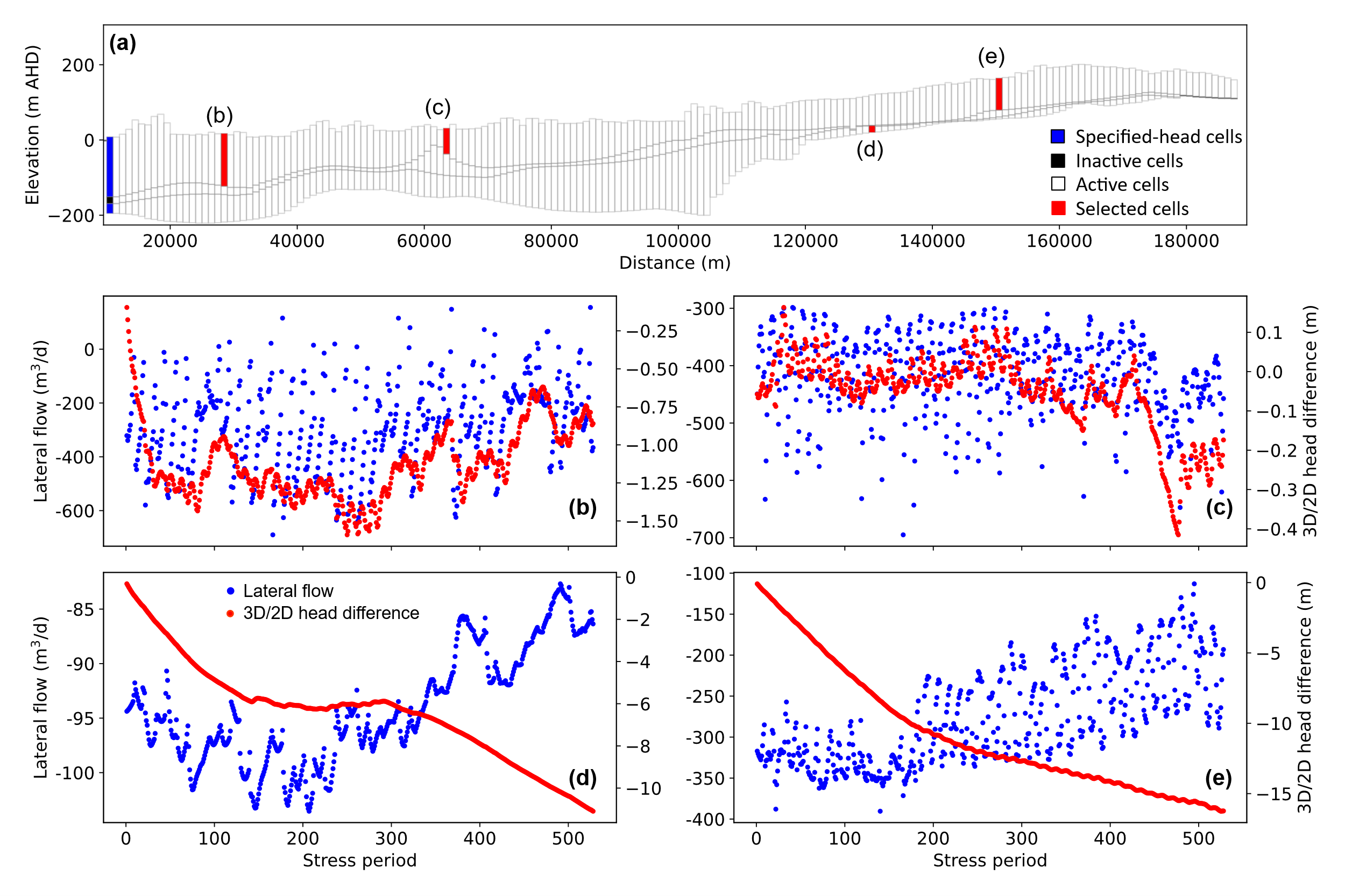


**Figure S41.** (a) Model grid of cross section JJ′, showing the locations of selected cells for which changes in lateral flow and 3D/2D head differences versus stress period are displayed in (b), (c), (d), and (e).


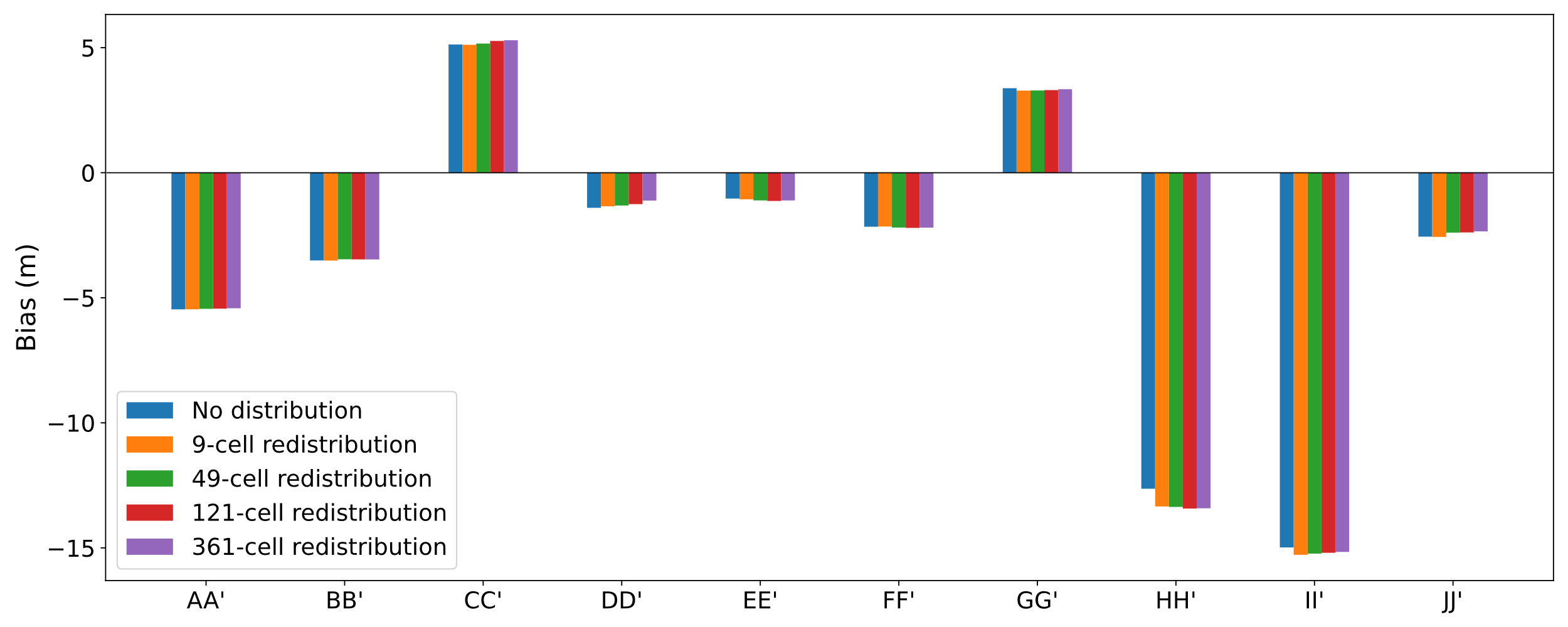


**Figure S42.** Comparison of bias arising from different pumping redistribution scenarios.


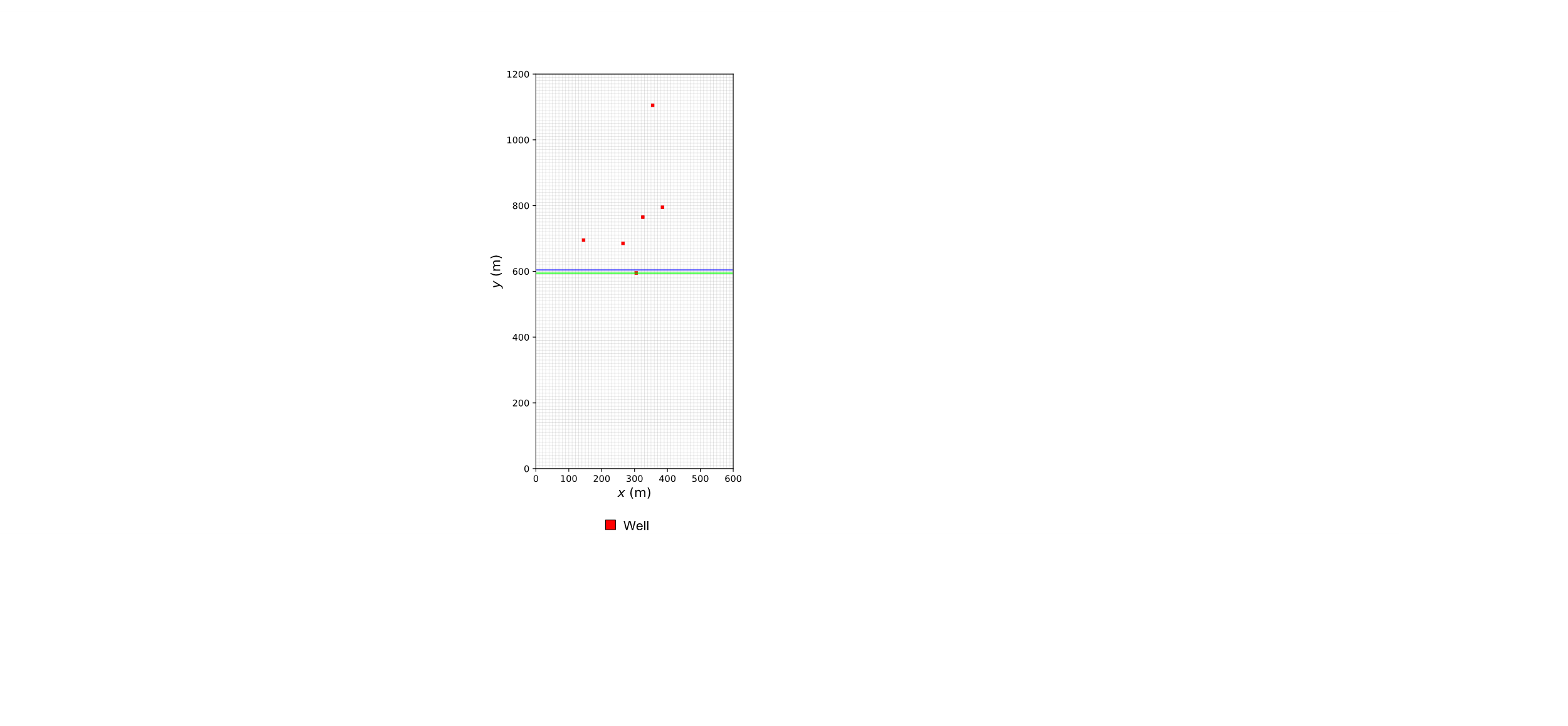


**Figure S43.** Map view of simple 3D model. The solid green line represents a cross section that intersects the cell containing the well, while the solid blue line indicates a cross section that is one cell away from the cell with the well.


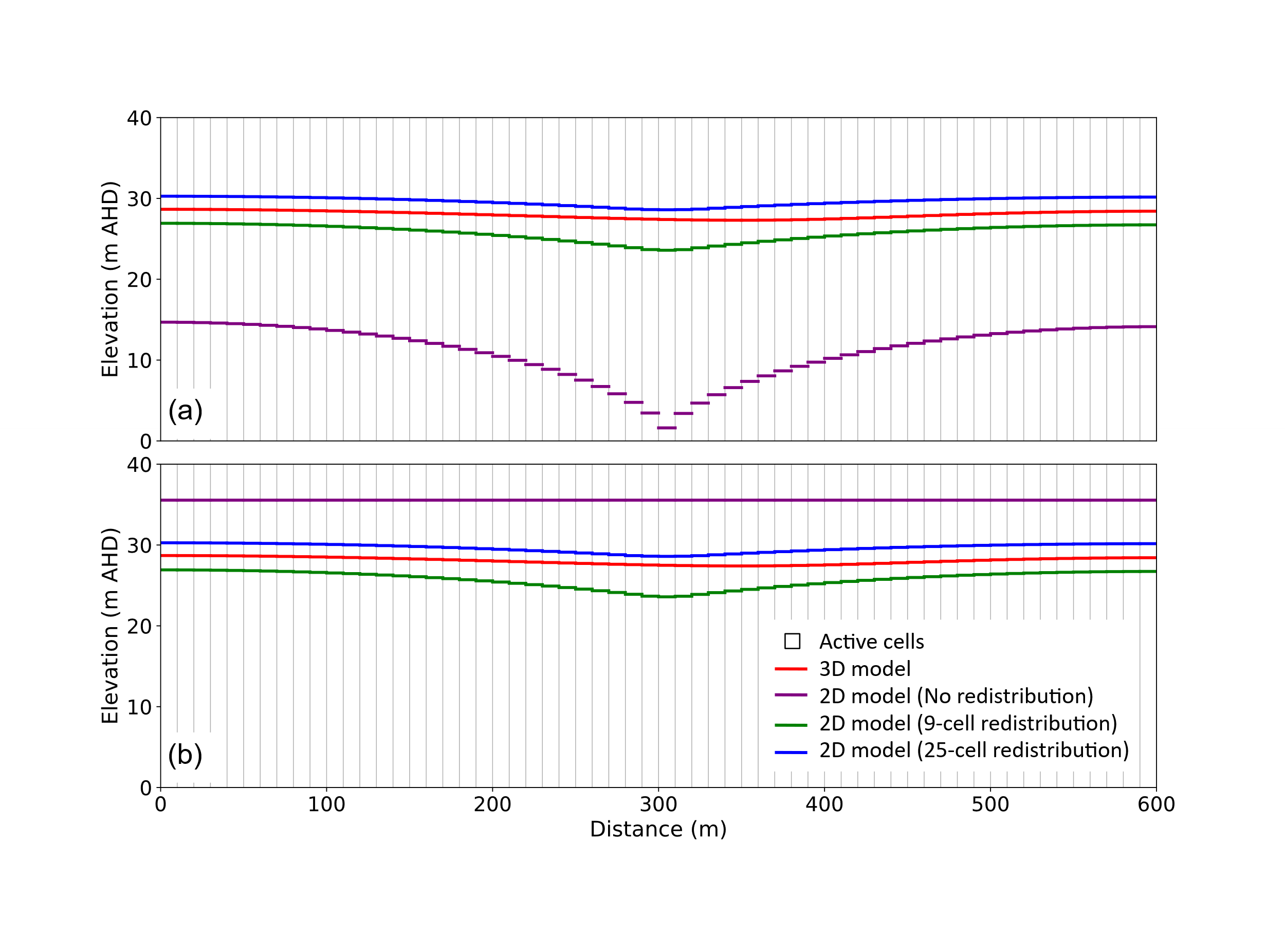


**Figure S44.** Impact of redistribution on head matching between 2D and 3D models: (a) cross section intersects the cell which has pumping well (solid green line in Figure S43), and (b) cross section is one cell away from cell which has pumping well (solid blue line in Figure S43).
